# Immune reprogramming in the bone marrow microenvironment: a new perspective on the bone immune microenvironment of postmenopausal osteoporosis

**DOI:** 10.3389/fimmu.2026.1766460

**Published:** 2026-02-27

**Authors:** Dingpeng Li, Xianli Zheng, Deming Lin, Yuan Cheng, Zhong Wang, Yangyang Chen, Xingwen Xie

**Affiliations:** 1 Gansu University Of Chinese Medicine, Lanzhou, Gansu, China; 2 Gansu Provincial Second People’s Hospital, Lanzhou, Gansu, China; 3 The Affiliated Hospital of Gansu University of Chinese Medicine, Lanzhou, China

**Keywords:** postmenopausal osteoporosis, bone marrow microenvironment, immune reprogramming, bone immunology, inflammatory bone loss, bone immune microenvironment therapy

## Abstract

Research on postmenopausal osteoporosis (PMOP), a common bone metabolic disease, has traditionally focused on bone loss and imbalance in bone remodeling. However, with the development of bone immunology, the complex interactions between immune cells and bone cells in the bone marrow microenvironment have gradually been revealed, and “immune reprogramming” is considered a key factor driving the persistent bone loss in PMOP. Current evidence indicates that the postmenopausal bone marrow microenvironment undergoes significant structural and functional changes. These changes are characterized by a myeloid bias in hematopoietic stem/progenitor cells, aging of bone marrow mesenchymal stem cells (BMSCs) with a tendency toward differentiation into the adipocyte lineage, an imbalance of key immune cell subpopulations such as M1 and M2 macrophages and Th17 and regulatory T cells (Treg), as well as remodeling of cytokine and chemokine axis networks. Signaling pathways such as RANK/RANKL/OPG, Wnt/β-catenin, CXCL12–CXCR4, and S1P — along with systemic factors like estrogen deficiency, inflammatory aging, and the gut-bone-immune axis-collectively shape the characteristic bone immune microenvironment of PMOP. Based on this, this article systematically reviews the changes in cell lineage and molecular mechanisms underlying PMOP bone marrow immune reprogramming. It focuses on the key signaling networks in the bone immune microenvironment and their relationship with the mechanisms of existing anti-osteoporosis drugs. Furthermore, it proposes an immunotherapy approach represented by a three-tiered framework: traditional bone-targeted drugs, immune-guided therapy, and comprehensive intervention of the bone marrow microenvironment. Finally, in conjunction with emerging technologies such as multi-omics, single-cell, and spatial omics, this article discusses future directions for constructing a PMOP bone immune map and achieving precise stratification and individualized intervention, aiming to provide a theoretical basis and methodological reference for mechanistic research and bone immune-targeted therapy of PMOP.

## 1 Introduction

Postmenopausal osteoporosis (PMOP) is a common metabolic bone disease affecting middle-aged and elderly women, characterized primarily by decreased bone mass and damaged bone microstructure. This condition leads to a significantly increased risk of fragility fractures and severely impairs the quality of life and prognosis of elderly women (1). The global trend of population aging has led to a rising incidence of PMOP, imposing a heavy burden on individual and public health (2). Although traditional views attribute PMOP mainly to the decline in estrogen levels after menopause, resulting in an imbalance in bone remodeling, focusing solely on bone mass and bone remodeling does not fully explain its complex pathogenesis (3). In recent years, bone immunology, a frontier discipline at the intersection of bone metabolism and the immune system, has revealed the important role of the immune system in regulating bone metabolism, providing new insights into understanding PMOP (4, 5).

The bone marrow microenvironment is a complex system in which immune cells coexist with bone cells. Immune cells participate in regulating bone formation and resorption through the secretion of cellular factors and by direct cell-to-cell contact (6). After menopause, significant changes occur in the bone marrow immune microenvironment, characterized by an enhanced pro-inflammatory state and alteration of immune cell composition and function. This promotes the activity of bone resorption cells—osteoclasts—and inhibits osteoblastic function, leading to bone loss (7, 8). Notably, the proportion of Treg decreases in the peripheral blood and some bone marrow samples studied from postmenopausal women, while pro-inflammatory Th17 cells increase, disrupting immune balance. Released inflammatory factors, such as TNF-α and IL-17, promote the differentiation and activation of osteoclasts, exacerbating bone resorption (9, 10). It is important to emphasize that changes in the inflammatory/immune profile in peripheral blood do not necessarily equate to alteration of the bone marrow microenvironment. Subsequent sections will focus on direct evidence from the bone marrow of PMOP patients and supplement this with analogous evidence from ovariectomized animal (OVX) models.

The remodeling of the bone marrow immune microenvironment not only affects the functions of bone cells but also promotes the biased generation of myeloid cells by regulating the differentiation lineage of bone marrow hematopoietic stem cells/progenitor cells (HSCs/HSPCs), thereby exacerbating the inflammatory state and forming a vicious cycle of bone metabolism (11). Studies have shown that there has been upregulation of inflammation-related gene expression and abnormal signaling between immune cells in the bone marrow samples of PMOP patients, indicating that the bone marrow microenvironment exhibits pro-inflammatory characteristics (12). The polarization states of macrophages, dendritic cells, and other immune cells in the bone marrow also change, with an increase in pro-inflammatory M1 macrophages and a decrease in anti-inflammatory M2 macrophages, further aggravating bone metabolic disorders (13). At the same time, mechanisms such as oxidative stress, abnormal iron metabolism, and apoptosis in the bone marrow immune microenvironment have also been confirmed to participate in the immune alteration process of PMOP (14). Furthermore, the application of single-cell transcriptomics technology has further revealed the heterogeneity and functional diversity of bone marrow immune cells, providing technical support for a deeper understanding of immune reprogramming (15, 16). Therefore, systematically sorting out the dynamic changes of immune cells and their molecular mechanisms in the bone marrow immune microenvironment and revealing the restructuring characteristics of bone marrow structure and function are of great significance. Clarifying the roles of key immune cells and signaling pathways in PMOP is essential for understanding disease mechanisms and developing precise treatment strategies.

This article systematically reviews the structural and compositional changes in the bone immune microenvironment of PMOP, the characteristics of immune reprogramming, and its key signaling networks within the framework of bone immunology. It focuses on the potential role of estrogen decline leading to increased inflammatory factors and immune dysregulation, which in turn amplify bone resorption primarily through the RANK/RANKL/OPG pathway, while also affecting the Wnt/β-catenin signaling involved in bone formation during inflammatory bone loss. Additionally, the article evaluates the mechanisms of action of existing drugs targeting the bone immune microenvironment and emerging immune-targeted strategies. These evaluations are discussed in conjunction with the latest advances in multi-omics and single-cell technologies, providing theoretical support for the precise diagnosis and treatment of PMOP, as well as guiding future research.

## 2 Postmenopausal osteoporosis and the concept of bone immunology

### 2.1 Estrogen deficiency and imbalance in bone remodeling

Estrogen deficiency is a key upstream driver of PMOP, and its classic mechanism can be summarized as an imbalance in bone remodeling. This imbalance involves enhanced osteoclast activity while osteoblast function is suppressed, leading to long-term bone resorption exceeding bone formation, which results in decreased bone mass and destruction of bone microstructure (17, 18). In the classic model of the “osteocyte center,” the RANK/RANKL/OPG axis dominates the differentiation and activation intensity of osteoclasts, while the Wnt/β-catenin pathway determines osteogenic differentiation and bone formation capacity; both together maintain the homeostasis of bone remodeling (19). Estrogen can inhibit bone resorption and promote bone formation by lowering the RANKL/OPG ratio and maintaining Wnt/β-catenin activity. However, the postmenopausal decline of estrogen leads to an increase in the RANKL/OPG ratio and a weakening of Wnt/β-catenin signaling, thus forming the classic pathological picture of PMOP (20–23). It is noteworthy that the local signaling imbalance in osteocytes alone cannot fully explain the persistent bone loss in PMOP. Estrogen deficiency can also be accompanied by elevated pro-inflammatory cytokines and immune cell profile shifts, and these changes are coupled with the RANK/RANKL/OPG and Wnt/β-catenin pathways, further amplifying bone resorption signals and reinforcing the bias in bone remodeling (24). Additionally, abnormalities in immune-related pathways such as the NLRP3 inflammasome and ANXA1/FPR2 in bone remodeling-related cells also suggest that the bone metabolism process is deeply embedded in the immune regulation network (25). Therefore, in the subsequent chapters of this review, we emphasize the upgrade from this “classic osteocyte model” to the “bone immune model.” Under the background of estrogen deficiency, RANK/RANKL/OPG and Wnt/β-catenin are no longer just local signaling axes between osteocytes but are co-opted and reprogrammed by bone marrow immune cells, cytokine networks, and inflammation-aging signals. These changes thus drive the persistent bone loss in PMOP.

### 2.2 Bone marrow as a “niche” site for immunity and bone metabolism

Bone marrow is not only the site of generation and renewal of immune cells but also a key microenvironment where bone cells and immune cells coexist and regulate each other (26, 27). In this microenvironment, immune cells influence the processes of bone resorption and osteogenesis through cytokine networks and direct cell contact. The synergy of proinflammatory effectors and RANKL signaling is considered an important mechanistic basis driving enhanced bone resorption (28–31). Meanwhile, the metabolic state of immune cells and changes related to bone marrow fat can further shape the inflammatory phenotype and influence bone remodeling, forming a coupling network of “immune-metabolism-bone remodeling” (32–35). Therefore, examining PMOP from the perspective of the bone marrow microenvironment helps establish a more direct mechanistic bridge between “systemic inflammation/immune drift” and “local bone metabolic imbalance,” providing a conceptual basis for subsequent discussions on immune cell reprogramming, key signaling network elucidation, and immune-targeted intervention strategies (36).

In summary, bone loss in PMOP can be explained by the imbalance of the two major axes of RANK/RANKL/OPG and Wnt/β-catenin, and its persistence is likely amplified by inflammation-immune changes induced by estrogen deficiency and remodeling of the bone marrow microenvironment, providing a theoretical basis for precision interventions based on bone immunology.

### 2.3 The connotation and research significance of the bone immune microenvironment

**Figure 1:**
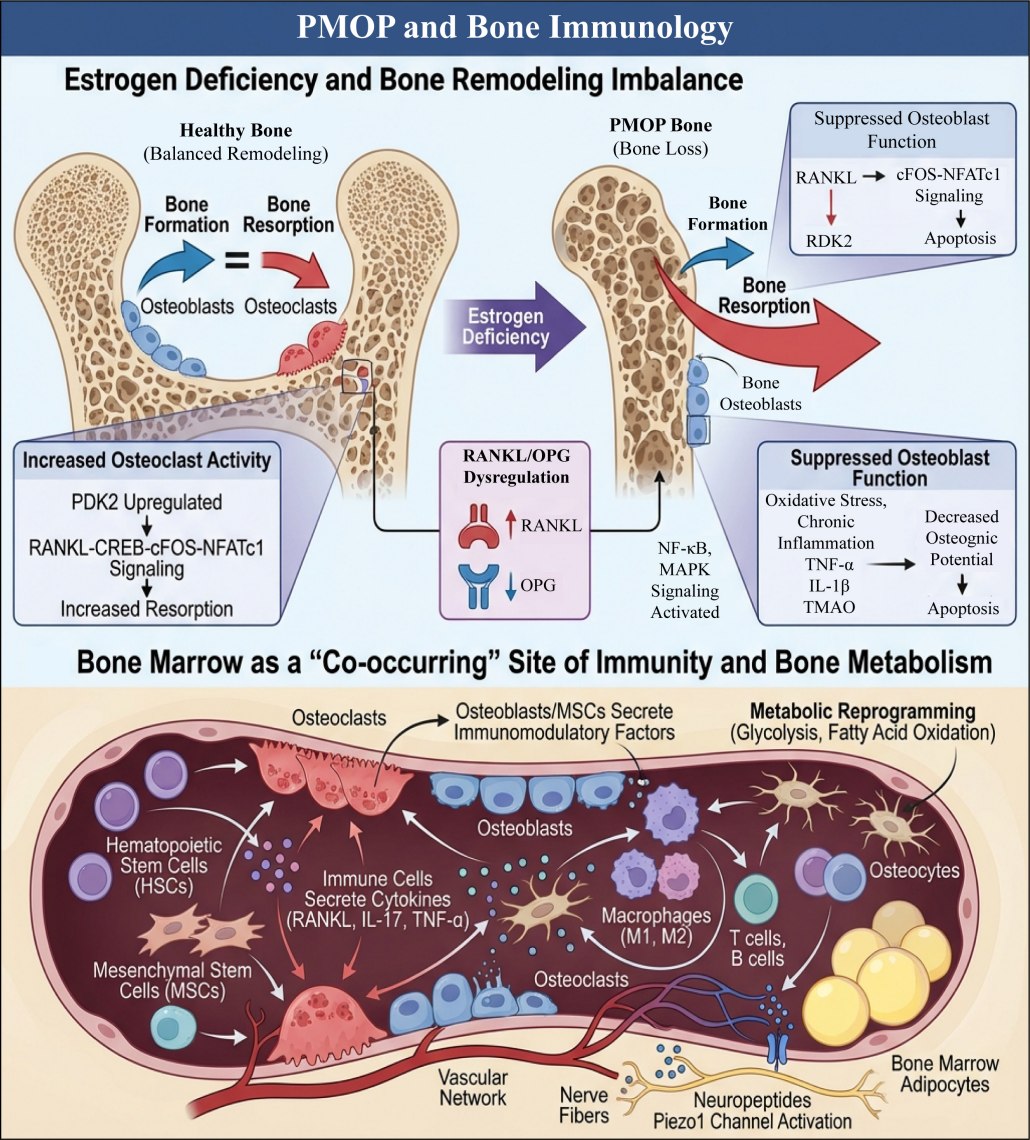
Schematic diagram of PMOP and bone immunology. Upper part: Postmenopausal estrogen deficiency disrupts the balance of bone remodeling. In healthy bones, the activity of osteoblasts and osteoclasts maintains a dynamic balance. After the decline of estrogen, the RANKL/OPG ratio becomes imbalanced, and the activation of PDK2 and RANKL-CREB-cFOS-NFATc1 signaling enhances osteoclast activity and increases bone resorption. Meanwhile, oxidative stress and chronic inflammation inhibit osteoblast function, reduce osteogenic potential, and induce apoptosis, ultimately leading to PMOP-related bone loss. Lower part: Bone marrow serves as an integrated site for immunity and bone metabolism. Hematopoietic stem/progenitor cells and MSCs differentiate into various immune cells and bone cells. T cells, B cells, macrophages, and other immune cells secrete cytokines such as RANKL, IL-17, and TNF-α to regulate osteoclast and osteoblast activity. Osteoblasts and MSCs release immune regulatory factors. Additionally, bone cells, bone marrow adipocytes, and metabolic reprogramming processes (including glycolysis and fatty acid oxidation) jointly participate in remodeling the bone immune microenvironment.

The bone immune microenvironment refers to the dynamic regulatory network formed by immune cells and stromal cells within the bone marrow, through the interaction of cytokines and receptor-ligand interactions. It serves as a key hub connecting immune homeostasis and the balance of bone remodeling (37–39). In PMOP, hormonal changes not only affect osteoblasts and osteoclasts themselves but also accompany the reprogramming of bone marrow immune cell functions and enhanced inflammatory states. These changes promote bone resorption, inhibit bone formation, and accelerate bone loss (40–42). In addition, BMSCs play a dual role in bone regeneration and immune regulation, and their phenotypic changes may further affect bone immune homeostasis, providing a basis for potential intervention targets (43). From a therapeutic perspective, current clinical interventions mainly focus on inhibiting bone resorption or promoting bone formation, such as anti-RANKL antibodies and bisphosphonates. However, incorporating immune cells and their inflammatory networks into the framework of bone metabolism regulation can provide new entry points for combined strategies aimed at immune regulation and restoration of bone remodeling balance. These strategies include regulating macrophage polarization; inhibiting key inflammatory factors and inflammasome pathways; and promoting the formation of an immunotolerant microenvironment (44, 45). Furthermore, the concept of dynamic regulation of the bone immune microenvironment provides theoretical support for bone regeneration materials and bone defect repair, that is, promoting bone healing and integration by optimizing immune responses (46, 47). In summary, research on the bone immune microenvironment not only deepens the understanding of the immunopathological mechanisms of PMOP but also offers a more translational theoretical framework for immune-targeted therapies and bone tissue engineering strategies (**Figure 1**).

## 3 Structural and compositional changes of the bone marrow microenvironment after menopause

This section mainly outlines the changes in the bone marrow microenvironment in PMOP from the perspective of “structure and niche,” focusing on identifying which cells and tissue components undergo reconstruction and their spatial distribution patterns. It emphasizes changes in bone marrow structure and cellular lineage composition, whereas the consequent immune lineage reprogramming and cytokine amplification effects are discussed in detail in Sections 4 and 5 to avoid redundant discussions of the same mechanisms across different levels.

### 3.1 Aging and phenotypic remodeling of hematopoietic stem/progenitor cells and MSCs

The occurrence of PMOP is accompanied by significant changes in the structure and cellular lineage composition of the bone marrow microenvironment, which involves aging and phenotypic remodeling of HSCs/HSPCs and MSCs, a fundamental process that drives the imbalance of the bone immune microenvironment (7). First, hematopoietic stem cells exhibit typical functional decline and lineage bias after menopause. In aging and menopause-related models, the total number of bone marrow HSCs does not necessarily decrease significantly, but their self-renewal capacity and long-term hematopoietic potential weaken, showing a tendency to differentiate toward the myeloid lineage, while lymphoid generation is relatively suppressed, leading to an imbalance in immune cell generation (48). This “myeloid skewing” is specifically manifested as an increase in the proportion of myeloid cells such as monocytes/macrophages and neutrophils, while the generation of lymphocytes such as B cells and T cells decreases. This shift not only weakens adaptive immune responses but also lays the cellular foundation for the formation of a pro-inflammatory environment in the bone marrow.

MSCs, as key supporting cells for HSCs and precursors of osteoblasts, also profoundly affect the bone marrow microenvironment through their aging and phenotypic remodeling (49). In postmenopausal and aging bone marrow, the proliferation capacity of MSCs decreases, their colony-forming ability weakens, and their osteogenic differentiation potential significantly declines, while the tendency to differentiate into adipocytes increases, leading to the accumulation of bone marrow adipose tissue (BMAT) and a reduction in bone mass (50). During this process, the expression of key receptors such as CXCR4 in aged MSCs is downregulated, weakening their supportive capacity for HSPCs and disrupting the homeostasis maintenance of the “MSC–HSC” axis. Co-culture experiments show that co-culturing aged MSCs with young HSPCs can induce premature aging and myeloid skewing phenotypes in the latter, suggesting that MSC dysfunction directly reshapes HSC fate determination through intercellular signaling (51).

At the transcriptional and signaling pathway levels, multiple pathways regulating lineage differentiation undergo reprogramming in aged MSCs. For example, the transcription factor EBF1 plays a key role in maintaining the osteogenic capacity and immune homeostasis of MSCs, and its absence can lead to impaired MSCs proliferation, decreased osteogenic capacity, and upregulation of inflammation-related gene expression in the bone marrow microenvironment, thereby promoting myeloid skewing and hematopoietic abnormalities in HSCs (52). In addition, the secretion profile of aged MSCs also changes, regulating the cell cycle and colony-forming ability of HSPCs through the secretion of cytokines and extracellular vesicles (EVs), with changes in the composition of miRNA within EVs being considered one of the important mechanisms mediating the functional imbalance of the MSCs–HSPCs axis (53). In the context of PMOP, the disruption of the osteogenic–adipogenic differentiation balance of MSCs not only directly leads to insufficient bone formation but also indirectly affects the spatial distribution and activation state of bone marrow immune cells by promoting BMAT expansion. The adipokines and inflammatory mediators secreted by BMAT can further alter the lineage composition and activation thresholds of bone marrow immune cells, making “MSCs aging–lineage skewing–BMAT increase” a structural hub connecting abnormal bone metabolism and immune reprogramming (54).

In summary, the functional decline and myeloid skewing of bone marrow HSCs after menopause, along with the aging of MSCs, decreased osteogenic potential, and reprogramming towards the adipocyte lineage, collectively reshape the cellular lineage composition and spatial structure of the bone marrow. This provides a “platform” for subsequent immune reprogramming, chronic inflammation maintenance, and bone loss. It should be noted that the aging-related secretory phenotype (SASP) and chronic low-grade inflammation (inflammaging) mechanisms derived from the aforementioned changes will be systematically elaborated in sections 5.4 and 6.2 from the perspectives of “local bone marrow aging” and “systemic inflammatory aging,” respectively. It should be emphasized that most evidence regarding the mechanisms related to HSC/HSPC aging and their association with BMAT expansion mainly comes from aging/castrated animal models and studies related to bone marrow aging. Furthermore, there is still a lack of systematic human data on the fine structure and functional changes of the bone marrow niche in typical PMOP patients. Therefore, the pathological framework of “MSC aging–lineage skewing–BMAT increase” outlined in this section is more of an analogical integration based on related osteoporosis and aging research, and its specific mechanisms in the PMOP population require further validation. The specific molecular and signaling interactions with the BMAT–immune–bone axis will be further elaborated in section 3.3.

### 3.2 Osteoblasts, osteoclasts, and the reconstruction of the osteocyte network

Under normal physiological conditions, bone remodeling alternates between the bone resorption phase mediated by osteoclasts and the bone formation phase dominated by osteoblasts. Osteoclasts dissolve minerals and degrade the organic bone matrix by secreting acidic substances and proteases, while releasing growth factors stored in the bone matrix. Subsequently, these signals can recruit and activate osteoblasts to complete the refilling and reconstruction of bone, thus forming a tightly coupled “resorption–formation” cycle (55, 56). In this process, osteoblasts originate from BMSCs, whose differentiation and function are finely regulated by various signaling pathways and transcription factors to ensure the continuous repair of local micro-damage and the long-term maintenance of bone microstructure (57, 58).

Osteocytes, as the most numerous and longest-lived cells in bone tissue, are embedded in the lacunae and canaliculi of the bone matrix, and interconnected by their dendritic processes to form a dense “osteocyte network” (LCN), which is the core mechanosensor and signaling hub of bone tissue (59). Osteocytes can sense mechanical stress and metabolic changes, communicating with neighboring osteocytes, osteoblasts, and osteoclast precursors on the bone surface through gap junctions and EVs (60). The OPG, RANKL, and various factors they produce can directly regulate osteoclast differentiation and activity, and provide feedback to modulate the differentiation and mineralization capacity of osteoblasts, thus coordinating bone resorption and bone formation in both spatial and temporal dimensions (61). In a healthy bone environment, osteocytes, osteoblasts, and osteoclasts form a dynamically balanced “osteocyte–bone remodeling coupling unit.”

In the context of PMOP, the combined effects of estrogen deficiency and chronic inflammation in the bone marrow microenvironment disrupt this coupling systemically. On the one hand, the differentiation and mineralization capacity of osteoblasts decline, with the upstream MSC osteogenic potential weakening and shifting towards the adipogenic lineage, leading to insufficient new bone formation. On the other hand, both the number and function of osteoclasts are enhanced, with bone resorption continuously dominating, presenting a typical “bone resorption > bone formation” imbalance (62). From the perspective of signaling pathways, estrogen deficiency and pro-inflammatory cytokines can inhibit the expression of osteogenic-related genes by activating pathways such as NF-κB and STAT3, weakening the synthesis of bone matrix proteins (such as osteocalcin and osteopontin) and bone morphogenetic proteins, further amplifying osteogenic defects (63, 64). In this context, some natural products or Chinese medicine compounds (such as deer antler extract) with both immune and metabolic regulatory effects can inhibit osteoclasts and promote osteoblasts, providing preliminary pharmacological evidence for integrating bone immune regulation (65–67).

**Figure 2:**
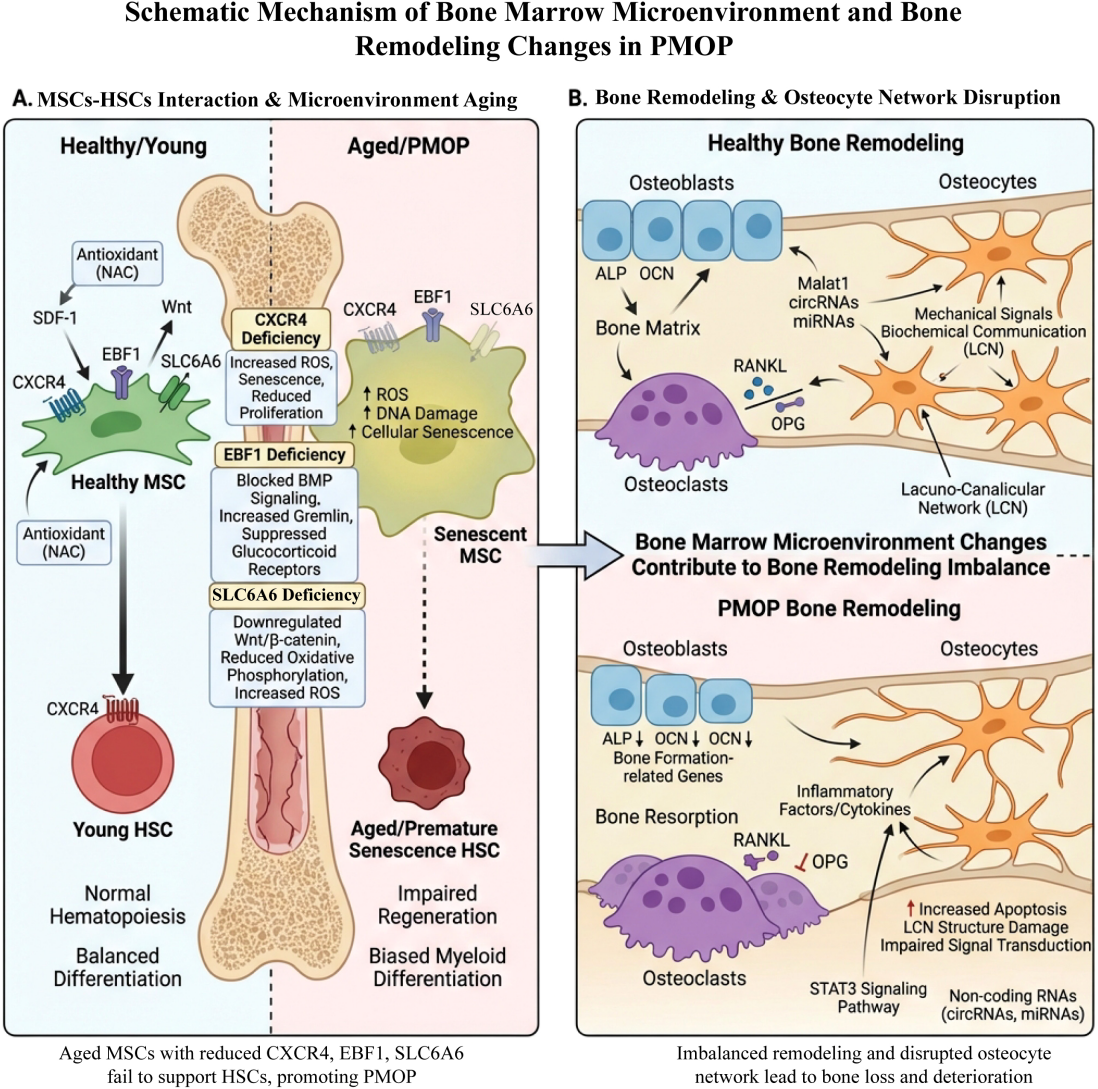
Schematic diagram of changes in bone remodeling in the bone marrow microenvironment and PMOP. **(A)** In a healthy/young state, MSCs maintain antioxidant capacity and microenvironment homeostasis through pathways such as CXCR4, EBF1, and SLC6A6, thereby supporting normal hematopoiesis and balanced differentiation of HSCs. In the postmenopausal/aged PMOP state, MSCs exhibit elevated ROS, DNA damage, and cellular senescence, along with downregulation of CXCR4, EBF1, and SLC6A6 expression, impaired BMP signaling, and disrupted oxidative phosphorylation. These changes lead to decreased regenerative capacity of HSCs and a shift toward a myeloid aging phenotype characterized by altered myeloid lineage differentiation. **(B)** Abnormalities in the bone marrow microenvironment further affect bone remodeling. In healthy bone, osteoblasts, osteoclasts, and osteocytes maintain the balance of bone formation and bone resorption through networks involving RANKL/OPG, mechanical signals, and CircRNA/miRNA. In PMOP, osteoblast-related markers (such as ALP, OCN) are downregulated, RANKL/OPG imbalance occurs, and inflammatory factors are upregulated, accompanied by increased apoptosis of bone cells and disruption of the LCN structure, ultimately leading to imbalanced bone remodeling and bone loss.

At the same time, the osteocyte network itself also undergoes structural damage in PMOP: increased osteocyte apoptosis, destruction of the LCN structure, and weakened mechanosensing and signal transmission capabilities, leading to impaired adaptive remodeling of bone tissue to mechanical stimuli and metabolic signals (68). The EVs secreted by osteocytes play an important role in regulating communication between osteoblasts, osteoclasts, and immune cells, and their dysfunction further weakens local bone repair capacity, amplifying the osteoclast/osteoblast imbalance (69–71). Overall, in PMOP, the combination of “decreased osteoblast number and function + increased osteoclast activity + structural damage to the osteocyte network and weakened mechanosensing” constitutes the cellular basis for the deterioration of bone microstructure and bone mass loss (72, 73). (**Figure 2**). It should be noted that many detailed mechanisms regarding the imbalance of osteoblast/osteoclast coupling and the destruction of the osteocyte network are still mainly based on castrated animal models, bone defect repair models, and some other basic research on bone metabolic diseases (such as RA-related bone destruction), and the high-resolution structural and functional verification of human PMOP bone specimens is relatively limited. Therefore, the description of the imbalance of “osteoblasts–osteoclasts–osteocytes” in this section can be seen more as a pathological framework extrapolated from related models to PMOP, and the specific degree and population differences still need to be clarified by further clinical and translational research.

In summary, this section primarily outlines the morphological and cellular basis of the imbalance in osteoblast-osteoclast coupling in PMOP with respect to “bone structure and osteocyte network.” Specific cytokine networks, RANKL signaling activation, and their cascading effects with immune cells will be elaborated in subsequent sections 4.3 and 5.1, and will not be discussed here again.

### 3.3 Increased bone marrow adipose tissue: The relationship among fat, immunity, and bone

BMAT plays an important role in the bone marrow microenvironment, with its quantity and function undergoing dynamic changes under various physiological and pathological conditions (74). BMAT is not only an energy-storing fat depot but also an active tissue involved in endocrine and immune regulation, participating in the modulation of bone metabolism and immune response. Particularly in diseases associated with reduced bone mass, such as PMOP, the increase in bone marrow adipocytes is considered one of the important factors for bone loss (75). This section focuses on the mechanisms by which the increase in bone marrow adipocytes regulates immune response and bone metabolism through the secretion of adipokines. It also examines how the interaction between adipose tissue and immune cells promotes inflammatory states and inhibits the osteogenic process. Firstly, the increase in bone marrow adipocytes is accompanied by changes in the secretion of adipokines (such as adiponectin, leptin, inflammatory cytokines, etc.), which regulate the metabolic balance of bones through paracrine and endocrine pathways (76). Studies have shown that the expansion of bone marrow adipose tissue is closely related to the decline in bone mass. Pro-inflammatory factors produced by adipocytes can activate immune cells in the bone marrow, inducing local chronic low-grade inflammation, thereby promoting bone resorption and inhibiting bone formation (77). For example, leptin secreted by bone marrow adipocytes not only regulates systemic energy metabolism but also exerts signaling effects through its receptors on bone marrow immune cells and osteocytes, affecting the inflammatory state of the bone marrow microenvironment and the dynamics of bone metabolism (78).

Secondly, there exists a complex interaction between adipose tissue and immune cells. Bone marrow adipocytes secrete various pro-inflammatory cytokines and chemokines, attracting and activating macrophages, T cells, and other immune cells in the bone marrow, which prompts their polarization toward pro-inflammatory phenotypes, thus forming a chronic inflammatory microenvironment (79). This inflammatory state not only inhibits the differentiation and function of osteoblasts but also activates osteoclasts, increasing bone resorption activity, leading to the occurrence and progression of osteoporosis (80). Moreover, fatty acids and related proteins (such as FABP4) in adipose tissue participate in lipid metabolism and inflammatory signal transduction, further exacerbating the immune imbalance in the bone marrow microenvironment (81). Moreover, the metabolic state of bone marrow adipocytes and their interactions with immune cells are regulated by endocrine factors and external environments. For instance, postmenopausal estrogen deficiency promotes the accumulation of bone marrow adipose tissue and activates immune cells through inflammatory factors secreted by adipocytes, forming a pro-inflammatory microenvironment that leads to bone loss (82). Certain receptors on adipocytes, such as the activation or inhibition of the CD40 signaling pathway, can also regulate the function of bone marrow immune cells, affecting bone metabolism and the state of adipocytes (83). Additionally, bone marrow adipose tissue interacts with BMSCs and other bone marrow stromal cells to regulate their differentiation towards adipocytes or osteoblasts, thereby influencing bone repair and remodeling (84).

**Figure 3:**
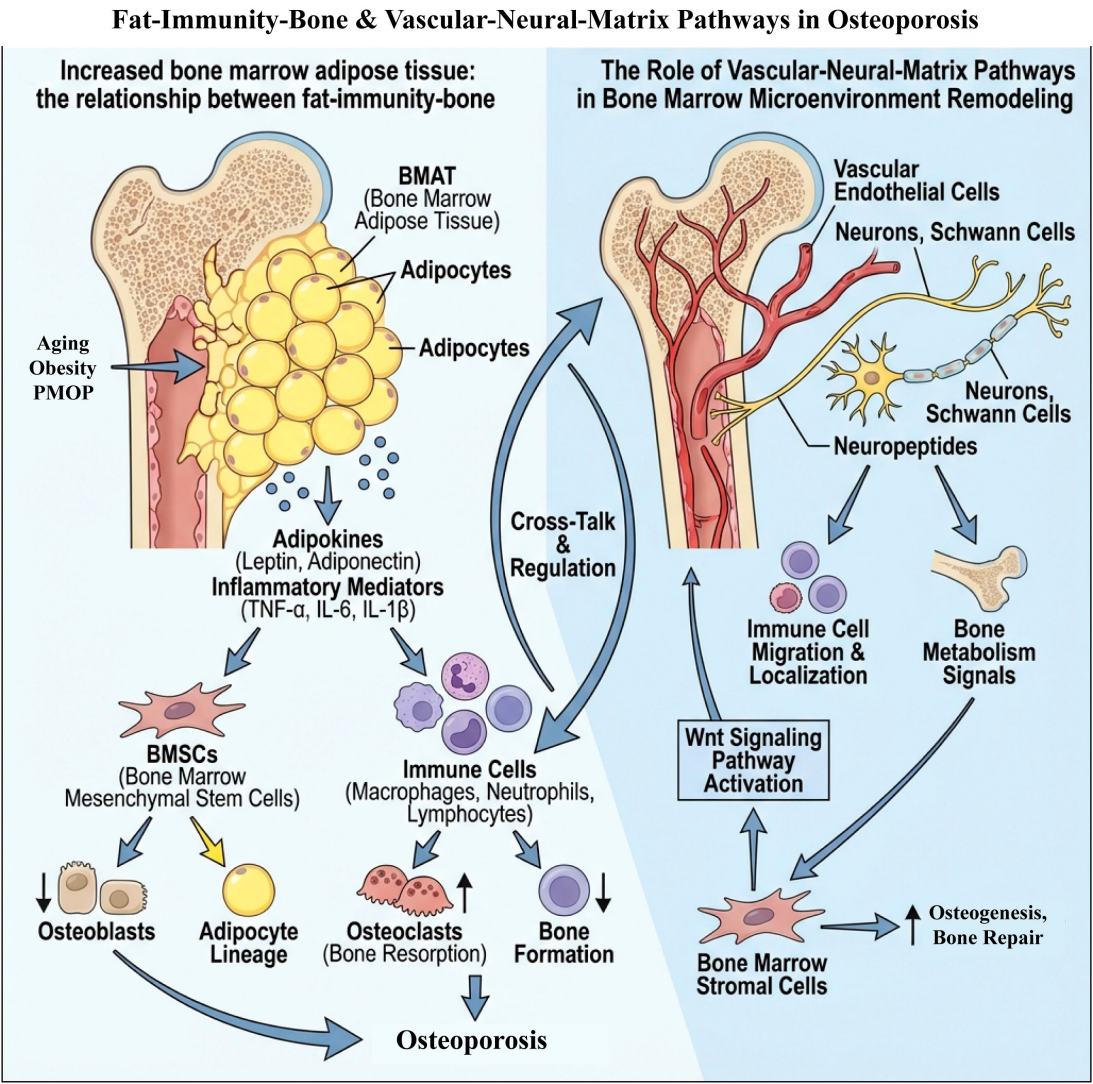
Schematic diagram of the role of the adipose–immune–bone axis and the vascular–nerve–matrix pathway in osteoporosis. Left side: Aging, obesity, and PMOP increase BMAT. Adipocytes secrete adipokines (such as leptin, adiponectin), and inflammatory mediators (TNF-α, IL-6, IL-1β), which alter the differentiation of BMSCs, inhibit osteoblast differentiation, and promote adipocyte activity and osteoclast activation. These changes lead to decreased bone formation, increased bone resorption, and progression to osteoporosis. Right side: Endothelial cells, neurons, and Schwann cells in the bone marrow microenvironment regulate the migration, localization, and function of immune cells through neuropeptides, Wnt signaling, and bone metabolism signals, thereby promoting or inhibiting osteogenesis and bone repair. The adipose–immune–bone axis and the vascular–nerve–matrix pathway achieve cross-talk and regulation through immune cells, jointly driving the imbalance of bone metabolism.

In summary, the increase in bone marrow adipose tissue is not only a phenotypic marker of bone metabolic diseases such as osteoporosis but also regulates the activity and inflammatory state of immune cells in the bone marrow through the secretion of various adipokines, forming a complex regulatory network of fat–immune–bone. The interaction between adipose tissue and immune cells promotes the inflammatory state of the bone marrow microenvironment, inhibits osteoblast activity, enhances bone resorption, ultimately leading to bone loss. In animal models related to PMOP and clinical observations, the expansion of BMAT is highly correlated with bone loss and fracture risk, suggesting that bone marrow fat is not only a passive marker of bone marrow aging but also an important “amplifier” of immune reprogramming and inflammatory bone loss. A deeper understanding of the mechanisms by which bone marrow adipose tissue functions in the bone immune microenvironment is expected to provide new therapeutic strategies and intervention targets for diseases such as PMOP (**Figure 3**). This subsection focuses on the expansion of BMAT and its role as a structural and endocrine hub of “fat–immune–bone”; the specific immune lineage changes driven by adipokines and the remodeling of cytokine networks will be elaborated in Sections 4 and 5 from the perspectives of “immune phenotype” and “signaling pathways,” respectively.

### 3.4 The role of the vascular-nerve-matrix pathway in the remodeling of the bone marrow microenvironment

The remodeling of the bone marrow microenvironment constitutes a key link in the pathogenesis of osteoporosis, where the interactions among blood vessels, nerves, and matrix components form a complex regulatory network. The impairment of bone marrow angiogenesis plays a crucial role in the pathological process of osteoporosis. Under normal circumstances, bone marrow blood vessels not only provide essential nutrients and oxygen to osteocytes but also provide channels for the migration and distribution of immune cells (85). However, in obesity and aging models induced by a high-fat diet, dysfunction of bone marrow blood vessels leads to reduced angiogenesis in the bone marrow microenvironment, which in turn affects the directional migration and activity of immune cells, decreases immune defense efficiency, and weakens the metabolic support for osteocytes. This abnormal angiogenesis not only limits the rate of cellular renewal within the bone marrow but also promotes the accumulation of adipocytes in the bone marrow, creating a microenvironment conducive to bone resorption, ultimately leading to reduced bone mass and structural damage (86).

Secondly, nerve signals play an important role in bone immune regulation. The abundant nerve fibers within the bone marrow regulate the functional state of immune cells and bone metabolism processes by releasing neurotransmitters and neuropeptides. The conduction of nerve signals regulates the activation and secretion functions of immune cells such as bone marrow macrophages and T cells, influencing the balance of bone remodeling (36). In the pathological state of osteoporosis, the dysregulation of the nerve-immune axis can exacerbate the inflammatory response in the bone marrow, enhance the activity of bone resorption cells, and inhibit the function of osteoblasts (87). Furthermore, nerve signals may mediate the interaction between the bone marrow and the central nervous system, forming a systemic regulatory network that modulates the dynamic changes in the bone immune microenvironment (88).

Additionally, the ECM of the bone marrow serves as an important “scaffold” for maintaining the structure and signal transduction of the bone marrow, and changes in its components and physical properties profoundly affect cell behavior (89). After menopause, the imbalance of ECM components such as type I collagen and proteoglycans, as well as changes in matrix stiffness, can directly regulate the adhesion, migration, and colonization of HSCs/HSPCs and MSCs, thereby influencing their differentiation tendencies between osteogenic/adipogenic lineages. At the same time, ECM remodeling alters the spatial presentation of adhesion molecules and chemokines, reshaping the distribution patterns of immune cells such as T cells and macrophages within the bone marrow cavity. Increasing evidence suggests that abnormal ECM remodeling after menopause is closely related to immune cell activation and inflammation amplification, maintaining a low-grade chronic inflammatory state in the bone marrow by promoting the sustained release of pro-inflammatory factors, thereby further exacerbating the bone loss process (90). However, current direct evidence regarding the abnormal vascular-nerve-matrix pathway in PMOP mainly comes from studies on bone biopsies of castrated/aged animal models, obesity-related models, and some other bone disease patients, lacking systematic human data on the fine structure and mechanical characteristics of the bone marrow niche in typical PMOP patients. Therefore, this section provides more of a “pathological framework based on related models” rather than definitive conclusions fully validated in the PMOP population.

In summary, the vascular-nerve-matrix pathway collaborates in the remodeling of the bone marrow microenvironment: impaired angiogenesis limits the migration of immune cells and the nutritional supply to osteocytes; meanwhile, the abnormal remodeling of ECM components and matrix stiffness alters the adhesion, migration, and spatial distribution of HSCs/MSCs and maintains chronic inflammation; additionally, nerve signals participate in the pathological process of osteoporosis by regulating immune cell functions. This section focuses on the remodeling at the “structural and niche” level, providing space and contextual background for the subsequent discussions on immune lineage changes (Section 4) and chemotactic axes (Section 5.3), avoiding repetitive mechanistic elaboration on the same group of cells and factors at different levels. A deeper understanding of these mechanisms provides new perspectives for the study of the bone immune microenvironment in PMOP and lays the foundation for the development of future therapeutic strategies targeting the bone marrow microenvironment (**Figure 3**).

## 4 Immune reprogramming in PMOP

After establishing the “bone marrow structure and ecological niche/lineage chassis” in Section 3, this section shifts to the immune level. It concentrates on the reprogramming of innate and adaptive immune cell lineages and phenotypes, addressing “which immune cells have experienced functional shifts and in which directions.” The specific remodeling of the cytokine network is summarized in Section 4.3 in the form of a “mediator layer,” while the signal integration and effects on the osteocyte side are further explored at the pathway level in Section 5, with the aim of presenting a hierarchical, rather than parallel, analysis.

### 4.1 Reprogramming of innate immune cells

The functions and phenotypes of innate immune cells in the bone marrow microenvironment are regulated by various internal and external environmental factors, especially under the pathological state of PMOP, where their immune reprogramming exhibits significant characteristics. Bone marrow macrophages polarize towards the pro-inflammatory M1 type, becoming a key factor in the progression of osteoporosis (91). In this process, the output of inflammatory factors from M1 macrophages is enhanced (such as TNF-α, IL-1β, and IL-6). To avoid elaborating on the detailed mechanisms of the cytokine network in different sections, this section only emphasizes the phenotypic characteristics of “pro-inflammatory polarization + enhanced osteoclast activity.” The specific positions and synergistic effects in the cytokine network will be integrated and elaborated in section 4.3. M1 macrophages enhance osteoclast activity, speeding up the absorption of bone matrix and bone loss (92). This polarization shift is driven not only by the enhancement of inflammatory signals in the bone marrow microenvironment but also closely related to the remodeling of HSCs/HSPCs functions.

The recently proposed concept of “trained immunity” provides a new theoretical framework for understanding the reprogramming of innate immunity in the bone marrow. Brief microbial or inflammatory stimuli (such as BCG or β-glucan) can induce HSPCs and their progeny myeloid cells to acquire a long-term “memory-like” enhanced response capability through metabolic and epigenetic remodeling, resulting in a stronger inflammatory response upon re-encountering stimuli (93, 94). This mechanism has protective significance in anti-infection and anti-tumor contexts, but in chronic bone metabolic diseases, it may manifest as the maintenance of persistent low-grade inflammation. In the context of PMOP, factors such as estrogen deficiency, bone marrow aging, and BMAT expansion overlap, making “trained immunity” more likely to solidify towards a pro-inflammatory phenotype, forming what is known as “trained inflammatory marrow,” providing a long-term drive for inflammatory bone loss (95). This part mainly supplements the “immune consequences” of HSC myeloid bias described in section 3.1 at the level of immune function and memory-like reprogramming, without repeating the structural details of cell lineages and ecological niches.

Monocytes and dendritic cells (DCs) also undergo significant phenotypic and functional remodeling, participating in the formation and maintenance of the inflammatory environment. Studies have found that inflammatory and metabolic reprogramming can induce monocytes to transition to a pro-inflammatory phenotype, with the cytokines and chemokines they secrete further exacerbating the inflammatory microenvironment in the bone marrow (96). As a bridge connecting innate and adaptive immunity, the functions of DCs are regulated by the skeletal metabolic environment, which may manifest as a decline in antigen presentation capacity and abnormal release of inflammatory signals in osteoporosis, leading to impaired immune surveillance and imbalanced bone remodeling (97). Additionally, metabolites in the bone marrow microenvironment, such as lactate, can also regulate the signaling pathways of macrophages and dendritic cells, affecting their polarization states and functional activities, further promoting pro-inflammatory responses (98). This reprogramming of innate immune cells is not limited to the local bone marrow but interacts with systemic inflammatory responses and the bone-immune axis. Recent studies have shown that hematopoietic stem cells in the bone marrow undergo metabolic and epigenetic reprogramming under external stimuli, forming a trained immunity state, which is beneficial for anti-infection and anti-tumor responses but may also become the pathological basis for chronic inflammation and diseases such as osteoporosis (99, 100). In PMOP, this trained immunity is more inclined to maintain a low but persistent pro-inflammatory environment, keeping osteoclast signaling in a mildly activated state, becoming a “hard-to-turn-off engine” for bone loss (101).

In summary, the reprogramming of bone marrow innate immune cells in PMOP is characterized by the polarization shift of macrophages towards the pro-inflammatory M1 type, enhancing osteoclast activity; at the same time, the phenotypic and functional changes of monocytes and dendritic cells collectively shape a stable pro-inflammatory bone immune microenvironment. These changes are maintained through complex metabolic and epigenetic mechanisms and are highly consistent with the concept of trained immunity. Future research should focus on precisely regulating the states of these immune cells, especially in the context of estrogen deficiency and bone marrow aging, in order to restore bone marrow immune homeostasis and thus slow down the progression of osteoporosis.

### 4.2 Adaptive immune cell lineage changes

Adaptive immune cells play a key role in the bone marrow microenvironment, and changes in their lineage and function are closely related to osteoporosis, especially PMOP. The decline in estrogen levels after menopause leads to complex reprogramming of the immune system, affecting the homeostasis of the bone immune microenvironment and the balance of bone metabolism (102). Firstly, the imbalance of Th17/Treg cells is a core link in the changes of adaptive immune cell lineages. Th17 cells are a type of helper T cell that promotes inflammatory responses, and the IL-17 they secrete can significantly promote the differentiation and activity of osteoclasts, thereby enhancing bone resorption and leading to a decrease in bone mass. In contrast, Treg primarily suppress immune responses, maintain immune tolerance, and protect bone tissue from excessive destruction (103). After menopause, due to estrogen deficiency, the suppressive function of Treg cells weakens, while the number and activity of Th17 cells increase, leading to an imbalance between the two, resulting in a pro-resorptive inflammatory environment that facilitates the onset and progression of osteoporosis (104). Relevant studies suggest that regulating the Th17/Treg balance is a potential therapeutic strategy to alleviate PMOP (105). Secondly, the activation of T follicular helper cells (Tfh) and CD8^+^ T cells also participates in bone immune regulation and the process of inflammatory aging. Tfh cells mainly support the maturation of B cells and antibody production. Their activation affects the composition and function of B cell subsets in the bone marrow microenvironment, thereby indirectly regulating bone metabolism (106, 107). At the same time, CD8^+^ T cells, as a type of cytotoxic T lymphocyte, participate not only in immune surveillance within the bone marrow but also influence the function of osteoclasts and osteoblasts by secreting cytokines. With aging and menopause, the functional state of CD8^+^ T cells changes, exhibiting characteristics of inflammatory aging, which may exacerbate the pathological process of osteoporosis (108).

Moreover, changes in B cell subsets are also crucial in the bone immune environment. Regulatory B cells (Breg) have immunosuppressive effects and can secrete anti-inflammatory cytokines such as IL-10 to inhibit excessive immune activation and maintain immune balance in the bone marrow (109). After menopause, the number of Breg cells decreases, and their immunosuppressive ability weakens, making pro-inflammatory T cells and osteoclast-related signals more dominant (110). In contrast, the number of age-related B cells (ABCs) significantly increases. These B cells exhibit a strong pro-inflammatory phenotype and significantly upregulate RANKL expression, which can directly promote osteoclast differentiation and bone resorption (111, 112). Therefore, the transition of B cell subsets from “Breg dominance” to “ABCs dominance” constitutes a typical adverse reprogramming of the adaptive immune system to the skeletal environment (113). However, current associations between Th17/Treg imbalance, Breg/ABCs reconstruction, and PMOP are mainly based on small-scale clinical studies of peripheral blood/limited bone marrow samples from postmenopausal osteoporosis patients; some mechanistic understandings of Tfh, CD8^+^ T cells, and ABCs are more derived from other chronic immune diseases such as rheumatoid arthritis. Therefore, the inferences in this section under the context of PMOP still have certain analogical and exploratory nature (105, 114).

In summary, in the bone marrow immune microenvironment of PMOP, significant changes occur in the lineage of adaptive immune cells. The imbalance of Th17/Treg cells promotes inflammation and bone resorption; the activation of Tfh and CD8^+^ T cells participates in bone immune regulation and inflammatory aging; and the reconstruction of B cell subsets regulates bone metabolism through RANKL. More importantly, the changes in the aforementioned T/B cell axis synergize with RANKL upregulation and NLRP3 inflammasome activation, forming a key node of inflammatory bone loss in the PMOP bone immune microenvironment, laying an immunological foundation for the subsequent discussion on cytokine networks and signaling pathways.

### 4.3 Cytokine network remodeling: The “mediating layer” connecting immune lineage and osteocyte effects

The remodeling of the cytokine network in the bone marrow immune microenvironment is an important aspect of the pathogenesis of PMOP. After menopause, due to the decline in estrogen levels, the activity of immune cells in the bone marrow increases, and the expression of pro-inflammatory cytokines is significantly upregulated, resulting in increased osteoclast activity, aggravated bone resorption, and significant bone loss (115). In this process, the increased ratio between RANKL and its inhibitor OPG is a direct manifestation of enhanced bone resorption signaling (116, 117). Centered on this “core of bone resorption signaling,” various pro-inflammatory cytokines such as TNF-α, IL-6, IL-17, and IFN-γ form a synergistic amplification network (118). Considering that these factors have been elaborated from the “cellular origin” perspective in sections 4.1 (M1 polarization) and 4.2 (Th17/Treg imbalance, B cell subset reconstruction), this section will not repeat the discussion by cell type, but rather emphasize their common effects: Immune lineage reprogramming is “translated” into a persistent osteoclastic signal perceivable by the skeletal system through upregulating RANKL, downregulating OPG, and directly or indirectly enhancing osteoclast differentiation and survival (119, 120).

In addition, the activation of inflammasomes such as NLRP3 is closely related to inflammatory bone loss. The NLRP3 inflammasome senses intracellular danger signals, activating the maturation and release of IL-1β and IL-18, promoting local inflammatory responses (121). Studies have shown that the NLRP3 inflammasome is activated in osteoporosis models, promoting the formation of a bone marrow inflammatory environment, exacerbating osteoclast-mediated bone resorption, and synchronously upregulating the expression of pro-inflammatory factors such as TNF-α and IL-6 (122). From a hierarchical perspective, the NLRP3–IL-1β/IL-18 axis can be viewed as an “amplifier”: on one hand, it deepens the pro-inflammatory phenotype of immune cells, and on the other hand, it strengthens the coupling of the cytokine cluster with the RANKL axis, providing upstream driving forces for pathway-level imbalance.

**Figure 4:**
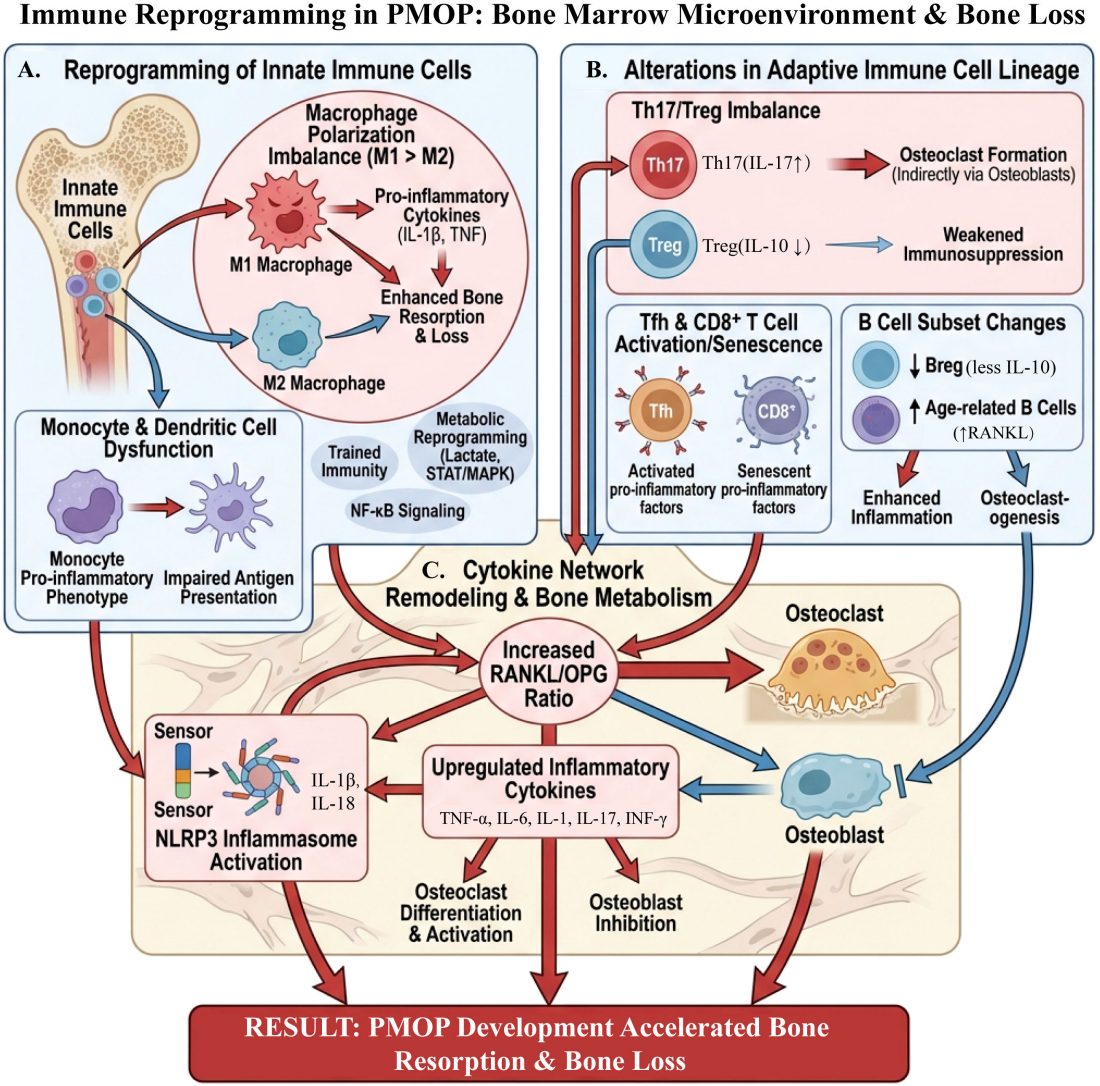
Schematic diagram of immune reprogramming and bone marrow microenvironment imbalance in PMOP. Part A: Reprogramming of innate immune cells. In postmenopausal osteoporosis, macrophage polarization is imbalanced (M1↑, M2↓), which enhances bone resorption through pro-inflammatory factors such as IL-1β and TNF, as well as through metabolic and signaling changes involving lactate and the STAT/MAPK and NF-κB pathways. Monocytes and dendritic cells exhibit a sustained pro-inflammatory phenotype and impaired antigen presentation, further amplifying local inflammation in the bone marrow. Part B: Changes in adaptive immune lineages. An imbalance of Th17/Treg (Th17↑, Treg↓) promotes osteoclast formation and weakens immune suppression. Additionally, Tfh and CD8^+^ T cells secrete large amounts of pro-inflammatory factors after activation or senescence. B cell subpopulations transition from immunoregulatory Breg cells to age-related B cells associated with RANKL, enhancing inflammation and osteoclastogenesis. Part C: Remodeling of cytokine networks and imbalance in bone metabolism. The NLRP3 inflammasome is activated, leading to upregulation of pro-inflammatory cytokines including TNF-α, IL-6, IL-1, IL-17, and IFN-γ. This is accompanied by an increased RANKL/OPG ratio, which promotes osteoclast differentiation and activation while suppressing osteoblast function. The final result is accelerated bone resorption and progressive loss of bone mass in PMOP.

In summary, the bone immune microenvironment of PMOP is characterized by an “immune-bone interface” mediating layer, centered on the imbalance of RANKL/OPG and represented by a cluster of pro-inflammatory factors such as TNF-α, IL-6, IL-17, IFN-γ, and NLRP3–IL-1β/IL-18. This mediating layer connects the structural remodeling and immune lineage changes described in sections 3–4 above, and below, it coordinates the terminal effects of osteogenesis/resorption through RANK and related signaling pathways, thus completing the logical closed loop of “immune reprogramming → bone metabolism imbalance” without repeating the cellular and factor backgrounds (**Figure 4**).

## 5 Key signaling pathways and molecular networks of bone immune microenvironment

After focusing on structure and niche in Section 3 and clarifying the immune lineage and cytokine “intermediate layer” in Section 4, this section further delves into signaling pathways and temporal dimensions. It focuses on RANK/RANKL/OPG and Wnt as “osteocyte-side signaling hubs,” CXCL12–CXCR4/S1P as “spatial positioning axes,” and bone marrow aging and SASP as chronic driving forces across cellular, tissue, and systemic levels. The section also explains how they converge to ultimately cause osteoclast-dominated bone metabolic imbalance.

### 5.1 RANK/RANKL/OPG axis: The “osteocyte hub” that translates immune signals into osteoclast effects

The RANK/RANKL/OPG axis is a key regulatory system for maintaining skeletal homeostasis, which plays a central role in the pathological process of PMOP. RANKL is secreted by various cells in the bone marrow microenvironment (including osteoblasts, osteocytes, B cells, and some T cells), and can bind to its receptor RANK, promoting the differentiation and activation of osteoclasts, thereby enhancing bone resorption function (123, 124). In contrast, OPG, as a soluble RANKL “decoy” receptor, can competitively bind to RANKL, blocking its interaction with RANK. This inhibits the generation and activity of osteoclasts, maintaining the balance of bone remodeling (125, 126). Under normal circumstances, the dynamic balance between RANKL and OPG ensures the coordination of bone resorption and bone formation, maintaining the homeostasis and health of bone tissue (127).

In postmenopausal women, this balance is disrupted due to a significant decrease in estrogen levels (128). As described in section 4.3, various pro-inflammatory cytokines (including TNF-α, IL-6, IL-17, etc.) promote an increase in the RANKL/OPG ratio by upregulating RANKL and downregulating OPG. To avoid repetitive explanations of the same group of factors and their general inflammatory effects in different sections, this section will not elaborate on their immunological background but will focus on the integration and amplification function of this axis on the “osteocyte side.” Once the RANKL/OPG ratio crosses a critical threshold, osteoclast precursors are continuously activated through RANK-mediated NF-κB, MAPK, and other pathways, promoting their differentiation into mature osteoclasts and extending their lifespan, resulting in a long-term imbalance of “bone resorption > bone formation” (129–131). Furthermore, the expression of OPG is regulated by multiple factors including estrogen, mechanical load, and local microenvironment, and its downregulation not only weakens the buffering capacity against RANKL but also indirectly affects the initiation of the osteogenic process, as excessive bone resorption inhibits the recruitment and activation of osteoblasts (132–134). Therefore, in the PMOP bone immune microenvironment, the RANK/RANKL/OPG axis can be viewed as a signal hub that translates the “structural remodeling + immune reprogramming” described in sections 3–4 into signals for bone loss.

Recent studies have also found that some natural compounds, such as flavonoids, can inhibit osteoclast activity and promote bone formation by regulating the RANKL/RANK/OPG axis, providing new ideas for the treatment of PMOP (135). For example, the flavonoid rutin can regulate the signaling pathways related to this axis, alleviate inflammatory responses and oxidative stress, and restore bone metabolic homeostasis, showing potential therapeutic value (136). From the perspective of bone immunology, such interventions can be understood as downstream rebalancing acting on the “osteocyte-signal hub”: without the need to directly reverse the entire immune lineage reprogramming, by reshaping the response threshold and output intensity of the RANK axis, partially severing the pathway of “immune inflammation leading to osteoclast dominance,” providing actionable targets for the precise prevention and treatment of PMOP.

### 5.2 Wnt/β-catenin, sclerostin, sclerostin and dickkopf-1 and other immune regulation of osteogenic-related pathways

The Wnt/β-catenin signaling pathway is a key regulatory mechanism in the process of bone formation, widely involved in the proliferation and differentiation of bone cells, and the maintenance of bone tissue homeostasis. This pathway promotes the differentiation of bone marrow mesenchymal stem cells into osteoblasts through the activation of β-catenin, enhancing the synthesis and mineralization of the bone matrix. This thus promotes bone formation (137). Conversely, inhibitors of the Wnt signaling pathway, such as Sclerostin and Dickkopf-1 (DKK1), prevent the accumulation of β-catenin by blocking the binding of Wnt ligands to receptors, reducing osteogenic activity and leading to impaired bone formation (138, 139).

Immune cells regulate the Wnt/β-catenin pathway by secreting various cytokines, thereby affecting the dynamic balance of bone formation. For example, the polarization state of macrophages has a significant impact on Wnt signaling (140). Based on the overall characteristics of M1/M2 polarization clarified in section 4.1, this section will only supplement parts directly related to the Wnt axis without repeating its general inflammatory effects. Studies have shown that pro-inflammatory M1 macrophages can activate the Wnt/β-catenin pathway by releasing IL-17A, promoting the inflammatory response while inhibiting the polarization of M2 macrophages; moderate activation of the Wnt/β-catenin signaling can inversely inhibit IL-17A-induced M1 polarization and promote M2 polarization, thus playing a dual regulatory role in immune-osteogenesis during bone repair (141). In addition, the Wnt signaling pathway also regulates the autophagic activity of macrophages, maintaining their immune regulatory function, which is beneficial for bone tissue regeneration (142).

Regarding inhibitors, DKK1, as a classic Wnt antagonist, also plays an important role in immune regulation. Research on tumors and the bone microenvironment suggests that DKK1 can promote the formation of an immunosuppressive microenvironment by affecting the phenotype of macrophages and other immune cells, and inhibit immune responses related to bone repair (143). Some studies indicate that DKK1 can activate downstream signals such as PI3K–AKT after binding to immune cell surface receptors (e.g., CKAP4), inducing an immunosuppressive phenotype and hindering tissue repair, suggesting that the “DKK1–immune–bone” axis may be a noteworthy negative regulatory pathway in osteoporosis and bone injury; correspondingly, blocking DKK1 is expected to restore Wnt-dependent osteogenic capacity and may indirectly improve the bone immune microenvironment (143). Sclerostin is primarily secreted by osteocytes and serves as another important inhibitory factor of the Wnt pathway, maintaining bone mass balance in bone metabolism. Sclerostin not only directly inhibits osteoblast function but also participates in the regulation of the immune system, affecting the development and differentiation of B cells (95). It reduces osteoblast activity by lowering Wnt/β-catenin signaling, promoting bone resorption, thereby affecting the stability of the bone immune microenvironment (144). Clinical studies have found that elevated levels of DKK1 and Sclerostin in the plasma of PMOP patients are closely related to decreased bone density and increased fracture risk (145). Meanwhile, activating the Wnt/β-catenin signaling pathway can promote bone repair and formation while improving the polarization state of immune cells, becoming an important target for the treatment of osteoporosis and bone injury (146). However, it should be noted that much of the existing mechanistic evidence regarding the fine associations between the Wnt/β-catenin–Sclerostin/DKK1 axis and immunological aspects such as macrophage polarization and B cell development largely comes from models of rheumatoid arthritis, tumor bone microenvironments, and bone defect repair rather than PMOP models. Therefore, the discussion of the “Wnt–immune–osteogenesis” interactive network’s role in PMOP in this section should be understood more as an integrated mechanism across diseases and models, and its specific effects in the typical PMOP bone marrow microenvironment still require further verification in subsequent studies.

### 5.3 CXCL12–CXCR4 and S1P chemotactic axes in immune cell recruitment and localization in the bone marrow

Currently, there is still limited direct evidence regarding the CXCL12–CXCR4 and S1P chemotactic axes in the context of PMOP. The following primarily draws on research findings from bone marrow-related diseases such as AML, MM, and WHIM syndrome, as a technical and research paradigm that can be transferred to PMOP. The chemokine CXCL12 and its receptor CXCR4 axis play a core role in the migration and localization of immune cells in the bone marrow. HSCs in the bone marrow rely on the support of cytokines and signaling molecules in the local microenvironment. The CXCL12/CXCR4 axis not only directly promotes the quiescent state of HSCs, but is also responsible for guiding HSCs into the stem cell “niche” in the bone marrow, achieving their correct localization and long-term maintenance (147, 148). Bone marrow stromal stem cells and endothelial cells (ECs) are the main constituent cells of these niches, secreting CXCL12 to provide directional migration signals for HSCs and their progeny blood cells (149). In addition, the CXCL12/CXCR4 axis is also involved in regulating the maintenance of immune memory cells, such as memory T cells and plasma cells, which rely on this axis to migrate to the bone marrow and interact with niche cells, ensuring their survival and functional continuity (150). In summary, the CXCL12/CXCR4 axis coordinates the generation of various blood and immune cells in the bone marrow and the maintenance of immune memory by mediating cell recruitment and subsequent maintenance signals. This reflects the key position of this chemotactic axis in stabilizing the bone immune microenvironment. Furthermore, the dysfunction of the CXCR4 receptor can lead to abnormal localization of immune cells and dynamic disturbances of cells in the bone marrow, with typical diseases such as WHIM syndrome, whose etiology is caused by mutations in the CXCR4 gene that lead to receptor endocytosis blockage, resulting in an excessive response to CXCL12 signals, causing mature neutrophils to be abnormally retained in the bone marrow, manifested as neutropenia and recurrent infections. This pathological mechanism further confirms the importance of the CXCL12/CXCR4 axis in regulating the localization of immune cells in the bone marrow (151, 152). Different mutation types have varying impacts on CXCR4 signaling, leading to diversity in clinical manifestations and signaling pathway activities, which also suggests that treatment strategies targeting CXCR4 need to consider the complexity of its signaling regulation (153). In addition to CXCL12/CXCR4, sphingosine-1-phosphate (S1P) axis is also an important regulatory factor for immune cell migration. S1P regulates the circulation of lymphocytes and their homing to the bone marrow by binding to its receptors, maintaining the spatial distribution balance of immune cells (154). Although the specific mechanisms of S1P are not detailed in this review, in the bone marrow and immune microenvironment, S1P interacts with the CXCL12/CXCR4 axis to jointly regulate the migration, localization, and functional status of immune cells, thereby affecting bone immune homeostasis.

In the tumor microenvironment, especially in multiple myeloma, the CXCL12/CXCR4 axis is exploited by malignant plasma cells to evade immune surveillance, promote the localization of tumor cells in the bone marrow, and cause bone destruction. This suggests that this axis not only maintains the normal distribution of immune cells under physiological conditions but may also be pathologically regulated under disease conditions, becoming a potential therapeutic target (155). Existing CXCR4 antagonists such as plerixafor have been used clinically for stem cell mobilization and cancer treatment, demonstrating the feasibility of intervening in this chemotactic axis to regulate the bone marrow immune microenvironment (156). Additionally, the exosome-mediated chemokine delivery mechanism also reveals the extensive role of the CXCL12/CXCR4 axis in tissue regeneration and immune cell recruitment (157). For example, Schwann cell-derived exosomes promote the migration of endogenous stem cells to the injury site by carrying CXCL12 signals, facilitating tissue repair and regeneration, indicating that this chemotactic axis also plays an important role in intercellular communication and regulation in the bone immune microenvironment (158).

In summary, the CXCL12–CXCR4 and S1P chemotactic axes play pivotal roles in the recruitment, migration, and localization of immune cells in the bone marrow. These axes ensure the dynamic balance and functional stability of the bone immune microenvironment. They coordinate the maintenance of hematopoietic stem cells, the preservation of immune memory, and the spatial distribution of immune cells through complex signaling networks, thereby affecting bone metabolism and bone immune homeostasis, and serve as important entry points for studying the pathological mechanisms of PMOP and developing new therapeutic strategies. Currently, direct mechanistic studies on the CXCL12–CXCR4 and S1P axes in the context of PMOP are still relatively limited, with existing evidence mostly coming from bone marrow-related diseases such as AML, MM, and WHIM syndrome, as well as the field of stem cell mobilization. The role of these axes in potentially participating in the localization of immune cells and maintaining low-grade inflammation in PMOP is primarily based on cross-disease and cross-model analogical inference, and lacks systematic verification at the bone marrow level in PMOP. Combining existing evidence regarding HSC aging, MSC senescence, and increased BMAT, it is speculated that the remodeling of these chemotactic axes is involved in the changes in immune cell localization in the bone marrow and the maintenance of low-grade chronic inflammation in PMOP.

### 5.4 Bone marrow aging, aging-associated secretory phenotype, and chronic low-grade inflammation

The previous sections 3.1–3.3 have described HSC/MSC aging, myeloid bias, and BMAT expansion from the perspective of structure and cell lineage, while sections 4.1–4.3 discuss the reprogramming of innate/adaptive immune lineages and the imbalance of cytokine networks. This section, without repeating the structural and lineage details mentioned above, specifically organizes how bone marrow aging and the SASP continuously drive this multi-layered imbalance from a “temporal dimension,” thereby placing PMOP within the broader framework of inflammaging.

As a microenvironment that is crucial to both hematopoiesis and the skeletal system, the cellular population of bone marrow, particularly MSCs and HSCs, undergoes significant aging changes with increasing age. Cell aging refers to the irreversible growth arrest that cells experience, accompanied by a specific secretory phenotype, namely SASP. SASP mainly includes various pro-inflammatory cytokines, chemokines, and proteases, and the continuous release of these factors not only disrupts local tissue homeostasis but also triggers a chronic low-grade inflammatory state known as “inflammaging” (159–161). Bone marrow aging leads to the accumulation of SASP factors, maintaining a chronic, low-grade but persistent inflammatory state, which plays an important role in the development of osteoporosis (162).

More specifically, the shift in osteogenic-adipogenic differentiation of MSCs described in 3.1 and the expansion of BMAT in 3.3 can be seen as the terminal phenotype of bone marrow aging at the structural and lineage levels; while in the temporal dimension, SASP continuously releases factors such as IL-6, IL-1β, and TNF-α, which simultaneously provide sustained stimulation for the pro-inflammatory polarization of macrophages, the imbalance of Th17/Treg, and the reconstruction of B cell subsets described in 4.1–4.2, while reinforcing the RANKL/OPG imbalance and the cytokine cluster amplification effect summarized in 4.3, ultimately leading to a resettlement of osteoclast-dominant homeostasis at the signaling pathway level, such as the RANK axis described in 5.1 (163). In other words, bone marrow aging and its accompanying SASP are not a single pathological link but a “slow but constant” driving force that runs through multiple levels of sections 3–5.

The composition of SASP factors is complex, including various pro-inflammatory cytokines such as IL-6, IL-1β, and TNF-α, which promote the sustained presence of inflammatory responses by activating signaling pathways like NF-κB, interfere with the function of BMSCs, and induce more cells into a state of senescence, forming a self-reinforcing feedback loop (164, 165). In this context, the regenerative potential of bone marrow stromal cells is continuously interfered with by SASP, and bone remodeling is gradually reset to a “new homeostasis” dominated by bone resorption, which also explains why, clinically, even if some reversible risk factors are corrected in the short term, the improvement in bone mass and bone microstructure in PMOP patients remains limited—the “chronic driving force” maintained by bone marrow aging and SASP has not been fundamentally reversed.

Currently, research on bone marrow aging and SASP is gradually deepening, and exploring their regulatory mechanisms and intervention methods has become a new direction for the prevention and treatment of osteoporosis. Some anti-aging strategies, such as drugs targeting apoptotic senescent cells (senolytics) and drugs modulating SASP (senomorphics), show potential application value, as they can alleviate chronic inflammation in the bone marrow microenvironment and improve the bone metabolic environment by clearing senescent cells or inhibiting the secretion of SASP factors, thereby promoting skeletal health (166, 167). In addition, a combination of various strategies such as lifestyle interventions, drug treatments, and molecular targeted therapy is expected to mitigate the negative impacts of bone marrow aging and delay the progression of osteoporosis (168).

**Figure 5:**
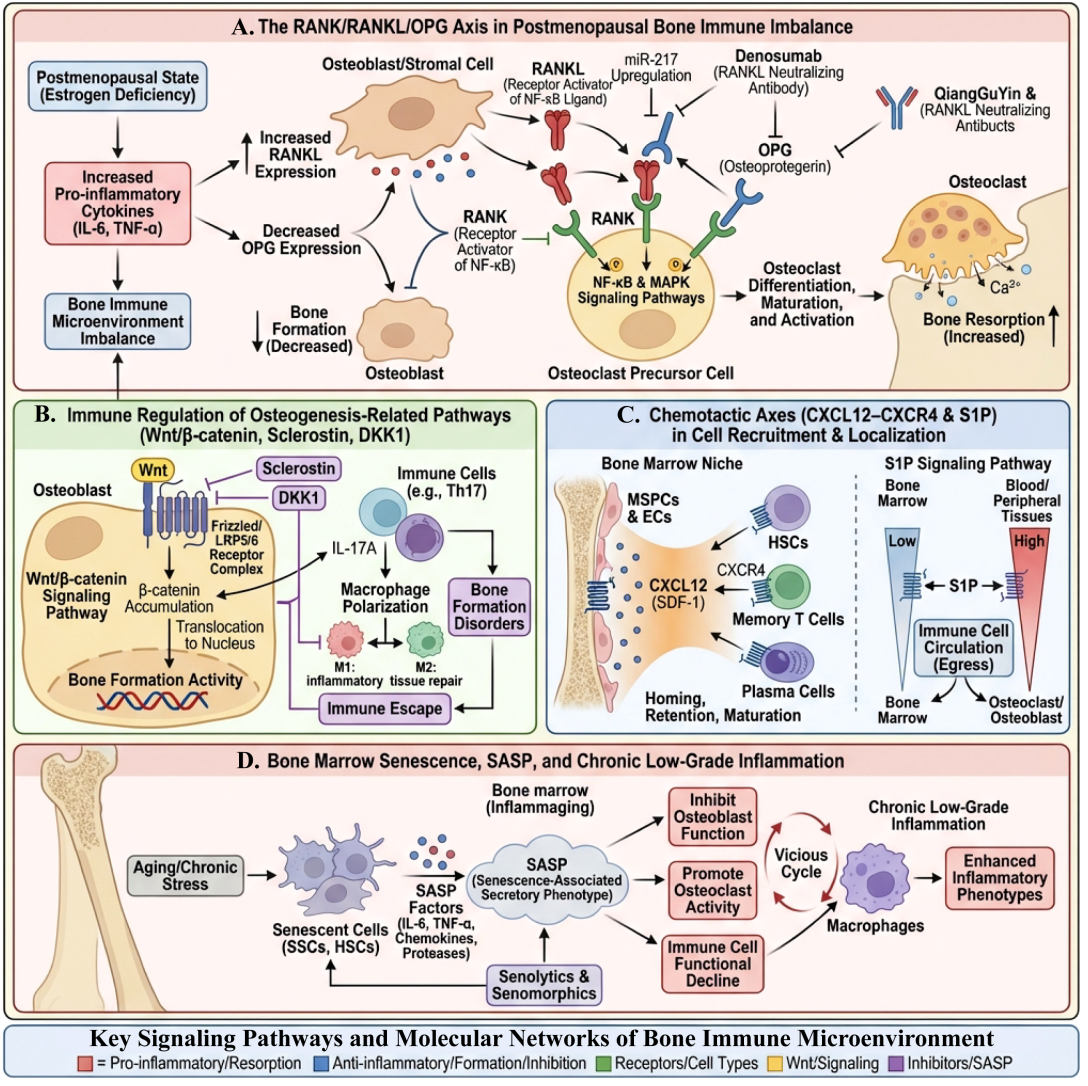
Schematic diagram of key signaling pathways and molecular networks in the postmenopausal bone immune microenvironment. **(A)** Estrogen deficiency and increased pro-inflammatory factors (IL-6, TNF-α) induce elevated expression of RANKL in osteoblasts and stromal cells, along with downregulation of OPG. This activates NF-κB and MAPK signaling pathways, promoting the differentiation and maturation of osteoclast precursors and enhancing bone resorption. Agents such as denosumab and bisphosphonates exert anti-resorptive effects by neutralizing RANKL. **(B)** Immune cells influence bone formation by regulating osteogenic-related pathways, including Wnt/β-catenin, sclerostin, and DKK1. Th17 cells secrete IL-17A, and an imbalance in macrophage M1/M2 polarization leads to inhibition of β-catenin nuclear translocation, resulting in impaired bone formation. **(C)** The CXCL12–CXCR4 and S1P chemotactic axes regulate the homing, retention, and peripheral migration of HSCs, T and B cells, and plasma cells within the bone marrow microenvironment. This affects the recruitment and localization of bone immune cells. **(D)** Aging and chronic stress induce senescence and SASP in bone marrow stromal cells and hematopoietic stem cells. These cells continuously release various pro-inflammatory factors and proteases, causing chronic low-grade inflammation, inhibiting osteoblast function, and promoting osteoclast activity, thereby forming a vicious cycle of “inflammation–bone loss.” The color-coded area at the bottom summarizes key modules in the bone immune microenvironment, including pro-inflammatory/pro-resorptive factors, anti-inflammatory/pro-osteogenic factors, receptors and cell types, Wnt signaling components, and inhibitors/SASP.

In summary, bone marrow aging maintains and exacerbates a chronic low-grade inflammatory state through the secretion of SASP factors, becoming an important mechanism driving osteoporosis, especially PMOP. A deeper understanding of the interaction between bone marrow aging and SASP and their impact on the bone immune microenvironment is of great significance for developing targeted therapeutic strategies and improving the prognosis of osteoporosis patients (**Figure 5**). From a broader systemic perspective, local bone marrow aging and SASP are also important components of inflammaging, and their systemic effects will be further discussed in section 6.2.

## 6 Systemic factors in the upstream regulation of the bone immune microenvironment

### 6.1 Estrogen deficiency and endocrine-immune interaction: ER signaling and immune cell reprogramming

Estrogen exerts a key regulatory role in the bone marrow microenvironment through its receptors (ER), directly affecting immune cell function and bone metabolic balance (169). Estrogen receptors are classified into two subtypes, ERα and ERβ, which mediate the activation state and phenotypic changes of immune cells by regulating downstream gene transcription and multiple signaling pathways (170, 171). For example, estrogen can regulate the metabolic state and secretion of inflammatory factors by T cells and macrophages, promoting the maintenance of an anti-inflammatory phenotype. This indirectly regulates the balance between osteoclasts and osteoblasts and maintains bone homeostasis (172).

After menopause, estrogen levels significantly decline, leading to an imbalance in immune regulation mediated by ER, making immune cells more prone to reprogramming toward a pro-inflammatory phenotype. Studies have shown that estrogen deficiency not only induces immune cells to produce more pro-inflammatory cytokines (such as TNF-α, IL-1β, IL-6), but also promotes the activation of bone-resorbing cells (osteoclasts), enhancing the bone resorption process and causing osteoporosis (21, 173). In addition, the dysbiosis of gut microbiota associated with estrogen deficiency also exacerbates bone loss through the interaction between the immune system and bone metabolism; changes in microbial composition and impaired intestinal barrier can promote the entry of microbe-associated molecular patterns (MAMPs) into the bloodstream, activating innate immune cells in the bone marrow and maintaining low-grade chronic inflammation (174). The specific characteristics of macrophage M1/M2 polarization, Th17/Treg imbalance, and changes in B cell lineages have been detailed in sections 4.1–4.2; therefore, this section emphasizes the upstream triggering role of “estrogen–ER–immune reprogramming” without repeating the mechanisms at the cellular lineage level.

The regulation of immune cell metabolism and function by estrogen also involves endoplasmic reticulum stress (ERS)-related pathways. ERS affects the phenotype and inflammatory state of immune cells by regulating their metabolic reprogramming and secretory profiles (175). Under conditions of estrogen deficiency, ERS-related signals such as the IRE1α/XBP1 pathway are activated, promoting the abnormal expression of pro-inflammatory factors and immune-suppressive factors, further exacerbating the imbalance of the immune microenvironment and osteoporosis (176, 177). It should be noted that current evidence regarding ER-mediated estrogen–immune–bone axis reprogramming mainly comes from animal models and *in vitro* cell experiments. Direct validation in PMOP patients remains limited. This limited clinical validation indicates that the mechanistic inferences discussed in this review should be considered as a framework of hypotheses rather than definitive conclusions confirmed in clinical settings.

In summary, estrogen plays a pivotal regulatory role in immune cell function and bone metabolism through ER-mediated signaling pathways. After menopause, estrogen deficiency leads to weakened ER signaling, which causes immune cells to shift towards a pro-inflammatory phenotype. This shift enhances osteoclast activity, promotes bone resorption, and ultimately results in osteoporosis.

### 6.2 Inflammatory aging and PMOP

Inflammaging is a chronic low-grade systemic inflammatory state that occurs with aging and is one of the important upstream driving forces affecting the bone immune microenvironment (178, 179). In postmenopausal women, the decline in estrogen levels, combined with age-related immune senescence, makes key immune cell subpopulations such as bone marrow macrophages and osteal macrophages (bone-resident macrophages) more prone to acquiring pro-inflammatory and senescence-associated transcriptional profiles, thereby amplifying the existing baseline of bone marrow inflammation (180). The continuously released pro-inflammatory mediators (such as IL-6, TNF-α, and others) not only directly damage osteogenic-related cells (such as MSCs and osteoblasts) but also induce a persistent shift in bone metabolism towards bone resorption surpassing bone formation by promoting osteoclast generation and activity (162). In the PMOP model, the clearance of senescent cells or oxidative stress-related subpopulations of bone marrow macrophages can partially alleviate bone loss. This further supports that inflammaging is one of the key pathological bases of PMOP (181, 182).

Unlike Section 5.4, which focuses on “local bone marrow senescence and SASP,” this section emphasizes the systemic level of inflammaging. The impact of inflammaging on the bone immune microenvironment is not limited to the local bone marrow. The SASP factors released by senescent MSCs, T cells, and other immune subpopulations enter circulation and maintain low-grade inflammation throughout the body. This persistent inflammation promotes imbalance in the gut-bone-immune axis and forms a pathological loop of “systemic inflammaging – bone marrow inflammation – bone loss” (183, 184). Furthermore, estrogen deficiency also increases intestinal permeability and microbial displacement by affecting gut microbiome and barrier function, which further amplifies the systemic inflammatory response (185).

From a clinical perspective, multiple cohort studies have observed that systemic inflammatory indicators such as the systemic immune-inflammation index (SII) and C-reactive protein (CRP) are positively correlated with the occurrence of PMOP and fracture risk, suggesting that these inflammatory markers can serve as auxiliary indicators for assessing osteoporosis risk and disease progression (186, 187).Notably, intervention strategies targeting inflammaging are emerging as a new direction in the field of PMOP. In addition to senolytics and senomorphics—agents that selectively eliminate or modulate senescent cells—some active ingredients of traditional Chinese medicine and plant extracts have also shown potential in modulating inflammaging (188). For example, Icariin can promote the recovery of osteoblast function by activating autophagy and inhibiting the inflammatory senescence phenotype of bone marrow macrophages, thereby alleviating bone loss (189). Extracts of Cimicifuga racemosa have been confirmed to reshape macrophage immune metabolism and inhibit proinflammatory signals, exhibiting potential anti-inflammatory aging effects (190). These strategies provide new ideas for comprehensive intervention in PMOP, by regulating the bone immune microenvironment from the perspective of systemic inflammaging. However, current evidence regarding the association between inflammaging and PMOP risk is supported by several large-sample epidemiological studies based on inflammatory indicators such as CRP and SII. Mechanistic evidence targeting specific senescent immune cell subpopulations and their SASP mainly comes from animal models and *in vitro* experiments. At the same time, research on the intervention effects of natural products like Icariin and Cimicifuga racemosa on inflammaging is still largely in the preclinical stages. Therefore, the relevant content of this section is more suitably understood as mechanistic integration and intervention ideas with clinical relevance, rather than a validated standard treatment pathway.

In summary, inflammaging exacerbates the process of bone loss in PMOP by maintaining chronic low-grade systemic inflammation, altering the composition and function of bone marrow and systemic immune cells, and promoting the continuous release of proinflammatory factors. Compared to the emphasis on local bone marrow senescence mechanisms and the connotation of SASP in Section 5.4, this section supplements the upstream background from the perspectives of systemic inflammaging, clinical inflammatory indicators, and systemic intervention strategies, logically connecting the two perspectives rather than simply repeating them.

### 6.3 Gut-bone-immunity axis: Gut microbiome, barrier function, and bone marrow immune status

The imbalance of the gut microbiome has a profound impact on systemic immune responses and bone metabolism. The surfaces exposed to the external environment in mammals are covered by a large number of symbiotic microorganisms; the gut microbiome is one of the richest ecosystems which regulate the activity of important endocrine hormones related to bone development and metabolism, such as parathyroid hormone (PTH) (191, 192). Studies have shown that gut microorganisms mediate the effects of PTH on bone resorption and bone formation through their metabolites, such as short-chain fatty acids (SCFAs), and through the migration of immune cells; this reveals the communication mechanism between gut microorganisms and bone marrow cells (193). Furthermore, changes in the gut microbiome or antibiotic-induced depletion of the microbiome can weaken the migration of natural killer (NK) cells and Th1 cells from the gut to the bone marrow, leading to accelerated tumor growth in bone tissue. This indicates that the migration of gut immune cells plays a key role in maintaining immune homeostasis in the bone marrow and bone metabolism (194). With aging, both the gut microbiome and the hematopoietic system undergo changes. Gut microorganisms influence the regulation of HSCs and the generation of immune cells through various mechanisms, including their metabolites, MAMPs, and Toll-like receptor (TLR) signaling pathways. These mechanisms indirectly regulate the immune environment of the bone marrow (195). These findings suggest that postmenopausal women with gut microbiome–SCFAs imbalance may amplify the preferential effects of PTH and other endocrine factors on bone resorption, thereby increasing the risk of bone loss.

The impairment of gut barrier function is a critical factor facilitating the entry of pro-inflammatory factors into circulation within the gut-bone-immune axis. Patients with PMOP often exhibit gut microbiome dysbiosis accompanied by gut barrier disruption. This leads to the translocation of bacteria and their products, such as lipopolysaccharides (LPS), across the gut barrier into the bloodstream; this triggers systemic chronic low-grade inflammation, promoting bone resorption and bone loss (196). The expression of tight junction proteins in the gut barrier is reduced, accompanied by increased gut permeability, which promotes inflammatory responses and abnormal activation of immune cells, further affecting the immune microenvironment of the bone marrow (197). Additionally, damage to the gut barrier results in a reduction of beneficial bacteria and their metabolites, such as butyrate, which affects the function of Treg and promotes an increase in inflammatory Th17 cells in the bone marrow, thereby activating the bone resorption process (198, 199). Studies have found that gut microbiota, such as Akkermansia muciniphila, have a protective effect on osteoporosis by regulating the gut barrier and immune responses, with their abundance closely related to bone metabolic status (200). These results collectively point to a potential closed loop related to PMOP. Specifically, the imbalance of gut microbiota and barrier damage not only triggers systemic inflammation but can also directly reprogram the immune status of the bone marrow through specific immune axes.

The imbalance of gut microbiota not only affects local gut immunity but also mediates immune reprogramming of the gut-bone axis through the migration of immune cells and the circulation of metabolic products. Gut immune cells, such as bone marrow-derived dendritic cells and macrophages, undergo phenotypic changes under the influence of gut microorganisms. These changes regulate the immune microenvironment of the bone marrow and affect the bone remodeling process (201). Furthermore, gut microbial metabolites can act on the bone marrow through the bloodstream, regulating the differentiation and function of immune cells within the bone marrow and maintaining skeletal homeostasis (202). Therefore, regulating the gut microbiome and maintaining gut barrier function have become potential strategies for improving osteoporosis and related bone immune diseases. In recent years, treatment research targeting the gut-bone-immune axis has gradually increased, based on probiotics, prebiotics, dietary interventions, and fecal microbiota transplantation, showing good bone protective effects and immune regulatory potential (203, 204). From the perspective of PMOP prevention and treatment, intervention in the gut-bone-immune axis is particularly suitable as a systemic, long-term auxiliary strategy. On one hand, by reconstructing the microbiota-barrier-metabolite network, it alleviates immune imbalance against the background of inflammatory aging. On the other hand, it complements anti-bone resorption and anabolic drugs, with the potential to achieve three-dimensional coordinated regulation involving endocrine, immune, and metabolic systems (205, 206). However, it should be emphasized that much of the evidence regarding the “microbiota-barrier-LPS-Th17/Treg-bone loss” loop and the role of specific strains (such as *A. muciniphila*) in bone protection still comes from animal models and small exploratory studies. Large-scale prospective cohort and interventional trials specifically targeting postmenopausal osteoporosis/PMOP women are still lacking. Therefore, the discussion of the potential role of the gut-bone-immune axis in PMOP in this section is more of a comparative inference and intervention hypothesis based on cross-disease and cross-model evidence, awaiting further validation and refinement through high-quality clinical and translational research in the future.

**Figure 6:**
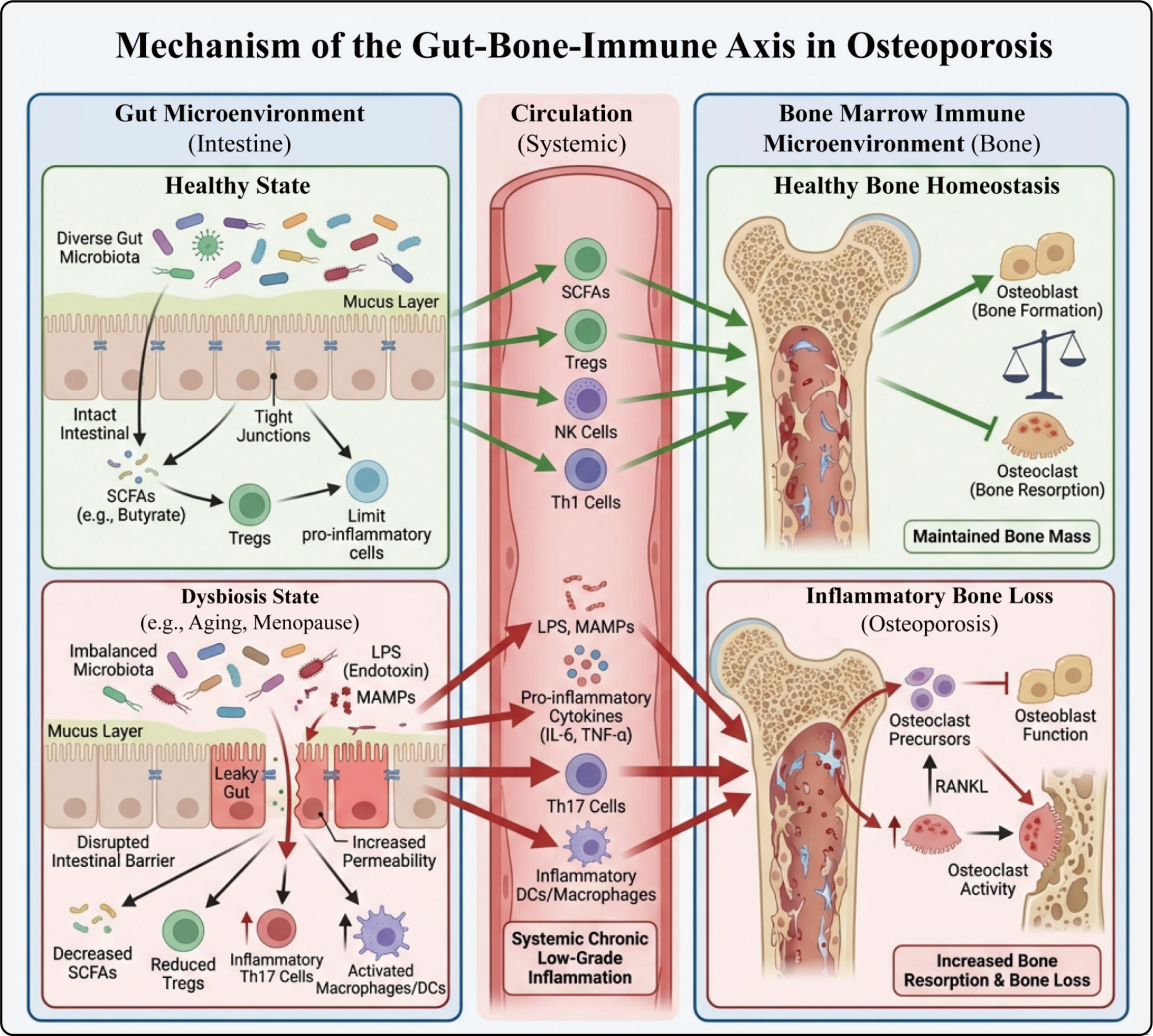
Schematic diagram of the role of the gut-bone-immune axis in the mechanism of osteoporosis. Left: In a healthy state, the diversity of gut microbiota maintains an intact intestinal mucosal barrier and tight junctions, producing SCFAs, inducing Treg, and inhibiting pro-inflammatory immune cells. This process limits the entry of inflammatory factors into the bloodstream. With aging, menopause, or other triggers, dysbiosis occurs, leading to damage to the intestinal mucosal barrier, increased permeability, reduced SCFAs, downregulation of Treg, and activation of Th17 cells and inflammatory dendritic cells and macrophages. Consequently, endotoxins (LPS, MAMPs) and pro-inflammatory cytokines enter the circulation in large quantities, forming systemic low-grade chronic inflammation. Middle and right: In the systemic circulation, SCFAs and regulatory immune cells help maintain immune homeostasis in the bone marrow and support normal bone remodeling. In contrast, when LPS, MAMPs, pro-inflammatory factors such as IL-6 and TNF-α, as well as Th17 cells and inflammatory dendritic cells and macrophages enter the bone marrow microenvironment, they promote RANKL upregulation and activation of osteoclast precursors, inhibit osteoblast function, and disrupt the balance between bone formation and bone resorption. This imbalance results in decreased bone mass and inflammatory osteoporosis.

In summary, the imbalance of the gut microbiome affects bone metabolism and bone health by influencing gut barrier function and promoting the translocation of inflammatory factors and microbial products into the bloodstream. It also regulates the immune microenvironment of the bone marrow. The gut-bone-immune axis serves as a key regulatory network for immune-related bone diseases such as osteoporosis, providing new therapeutic targets and strategies for clinical practice. To advance this field, future efforts should further deepen the understanding of its molecular mechanisms and promote the development of precision medicine and personalized treatment approaches (**Figure 6**).

## 7 The bone immune microenvironment as a therapeutic target for PMOP

In terms of treatment strategies, this review aims to propose a framework for bone immune therapy for PMOP based on the concept of “stratified intervention,” which refers to tailored therapeutic approaches targeting different levels of disease mechanisms. Overall, existing and potential intervention methods can be summarized into three progressive levels. The first level includes “traditional bone-targeting drugs” primarily acting on osteoclasts and osteoblasts, which reshape the bone immune microenvironment to varying degrees. Examples include bisphosphonates, denosumab, SERMs, PTH-like drugs, and romosozumab. Their core mechanism involves inhibiting osteoclasts and/or promoting osteoblasts, thereby indirectly regulating inflammation and immune status. The second level focuses on immune-targeted interventions directly targeting immune cells or cellular factors, such as anti-TNF, IL-6, and IL-17 biologics, as well as cell or drug therapies targeting regulatory lymphocytes like Treg and Breg cells. These interventions aim to actively correct the pro-inflammatory–anti-inflammatory imbalance and reshape the bone immune axis, which refers to the dynamic interactions between immune cells and bone cells that regulate bone remodeling. Finally, the third level addresses the overall regulation of the bone marrow microenvironment. This includes senolytic and antioxidant interventions; regulation of BMAT; optimization of the vascular–nerve–matrix axis; and systemic comprehensive interventions such as modulation of the gut–bone axis. These strategies strive to reverse the trained inflammatory marrow state—a persistent pro-inflammatory condition of the bone marrow—by restoring microenvironmental homeostasis. The following sections will discuss these three intervention strategies in detail.

### 7.1 Reinterpretation of the bone immunological mechanisms of existing anti-osteoporosis drugs

The therapeutic mechanisms of existing anti-osteoporosis drugs are not limited to their direct effects on bone cells; an increasing number of studies reveal their regulatory roles in the bone immune microenvironment, emphasizing the central position of the immune system in the bone remodeling process. Immune cells and the cytokines they secrete regulate the activity of osteoclasts and osteoblasts, affecting the balance of bone metabolism and forming the basis of bone immunology.

Bisphosphonates, the widely used anti-osteoporosis drugs in clinical practice, primarily exert their anti-osteoporosis effects by inducing osteoclast apoptosis and inhibiting bone resorption (207). Recent studies have shown that bisphosphonates also have the potential to regulate the bone immune microenvironment. Firstly, bisphosphonates can reduce the activity of osteoclasts and decrease the release of pro-inflammatory factors related to bone resorption, thereby alleviating the inflammatory state of the bone microenvironment (208). On the other hand, bisphosphonates affect immune cell function by inhibiting the activation of pro-inflammatory macrophages and promoting the increase of Treg. This regulation of immune balance helps protect and repair bone quality (209). In addition, the antigen fragments released from osteoclast apoptosis induced by bisphosphonates can be taken up by antigen-presenting cells, activating specific immune responses and further regulating the activity of immune cells, thus influencing bone remodeling and metabolism (210). These mechanistic insights provide a new immunological perspective on the bone protective effects of bisphosphonates, suggesting that they are not merely simple bone resorption inhibitors. Transitioning to another class of drugs, Denosumab is a humanized monoclonal antibody that specifically targets RANKL (receptor activator of nuclear factor κB ligand). It blocks the binding of RANKL to the RANK receptor and inhibits the formation and function of osteoclasts (211, 212). RANKL is not only a key factor in osteoclast differentiation but also an important signaling molecule for various immune cells such as T cells, B cells, and dendritic cells (213). Denosumab indirectly regulates the activity of immune cells and their communication with bone cells by inhibiting RANKL, restoring balance within the bone immune microenvironment (214). Studies show that Denosumab treatment can reduce the production of pro-inflammatory cytokines, inhibit immune-related bone resorption processes, and improve bone density and bone quality. Furthermore, the application of Denosumab in immune-mediated bone diseases such as rheumatoid arthritis further confirms its mechanism of regulating immune-bone cell interactions (215). However, Denosumab has a minimal impact on the gastrointestinal microbiota, suggesting that its immunomodulatory effects are primarily limited to the local immune environment of the bone (216). Selective estrogen receptor modulators (SERMs) regulate bone metabolism and the immune response by mimicking the bone-protective effects of estrogens. Estrogen deficiency is a major trigger for PMOP, leading to an increase in pro-inflammatory cytokines such as TNF-α and IL-6 in the immune system, promoting osteoclast activity and bone resorption (217). SERMs inhibit the expression of these pro-inflammatory factors by activating estrogen receptors, alleviating the inflammatory state of the bone, while promoting the activity of regulatory immune cells to balance the bone immune microenvironment (206). PTH and its analogs, such as Teriparatide, promote bone formation by directly activating osteoblasts and regulating the release of cytokines from immune cells, such as increasing the anti-inflammatory cytokine IL-10 and IFN-γ. These actions promote bone regeneration and inhibit bone resorption (218, 219). These immunomodulatory effects help restore the dynamic balance of bone metabolism and improve the bone microenvironment. Romosozumab is a monoclonal antibody against sclerostin that promotes bone formation and inhibits bone resorption. Sclerostin, which is secreted not only by osteoblasts, also participates in regulating immune cell function (220, 221). Romosozumab regulates the Wnt signaling pathway, thus affecting the activity of immune cells, improving the inflammatory state, and promoting the repair of the bone immune microenvironment (222). Through these mechanisms, the bidirectional regulatory effects of Romosozumab make it a powerful drug for the treatment of osteoporosis in bone immunology (223).

In summary, existing anti-osteoporosis drugs not only inhibit osteoclast activity and promote osteoblast function but also influence the overall balance of bone metabolism by regulating immune cells and the cytokines they secrete within the bone immune microenvironment. In-depth research on these bone immunological mechanisms provides a theoretical basis and practical guidance for developing more precise and effective treatment strategies for osteoporosis. It also offers important evidence for reinterpreting the mechanisms of action of anti-RANKL drugs such as Denosumab from the perspective of bone immunology. Future drug development should pay greater attention to the cross-regulation between the immune system and bone metabolism, thereby promoting innovative advances in the field of bone immunology.

### 7.2 Emerging immune targeting strategies

In recent years, research on the imbalance of the bone marrow immune microenvironment in osteoporosis, especially in PMOP, has driven the development of various emerging immune-targeted therapeutic strategies. The occurrence of PMOP is closely related to inflammatory responses, with inflammatory cytokines such as TNF, IL-6, and IL-17 playing key roles in the imbalance between bone resorption and bone formation. Therefore, interventions targeting these inflammatory pathways show potential for bone protection.

Firstly, treatments targeting inflammatory cytokines such as anti-TNF, anti-IL-6, and anti-IL-17 have been shown to effectively regulate the bone marrow immune microenvironment in osteoporosis (224). TNF, as a pro-inflammatory factor, promotes the differentiation and activity of osteoclasts, exacerbating bone resorption. Inhibiting its signaling pathway using anti-TNF antibodies not only reduces bone resorption but also improves bone metabolic balance (225). Similarly, IL-6 and IL-17 promote bone loss in the bone marrow by activating osteoclast precursors and inducing the release of pro-inflammatory cytokines. Inhibiting these pathways can alleviate inflammatory responses and suppress excessive activation of osteoclasts, thereby exerting bone-protective effects (226). It is important to emphasize that current clinical evidence regarding these biologic agents in directly improving the bone marrow immune microenvironment and reversing bone loss in PMOP patients is still limited. However, the inflammatory pathways they target highly overlap with the immune networks that have been proven to be abnormally activated in the bone marrow of PMOP patients. This overlap provides valuable insights and directions for future exploration of immune-targeted therapeutic strategies for osteoporosis (136). Furthermore, recent studies have shown that the regulation of inflammatory pathways also involves the metabolic and phenotypic reprogramming of myeloid cells and lymphocytes in the bone marrow, offering a new perspective for immune therapies targeting metabolic pathways (227).

Secondly, regulating the functions of immune regulatory cells such as Treg and Breg is an important strategy for restoring the balance of the bone marrow immune microenvironment (228). Treg and Breg cells play central roles in maintaining immune tolerance and suppressing inflammatory responses. Their dysfunction can lead to the excessive activation of pro-inflammatory T cell and B cell subpopulations that promote bone resorption, further exacerbating bone loss. Studies have found that both the quantity and function of Treg cells are impaired in PMOP patients, while promoting the expansion and activation of Treg cells helps to inhibit osteoclast formation and reduce bone resorption (229). Similarly, Breg cells maintain bone immune homeostasis by secreting anti-inflammatory cytokines, including IL-10, which inhibit the activity of pro-inflammatory cells. Immune therapies targeting the regulation of these cell subpopulations, using small molecule drugs or cell therapies, have shown potential bone-protective effects (230). However, current evidence regarding Treg/Breg-related cell therapies or small molecule interventions in the context of PMOP remains largely at the stage of conceptual extrapolation and early experimentation. Existing data are primarily derived from studies on rheumatoid arthritis, osteoarthritis, or tumor immunology, which necessitates more targeted validation in models of estrogen deficiency and postmenopausal populations (231).

In addition, the metabolic reprogramming of immune cells in the bone marrow immune microenvironment has become a new therapeutic target as well. For example, the metabolic status of MDSCs and macrophages derived from bone marrow affects their immunosuppressive functions and bone resorptive effects. Regulating the metabolic pathways of these cells can modulate the immune microenvironment by enhancing anti-inflammatory responses and inhibiting pro-resorptive immune activity, thereby slowing down bone loss (232, 233). The combination of nanotechnology and genetic engineering approaches, such as the intra-bone marrow injection of engineered probiotics or genetically modified hematopoietic stem cells, can precisely regulate the bone marrow immune microenvironment, enhance the activity of anti-inflammatory cells, and suppress bone resorptive immune cells (234, 235). For PMOP, these metabolic reprogramming and nano/gene engineering strategies are currently mostly in the conceptual exploration and early experimental stages, and they theoretically align well with the “trained inflammatory bone marrow” hypothesis, possessing strong translatability and research value (236).

In summary, anti-TNF and anti-IL-6/IL-17 treatments targeting inflammatory pathways, combined with the regulation of Treg/Breg cell functions and suppression of bone resorptive T/B cell subpopulations, constitute emerging directions for immune therapy in PMOP. Additionally, immune intervention strategies based on metabolic reprogramming represent a promising avenue. These strategies rely on key immune pathways, such as TNF-α, IL-6, IL-17 signaling, and regulatory T/B cell modulation, which have been partially validated in rheumatoid arthritis, tumors, and other immune-mediated diseases. They also significantly overlap with the key cell populations and signaling networks involved in the immune reprogramming of PMOP bone marrow, providing a reference path for the future development of truly targeted immune intervention plans for PMOP. Based on rigorous disease-specific validation, integrating molecular targeting, cell therapy, and multi-level immune regulation through nanotechnology is expected to significantly enhance the therapeutic effects of PMOP and improve patients’ bone health and quality of life.

### 7.3 Comprehensive intervention targeting the bone marrow microenvironment

Comprehensive intervention strategies targeting the bone marrow microenvironment show significant potential. They regulate the bone immune microenvironment in PMOP. The aging and inflammatory state of the bone marrow microenvironment not only affects the function of HSCs but also disrupts the balance of bone remodeling, leading to the occurrence and progression of osteoporosis. These intervention measures mainly include the application of senolytics and antioxidants, regulation of bone marrow fat content, promotion of angiogenesis, and the synergistic effects of lifestyle and nutritional interventions (237, 238). Firstly, senolytics and antioxidants restore homeostasis within the bone marrow microenvironment by alleviating aging and chronic inflammation. With age, the bone marrow experiences a decline in cell function, an increase in inflammatory factor levels, and changes in the extracellular matrix. These changes lead to immune cell dysfunction and bone metabolic disorders (239, 240). Senolytics can target senescent cells and promote their clearance. They also reduce the release of pro-inflammatory cytokines such as TNF-α, IL-1β, and IL-6; thereby, they diminish the negative impact of the inflammatory microenvironment on hematopoiesis and bone immunity in the bone marrow (241, 242). Antioxidants help restore immune regulatory functions and bone-forming capacity within the bone marrow by clearing excess reactive oxygen species, alleviating oxidative stress, and protecting the function of BMSCs and immune cells (243). Moreover, nanocomposite materials with stimuli-responsive release systems offer new approaches for the targeted delivery of senolytics and antioxidants to the bone marrow, potentially achieving multi-target regulation of the microenvironment (244).

Secondly, regulating bone marrow fat content and promoting angiogenesis are key factors in improving the bone immune microenvironment. BMAs not only occupy space in the bone marrow but also influence bone metabolism and immune cell function by secreting fatty acids, cytokines, and chemokines (245). Increased bone marrow fat content is closely associated with osteoporosis, as it promotes bone resorption while inhibiting bone formation. By regulating adipocyte differentiation and metabolism, reducing bone marrow fat accumulation can improve the bone immune microenvironment. Furthermore, promoting angiogenesis in the bone marrow helps provide nutrients and oxygen to bone immune cells and hematopoietic stem cells, maintaining their function and activity (246, 247). For example, strategies using bioactive materials to promote vascular and nerve regeneration, combined with endogenous or externally applied immune regulatory mechanisms, can achieve overall optimization of the bone marrow microenvironment. Furthermore, specific probiotics and their metabolites (such as butyrate) can influence the immune microenvironment of the bone marrow by regulating the gut-bone axis, promoting bone development and delaying bone loss (248).

Finally, lifestyle and nutritional interventions play a synergistic role in regulating the bone immune microenvironment. Regular physical exercise not only increases bone mineral density but also activates immune cells in the bone marrow, promoting the functional recovery of key immune subpopulations such as T cells, thereby improving the bone immune microenvironment (249). In terms of nutrition, optimizing the intake of calcium, vitamin D, and nutrients with anti-inflammatory properties helps maintain bone immune homeostasis and skeletal health (250). Additionally, a diet rich in antioxidants and anti-inflammatory components can alleviate inflammation in the bone marrow and, when combined with lifestyle adjustments, is expected to delay the progression of osteoporosis (251).

In summary, comprehensive interventions targeting the bone marrow microenvironment alleviate aging and inflammation through senolytics and antioxidants. They also regulate bone marrow fat and promote angiogenesis to improve immune and bone metabolic environments. These approaches achieve multi-layered, multi-target synergistic regulation in conjunction with lifestyle and nutritional interventions. These strategies not only help restore the balance of the bone immune microenvironment but also provide a new theoretical basis and therapeutic approaches for the prevention and treatment of PMOP. In the future, these strategies, combined with smart materials and precision medicine technologies, are expected to promote the development of clinical translation and personalized treatment (**Table 1**).

**Table 1:** Overview of stratified intervention strategies targeting the bone immune microenvironment in PMOP.

Intervention level	Represents drugs/strategies	Main target/object of action	Regulatory mechanism of bone immune microenvironment	Application characteristics and limitations of PMOP	References
Traditional bone-targeted drugs	Bisphosphonates (BPs)	Osteoclasts and their precursors; Hydroxyapatite on the bone surface	① Induce apoptosis of osteoclasts and inhibit bone resorption; ② Reduce the release of osteoclast-related pro-inflammatory factors and alleviate local inflammation; ③ Inhibit the activation of pro-inflammatory macrophages and promote the increase of Treg; The antigen fragments produced by osteoclast apoptosis are taken up by APC, reshaping the specific immune response.	The first-line drug with the most solid evidence; It provides definite protection for bone mass, but long-term application poses risks such as mandibular osteonecrosis and atypical fractures. The mechanism of immunological action is still under in-depth interpretation.	(207–210)
	Denosumab (Anti-RANKL monoclonal antibody)	Rankl-rank axis Osteoclast precursors and T/B cells, DCS and other immune cells expressing RANKL	① Block the binding of RANKL-RANK to inhibit the formation and function of osteoclasts; ② Down-regulate the production of pro-inflammatory cytokines and inhibit immune-mediated bone resorption; ③ Regulate RANKL-dependent signaling among T cells, B cells and dendritic cells to restore bone immune balance; ④ Its function is mainly limited to the local bone area.	It has a strong anti-bone resorption effect and is suitable for PMOP with a high risk of fractures. After drug withdrawal, the bone turnover shows a significant “rebound”, and sequential/combined strategies are required.	(211–216)
	SERMs	Estrogen receptors (bone cells, immune cells)	① Simulate the osteoprotective effect of estrogen and inhibit pro-inflammatory factors such as TNF-α and IL-6; ② Promote the activity of regulatory immune cells and correct the immune imbalance caused by estrogen deficiency.	Suitable for postmenopausal women; Attention should be paid to adverse reactions such as thrombosis, and it is more suitable to be used as a representative drug of the “endocrine-immune-bone” triple axis.	(217)
	PTH drugs (such as Teriparatide)	Osteoblasts and their precursors; The cytokine network secreted by immune cells	① Intermittent administration promotes the proliferation and differentiation of osteoblasts; ② Increase anti-inflammatory/immunomodulatory factors such as IL-10 and IFN-γ to inhibit osteoclasts.	It is used for patients with severe osteoporosis or multiple fractures. The treatment course is limited and the price is relatively high. It often needs to be used in sequence with anti-absorption drugs.	(218, 219)
	Romosozumab (anti-Sclerostin monoclonal antibody)	Sclerostin; Wnt/β-catenin signaling Osteoblasts and immune cells	① Block Sclerostin, activate the Wnt/β-catenin pathway, significantly promote osteogenesis and inhibit osteoclasts; ② It affects the function of immune cells through Wnt signaling and improves the inflammatory state of the bone marrow.	It takes effect quickly and has a strong bone-increasing effect, but there is controversy over its cardiovascular safety. Drugs representing the “bone-immune-WNT” axis.	(220–223)
Emerging immune-targeting strategies	Anti-tnf biological agents	TNF-α and its receptors; Myeloid cells, T cells	① Inhibit TNF-mediated differentiation of osteoclastic precursors and bone resorption; ② Reduce the cytokine waterfall response driven by TNF and alleviate bone marrow inflammation.	The evidence for directly reversing bone loss in PMOP is limited; Be vigilant about the risk of infection.	(224, 225)
	IL - 6/IL - 17 biological agents	IL-6/IL-17 pathway Effector cells such as Th17	① Block the osteoclasts promoted by IL-6/IL-17 and the release of pro-inflammatory factors; ② Alleviate the excessive activation of Th17 and improve the Th17/Treg imbalance.	Most of the relevant data come from diseases such AS RA and AS. Prospective trials in the PMOP population are still lacking.	(226, 227)
	Treg/Breg enhancement strategy	Regulatory T cells, regulatory B cells	① Amplification/activation of Treg inhibits osteoclast formation; ② Increase the secretion of IL-10 by Breg and inhibit the T/B cell subsets that promote bone resorption.	At present, most of them are still in the conceptual and early experimental stages and need to be verified in estrogen deficiency models and postmenopausal populations.	(228–231)
	Immunometabolic reprogramming and MDSCs/macrophage targeting	MDSCs, Metabolic pathways of bone marrow-derived macrophages	① Regulate metabolic pathways such as glycolysis and oxidative phosphorylation to alter the immunosuppressive/pro-inflammatory phenotype of myeloid cells; ② Weaken its effects of promoting bone resorption and inflammation.	At present, animal and *in vitro* studies are the main focus, and the targeting and safety issues remain to be addressed.	(232, 233)
	Nano/genetic engineering immune intervention	Engineered probiotics, gene-modified HSCS, etc	① Precisely deliver anti-inflammatory or immunomodulatory factors to the bone marrow; ② Reshape the lineage of hematopoietic and immune cells to enhance anti-inflammatory responses.	It is still in the stage of concept exploration and early experiments, and it will take some time for it to be translated into clinical practice.	(234–236)
Comprehensive intervention of the bone marrow microenvironment	Anti-aging drugs(Senolytics)	Bone marrow senescent cells and their SASP	① Eliminate senescent cells in the bone marrow and reduce SASP factors such as TNF-α, IL-1β and IL-6; ② Alleviate chronic low-grade inflammation and restore hematopoietic and bone immune regulatory functions.	At present, most of them are preclinical/early clinical studies, and the long-term safety and the optimal dosing regimen remain unclear.	(237–242)
	Antioxidant intervention	Excessive ROS and oxidative stress pathways	① Eliminate ROS and protect the functions of BMSCs and immune cells; ② Reduce apoptosis and abnormal polarization induced by oxidative stress.	As an adjunctive treatment, it is relatively safe, but its impact on hard endpoints such as fractures still requires further evidence.	(243, 244)
	Regulation of bone marrow fat (BMAT)	Bone marrow adipocytes and their secretory factors	① Inhibit the tilt of BMSC towards the fat lineage and reduce the accumulation of BMAT; ② Reduce the pro-inflammatory mediators released by adipocytes and improve the “fat-immune-bone” coupling.	At present, the main reliance is on metabolic drugs, exercise and weight loss intervention, and clinical trials are limited.	(245)
	Optimization of the vascular-neuro-matrix axis	Bone marrow vascular endothelial cells, nerve fibers, extracellular matrix	① Promote angiogenesis and nerve regeneration, and improve the bone marrow nutrition and signaling environment; ② Regulate the migration of immune cells and bone metabolism through neuropeptides and vasogenic factors.	Most of the research focuses on animal models and regenerative medicine, while evidence in the clinical application of PMOP is still lacking.	(246, 247)
	Regulation of the gut-bone axis (probiotics, butyrate, etc.	Intestinal flora and its metabolites; Intestinal barrier function	① Promote Treg generation and inhibit inflammation through SCFAs; ② Improve the intestinal barrier, reduce the entry of LPS into the bloodstream and systemic inflammation, and indirectly optimize the bone marrow immune environment.	Some clinical studies have suggested that it is beneficial to bone metabolism, but the strains, dosages and treatment courses have not yet been standardized.	(248)
	Comprehensive intervention in lifestyle and nutrition	Exercise, diet structure (calcium, vitamin D, anti-inflammatory nutrients, etc.)	① Exercise increases bone density and improves the function of immune cells. ② Reasonable nutrition maintains bone-immune homeostasis and alleviates chronic inflammation and oxidative stress.	Safety and low cost are the basis of intervention at all levels, but the reduction in fracture risk is limited when used alone. It is advisable to combine it with drugs.	(249–251)

## 8 Future research directions and technological prospects

### 8.1 Application of multi-omics approaches in the study of bone immune microenvironment

Currently, multi-omics research on the bone marrow immune microenvironment of PMOP is still limited. Therefore, this section mainly uses bone marrow-related diseases such as AML and MM as examples to illustrate the technical pathways and analytical paradigms that can be transferred to PMOP, rather than a simple cross-disease translation of conclusions. With the development of high-throughput sequencing technology and multi-dimensional data integration analysis, multi-omics approaches have become key tools for elucidating the complexity of the bone marrow immune microenvironment. The bone immune microenvironment contains various immune cell subpopulations and their dynamic interactions. The traditional methods struggle to comprehensively reveal their spatiotemporal characteristics and balance states. In contrast, multi-omics approaches systematically depict the overall picture of cellular heterogeneity and functional states by integrating genomic, transcriptomic, epigenomic, and spatial omics data, providing strong technical support for the study of immune reprogramming.

Single-cell RNA sequencing (scRNA-seq) technology can accurately capture the gene expression profiles of immune cells at the single-cell level, revealing the identities and functional states of different cell subpopulations (252). For example, in MM, single-cell sequencing analysis has found the presence of tumor-associated special immune cell populations in the bone marrow, such as distinct NK cells and macrophages, as well as local immune cell aggregation zones. The functional states of these cells are closely related to the genomic evolution of tumor cells, suggesting that dynamic changes in immune cells are key factors in tumor progression (253). Additionally, in the study of AML, single-cell sequencing combined with multi-omics analyses have revealed differences in gene signatures among different immune cell subpopulations. Based on this, a prognostic prediction model was constructed using immune-related genes. This model shows significant differences in patient prognosis between immune-rich and immune-deficient subpopulations (254). These studies indicate that single-cell sequencing can not only finely characterize the diversity of immune cells but also dynamically track their functional transitions during disease progression.

Although single-cell sequencing can reveal cell types and states, its lack of spatial location information limits the understanding of intercellular interactions. Spatial omics technology addresses this limitation by revealing the spatial distribution of cell populations and their signaling communication networks through the combination of gene expression information with the spatial locations of tissues. In the study of AML patients receiving immune therapy, the combination of single-cell RNA sequencing and spatial transcriptomics technology precisely analyzed the spatial distribution of immune cells in the bone marrow and their proximity to leukemia cells. The study found that the local enrichment of immune cells around tumor cells is closely related to treatment response. Additionally, ligand-receptor analysis revealed potential changes in signaling pathways after immune therapy (255). In the bone marrow breakthrough lesions of multiple myeloma, spatial multi-omics revealed the complex spatial structures and diverse interactions between tumor cells and immune cells, suggesting that breakthrough lesions are key sites for the diversification of tumor-immune cell interactions. Furthermore, the combination of spatial omics and single-cell sequencing data helps establish intercellular communication network models, providing in-depth analysis of signaling transmission and immune regulation mechanisms in the bone marrow immune microenvironment (256, 257).

**Figure 7:**
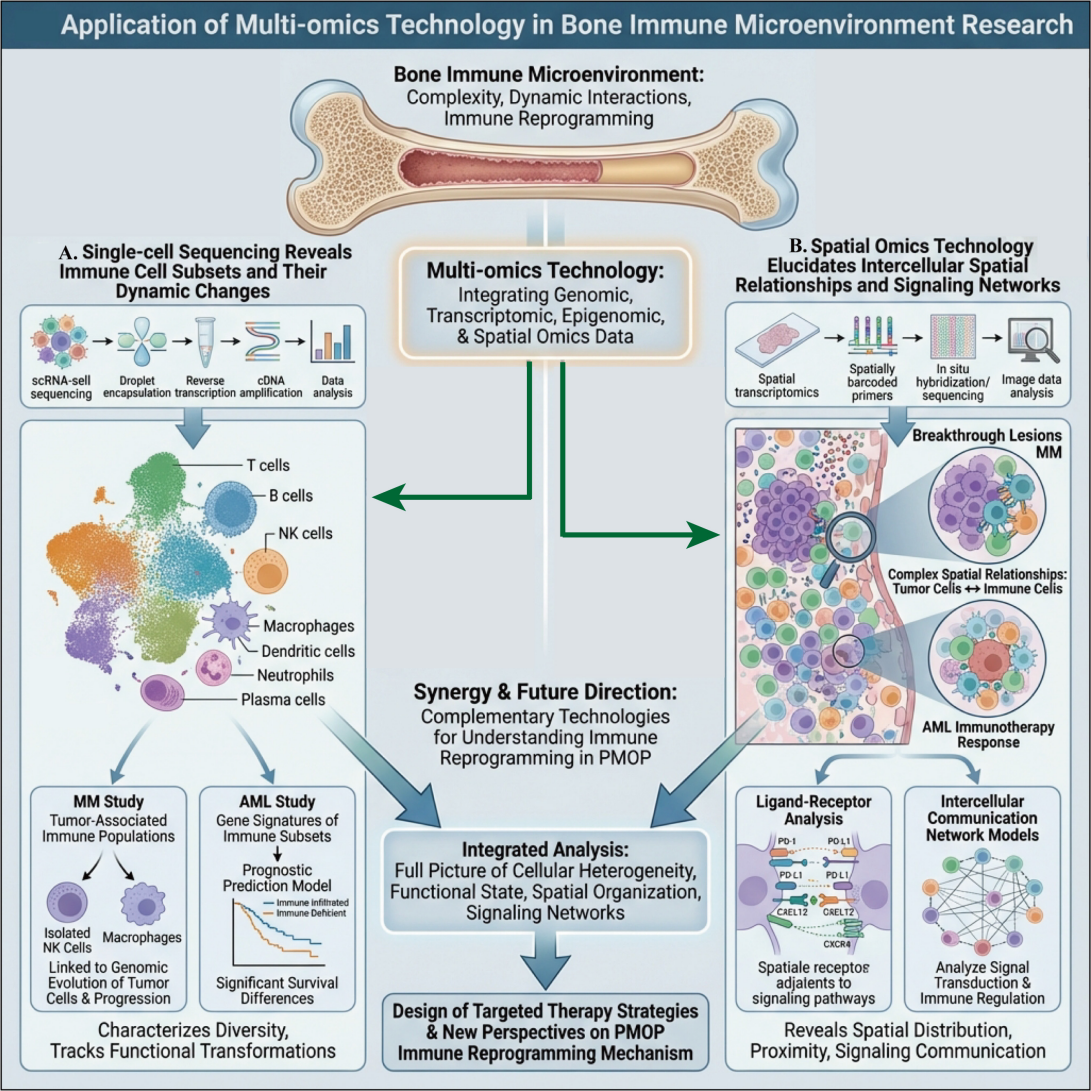
Schematic diagram of the application of multi-omics technology in the study of the bone immune microenvironment in PMOP. This figure emphasizes the systematic characterization of the heterogeneity, functional status, and key signaling networks of bone marrow immune cells by integrating multi-omics data, such as genome, transcriptome, epigenome, and spatial omics. Panel A: Single-cell-related technologies, such as single-cell RNA sequencing, are used to finely analyze immune subpopulations, including T and B cells and macrophages, as well as their reprogramming characteristics, thereby constructing a bone immune cell atlas. Panel B: Spatial omics links cell types with their spatial locations within tissue sections, analyzing the proximity relationships, and ligand-receptor interactions between immune cells and bone metabolism-related cells in the bone marrow. Together, these comprehensive multi-omics analyses help to construct an overall picture of the PMOP bone immune microenvironment. This provides a basis for elucidating immune reprogramming mechanisms and designing targeted intervention strategies.

In summary, multi-omics technology reveals the heterogeneity and dynamic changes of immune cells through single-cell sequencing, while spatial omics technology complements this by providing spatial relationships and signaling network information between cells. The two approaches complement each other and jointly promote the in-depth development of research on the bone marrow immune microenvironment. Currently, single-cell and spatial omics research on the bone marrow immune microenvironment of PMOP is still in its early stages. However, the research paradigms mentioned above in diseases such as AML and MM have already provided a reference technical route and analytical framework for constructing the PMOP bone immune map. In the future, applying similar strategies in ovarian removal animal models and postmenopausal female bone marrow samples is expected to finely characterize the cellular lineages and signaling networks of “trained inflammatory bone marrow,” laying the foundation for immune stratification and individualized intervention (**Figure 7**).

### 8.2 The combination of animal models and clinical cohorts to construct a bone immune microenvironment map

In the field of PMOP, there are still limited animal models and clinical cohort studies that truly focus on “bone marrow immune reprogramming” as the core endpoint. Therefore, this section mainly refers to studies on bone tumors, AML, MM, and other related diseases as methodological and conceptual references, rather than directly extrapolating their pathological conclusions. Constructing a detailed bone immune microenvironment map is a key step in understanding the pathogenesis of PMOP and screening potential therapeutic targets. Currently, research strategies that combine animal models and clinical cohort data provide strong support for this field. First, using transgenic and aging animal models is an effective approach to simulate immune reprogramming in PMOP. Traditional osteoporosis animal models often use specific pathogen-free (SPF) mice; however, the immune systems of SPF mice are relatively immature compared to those of adult humans, making it difficult to fully replicate the immune maturation state. Recent studies suggest using “dirty mouse” models—mice exposed to various microorganisms that develop a more human-like immune system—allowing for a more realistic reflection of the interactions between the immune system and bone tissue, especially regarding immune regulatory mechanisms in osteoporosis and aging-related diseases (258). In addition to “dirty mouse” models, transgenic mice generated through gene knockout technology have been utilized in studies targeting the bone immune microenvironment. For example, in mice with the Skp2 gene knocked out, significant increases in immune cell infiltration were observed in their bone tumor microenvironment. This finding indicates a close relationship between gene regulation and immune rejection, which has implications for the immune regulation of osteoporosis (259). Aging animal models are equally important. For instance, Zmpste24-deficient progeroid mice exhibit delayed fracture healing accompanied by immune system disturbances, such as reduced lymphocyte generation in the bone marrow and increased activation of myeloid cells. These changes demonstrate the impact of the immune microenvironment on bone repair (260). Through these models, the polarization state of immune cells—especially macrophages of the M1 and M2 types—can be dynamically monitored. This monitoring helps regulate bone metabolism and the remodeling process of the bone immune microenvironment, thereby revealing the molecular mechanisms underlying immune reprogramming (261).

Secondly, clinical cohort studies provide practical evidence validating the correlation between immune markers discovered in animal models and the severity of osteoporosis. For example, in analyzing the bone marrow immune microenvironment of patients with AML and MM, researchers found that specific immune gene expression and the abundance of immune cell subpopulations (such as CD8+ T cells, regulatory T cells, and myeloid-derived suppressor cells) closely correlate with disease progression and prognosis (262, 263). Similarly, in patients with osteosarcoma, prognosis models based on immune-related genes indicated that the degree of immune infiltration significantly associates with patient survival rates, highlighting the importance of the immune microenvironment in bone tumors and osteoporosis (264). Furthermore, single-cell RNA sequencing technology combined with spatial transcriptomics provides technical support for constructing the bone immune microenvironment map. Through high-resolution analysis of immune cells in bone tissue and bone marrow, the complex interactions between immune cells and bone metabolic cells—such as MSCs, osteoblasts, and osteoclasts—have been revealed (265). This technological approach helps identify key cell subpopulations and signaling pathways related to immune reprogramming, providing a molecular basis for validating immune markers in clinical cohorts. Finally, combining animal models with clinical cohort data establishes a comprehensive bone immune microenvironment map. For example, the single-cell immunogenomics map of multiple myeloma reveals the heterogeneity of bone marrow immune cells and their association with clinical prognosis, offering important references for the immune regulation of osteoporosis (266, 267). In bone metastatic tumor models, the role of immunosuppressive cells—such as myeloid-derived suppressor cells—in the bone immune microenvironment has also been observed. This observation suggests that immune reprogramming is not limited to osteoporosis but also involves regulation of the bone tumor microenvironment (268, 269).

Overall, the field of PMOP can fully utilize an integrated methodological approach combining transgenic, aging, and ovariectomized animal models with single-cell and spatial omics technologies, alongside clinical bone marrow cohorts, to construct a bone immune microenvironment map specifically for PMOP. This approach not only systematically characterizes the lineage bias of HSCs and MSCs, the reprogramming of innate and adaptive immune cells, and the remodeling of cytokine networks, but also provides a solid translational basis for precise immune stratification and targeted interventions.

### 8.3 Precision stratification and individualized intervention strategies

With the in-depth understanding of the role of the bone marrow immune microenvironment in the pathogenesis of PMOP, precision stratification and individualized intervention strategies have become important directions in current research and clinical applications. Classifying patients based on the characteristics of the immune microenvironment helps reveal the differences in immune status among different patients, thereby guiding the formulation of more precise treatment plans (270). By integrating single-cell and bulk transcriptome data, studies have found that PMOP patients can be divided into two molecular subtypes based on immune-related differentially expressed genes (IR-DEGs). Subtype 1 shows a higher proportion of M1 macrophage infiltration, indicating a more significant immune activation state (271). In addition, key immune genes such as JUN are significantly overexpressed in M1 macrophages, while HMOX1 is mainly elevated in endothelial cells and M2 macrophages. These immune cells and their interactions constitute a complex network of the PMOP bone immune microenvironment. The diagnostic models constructed based on these immune markers show high accuracy; they provide a powerful tool for early clinical diagnosis and patient classification (272). In terms of individualized treatment, precise immune typing can not only predict the immune status of the disease but also guide the development of innovative drugs targeting immune reprogramming (273). Furthermore, current research indicates that gut microbiota and their metabolites play a key regulatory role in the bone immune microenvironment. Short-chain fatty acids and tryptophan derivatives influence bone metabolism by regulating immune signaling pathways, inflammatory responses, and endocrine networks (274). These effects are concentration-dependent and vary according to specific physiological contexts; they can promote bone formation but may also exacerbate bone resorption. This suggests that precise dosage control and selection of appropriate patient populations are necessary when developing microbiota-targeted therapeutic strategies. Based on this, microbial intervention methods such as probiotics, prebiotics, and fecal microbiota transplantation are being actively explored as emerging directions for the treatment of osteoporosis, especially for elderly patients with impaired immune function. In such cases, personalized risk assessment and safety monitoring of potential adverse effects are particularly important (274).

**Figure 8:**
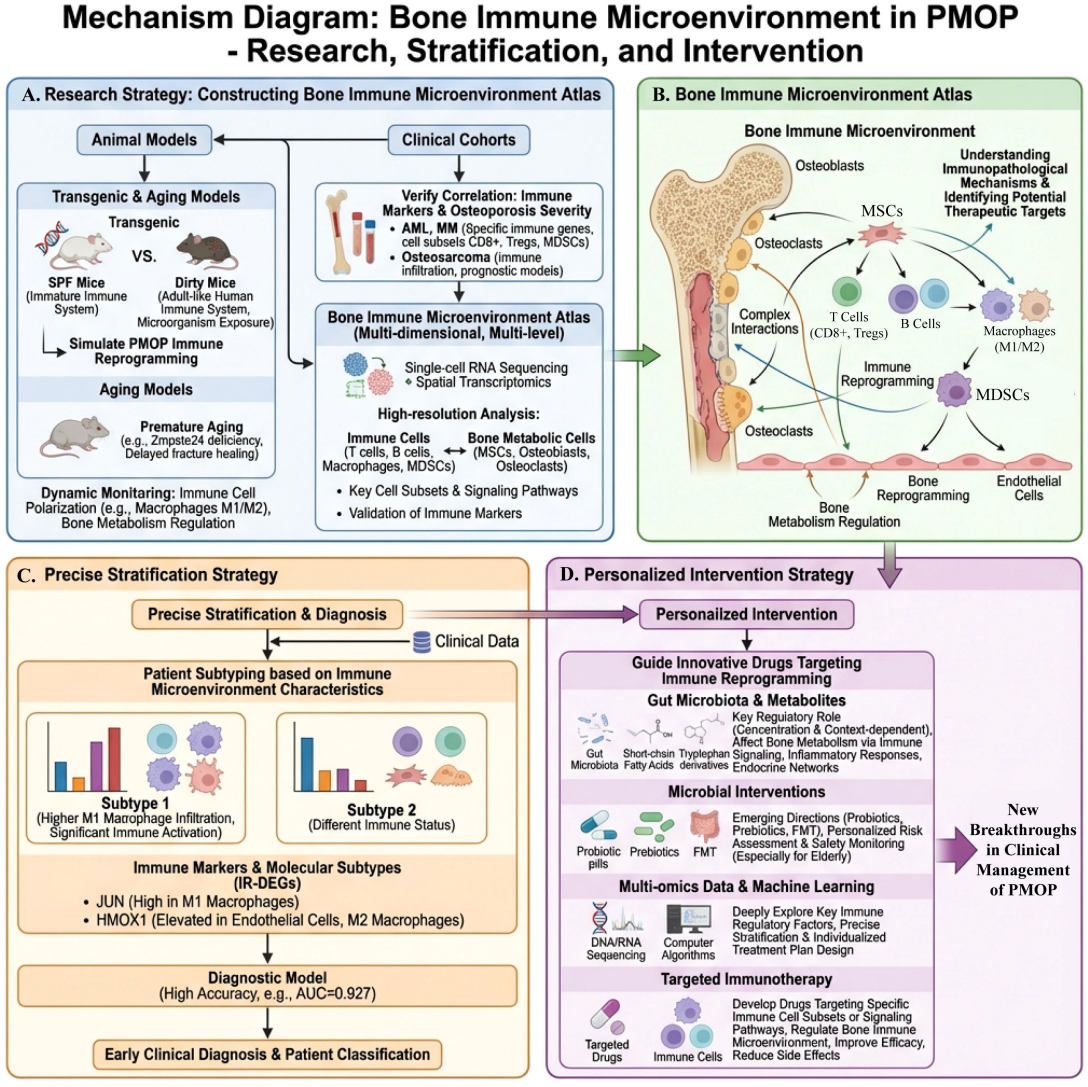
Research on the bone immune microenvironment of PMOP – stratification – integrated intervention framework schematic diagram. This figure summarizes the overall idea from mechanism research to patient stratification and then to individualized intervention in PMOP, which mainly includes four modules. **(A)** Research strategy module: This module combines animal models and clinical cohorts, using single-cell and spatial omics along with other methods to map the multidimensional bone immune microenvironment. **(B)** Bone immune microenvironment map module: This module displays the interactions and immune reprogramming of osteoblasts, osteoclasts, MSCs, T and B cells, macrophages, HUVECs, and other relevant cell types in the bone marrow niche, providing a basis for mechanism analysis and target screening. **(C)** Precision stratification module: This module constructs risk evaluation and diagnostic and stratification models based on the characteristics of the bone immune microenvironment and immune-related molecules, achieving early recognition and subtype classification of PMOP patients. **(D)** Individualized intervention module: This module explores comprehensive treatment pathways targeting different immune-bone phenotypes through strategies such as microecological regulation, molecular targeted drugs, and specific immune pathway interventions.

In the future, the integration of multi-omics data and machine learning algorithms will enable a deeper exploration of key immune regulatory factors in the bone marrow immune microenvironment, facilitating precise patient stratification and the design of individualized treatment plans for PMOP patients. Additionally, developing drugs that target specific immune cell subpopulations or signaling pathways can effectively modulate immune responses within the bone marrow microenvironment, promote bone remodeling, enhance therapeutic effects, and reduce side effects, thereby bringing new breakthroughs to the clinical treatment of PMOP. In summary, the integration of precise immune typing and personalized immune regulation strategies not only enriches the understanding of the pathological mechanisms of osteoporosis but also provides practical, novel therapeutic strategies for clinical practice (**Figure 8**).

## 9 Conclusion

The pathological concept of PMOP is undergoing a fundamental transformation: it is not only a traditional “imbalance between osteoblasts and osteoclasts,” but also a result of the reprogramming of multicellular lineages and signaling networks in the bone marrow immune microenvironment. This means that PMOP should be regarded as an integrated imbalance among bone, immunity, and metabolism, rather than merely a disease of reduced bone mass. At the mechanistic level, this review emphasizes the central role of “two core pathways and two types of key cell populations.” On one hand, the bone-immune coupling signaling axis represented by RANK/RANKL/OPG and Wnt/β-catenin forms the molecular “main line” connecting bone remodeling and immune inflammation. On the other hand, key immune cell populations represented by M1 and M2 macrophages, Th17 and Treg cells, and regulatory B cells and B cell subsets (Breg and ABCs) shape the inflammation–anti-inflammation balance and influence the osteoclast/osteoblast bias, determining whether the bone marrow immune microenvironment evolves towards bone loss or restoration of bone homeostasis.

From a therapeutic perspective, existing anti-osteoporosis drugs (such as bisphosphonates, estrogens and their analogs, RANKL inhibitors, etc.) are clinically targeted at bone but have inadvertently reshaped the bone immune microenvironment, providing us with a window to reinterpret the mechanisms of action of existing drugs. In the future, more forward-looking immune regulation strategies—including biologic agents targeting specific cytokines or receptors, cell therapies targeting specific immune cell subsets, and metabolic reprogramming aimed at immune cells and bone cells—are expected to achieve a closed-loop intervention of “from bone to immunity, and back to bone,” bringing truly stratified treatments based on patient immune and bone profiles and individualized plans to PMOP patients.

Methodologically and in terms of future directions, the deep integration of multi-omics (genomics, transcriptomics, proteomics, metabolomics) with single-cell omics and spatial omics, along with integration with animal models such as ovariectomy and real-world clinical cohorts, will be key approaches for constructing the PMOP bone immune map. Through these approaches, researchers aim to finely characterize the bone marrow immune phenotypes and molecular subtypes of different patients, identify high-risk populations and specific treatment-sensitive subsets, and accelerate the discovery of new targets and strategies with the assistance of artificial intelligence and big data analysis. Overall, bone immunology provides sharper and more translatable theoretical support for the mechanistic elucidation and therapeutic innovation of PMOP, and is expected to promote the field from a singular focus on anti-bone resorption and anabolic treatment to a new stage of precise intervention targeting both immunity and bone.

## References

[1] Sun S Xiu C Chai L Chen X Zhang L Liu Q . HDAC inhibitor quisinostat prevents estrogen deficiency-induced bone loss by suppressing bone resorption and promoting bone formation in mice. Eur J Pharmacol. (2022) 927:175073. doi: 10.1016/j.ejphar.2022.175073, PMID: 35636521

[2] Liang H Chen S Shi M Xu J Zhao C Yang B . Global epidemiology and burden of osteoporosis among postmenopausal women: insights from the Global Burden of Disease Study 2021. NPJ Aging. (2025) 11:78. doi: 10.1038/s41514-025-00269-2, PMID: 40890217 PMC12402057

[3] Satpathy S Patra A Ahirwar B . Experimental techniques for screening of antiosteoporotic activity in postmenopausal osteoporosis. J Complement Integr Med. (2015) 12:251–66. doi: 10.1515/jcim-2015-0034, PMID: 26215536

[4] Fischer V Haffner-Luntzer M . Interaction between bone and immune cells: Implications for postmenopausal osteoporosis. Semin Cell Dev Biol. (2022) 123:14–21. doi: 10.1016/j.semcdb.2021.05.014, PMID: 34024716

[5] Andreev D Kachler K Schett G Bozec A . Rheumatoid arthritis and osteoimmunology: The adverse impact of a deregulated immune system on bone metabolism. Bone. (2022) 162:116468. doi: 10.1016/j.bone.2022.116468, PMID: 35688359

[6] Cai L Lv Y Yan Q Guo W . Cytokines: The links between bone and the immune system. Injury. (2024) 55:111203. doi: 10.1016/j.injury.2023.111203, PMID: 38043143

[7] Li M Sun H Liu L Ning Y Cao Y Lu B . Pro-inflammatory immune microenvironment and Thrombospondin-1-positive monocytes as drivers of osteoclastogenesis in postmenopausal osteoporosis. J Bone Miner Res. (2025) 40:1061–76. doi: 10.1093/jbmr/zjaf083, PMID: 40515615

[8] Ji J Gu Z Li N Dong X Wang X Yao Q . Gut microbiota alterations in postmenopausal women with osteoporosis and osteopenia from Shanghai, China. PeerJ. (2024) 12:e17416. doi: 10.7717/peerj.17416, PMID: 38832037 PMC11146318

[9] Bhardwaj A Sapra L Madan D Ahuja V Sharma HP Velpandian T . Gut-resident regulatory T cells (GTregs) play a pivotal role in maintaining bone health under postmenopausal osteoporotic conditions. J Leukoc Biol. (2025) 117. doi: 10.1093/jleuko/qiaf008, PMID: 39829025

[10] Li H Deng W Qin Q Lin Y Liu T Mo G . Isoimperatorin attenuates bone loss by inhibiting the binding of RANKL to RANK. Biochem Pharmacol. (2023) 211:115502. doi: 10.1016/j.bcp.2023.115502, PMID: 36921635

[11] Xu Y Murphy AJ Fleetwood AJ . Hematopoietic progenitors and the bone marrow niche shape the inflammatory response and contribute to chronic disease. Int J Mol Sci. (2022) 23. doi: 10.3390/ijms23042234, PMID: 35216355 PMC8879433

[12] Li X Wang Z Chen Y Yang Y Shao H Feng X . Regulation of monocyte polarization through nuclear factor Kappa B/inhibitor of Kappa B Alpha pathway by Cuscuta chinensis Lam. In postmenopausal osteoporosis. J Ethnopharmacol. (2025) 346:119710. doi: 10.1016/j.jep.2025.119710, PMID: 40154899

[13] Liu J Li F Ouyang Y Su Z Chen D Liang Z . Naringin-induced M2 macrophage polarization facilitates osteogenesis of BMSCs and improves cranial bone defect healing in rat. Arch Biochem Biophys. (2024) 753:109890. doi: 10.1016/j.abb.2024.109890, PMID: 38246327

[14] Mcclung MR . Postmenopausal osteoporosis. Menopause. (2025) 32:359–60. doi: 10.1097/GME.0000000000002514, PMID: 40127108

[15] Chen X Yuan Y Zhou X Deng H Zhang J Duan S . The role of taurine in bone metabolism. iScience. (2025) 28:113257. doi: 10.1016/j.isci.2025.113257, PMID: 40894901 PMC12391252

[16] Xu C Yang J Kosters A Babcock BR Qiu P Ghosn EEB . Comprehensive multi-omics single-cell data integration reveals greater heterogeneity in the human immune system. iScience. (2022) 25:105123. doi: 10.1016/j.isci.2022.105123, PMID: 36185375 PMC9523353

[17] Huidrom S Beg MA Masood T . Post-menopausal osteoporosis and probiotics. Curr Drug Targets. (2021) 22:816–22. doi: 10.2174/18735592MTEwrOTUbx, PMID: 33109043

[18] Chang B Quan Q Li Y Qiu H Peng J Gu Y . Treatment of osteoporosis, with a focus on 2 monoclonal antibodies. Med Sci Monit. (2018) 24:8758–66. doi: 10.12659/MSM.912309, PMID: 30508820 PMC6289028

[19] Wong SK Mohamad NV Ibrahim N Chin KY Shuid AN Ima-Nirwana S . The molecular mechanism of vitamin E as a bone-protecting agent: A review on current evidence. Int J Mol Sci. (2019) 20. doi: 10.3390/ijms20061453, PMID: 30909398 PMC6471965

[20] Lee JM Kim MJ Lee SJ Kim BG Choi JY Lee SM . PDK2 deficiency prevents ovariectomy-induced bone loss in mice by regulating the RANKL-NFATc1 pathway during osteoclastogenesis. J Bone Miner Res. (2021) 36:553–66. doi: 10.1002/jbmr.4202, PMID: 33125772

[21] Cheng CH Chen LR Chen KH . Osteoporosis due to hormone imbalance: an overview of the effects of estrogen deficiency and glucocorticoid overuse on bone turnover. Int J Mol Sci. (2022) 23. doi: 10.3390/ijms23031376, PMID: 35163300 PMC8836058

[22] Yang C Yang P Liu P Wang H Ke E Li K . Targeting Filamin A alleviates ovariectomy-induced bone loss in mice via the WNT/β-catenin signaling pathway. Cell Signal. (2022) 90:110191. doi: 10.1016/j.cellsig.2021.110191, PMID: 34774991

[23] Ryoo GH Moon YJ Choi S Bae EJ Ryu JH Park BH . Tussilagone promotes osteoclast apoptosis and prevents estrogen deficiency-induced osteoporosis in mice. Biochem Biophys Res Commun. (2020) 531:508–14. doi: 10.1016/j.bbrc.2020.07.083, PMID: 32807498

[24] Terauchi M . Role of the immune system in the pathophysiology of postmenopausal osteoporosis. Nihon Rinsho. (2011) 69:1215–9. 21774360

[25] Sasso G Cerri PS Sasso-Cerri E Simões MJ Gil CD Florencio-Silva R . Possible role of annexin A1/FPR2 pathway in COX2/NLRP3 inflammasome regulation in alveolar bone cells of estrogen-deficient female rats with diabetes mellitus. J Periodontol. (2024) 95:749–63. doi: 10.1002/JPER.23-0530, PMID: 37987258

[26] Terashima A Takayanagi H . The role of bone cells in immune regulation during the course of infection. Semin Immunopathol. (2019) 41:619–26. doi: 10.1007/s00281-019-00755-2, PMID: 31552472

[27] Xu H Li Y Gao Y . The role of immune cells settled in the bone marrow on adult hematopoietic stem cells. Cell Mol Life Sci. (2024) 81:420. doi: 10.1007/s00018-024-05445-3, PMID: 39367881 PMC11456083

[28] Ikeogu NM Edechi CA Akaluka GN Feiz-Barazandeh A Uzonna JE . Isolation and preparation of bone marrow-derived immune cells for metabolic analysis. Methods Mol Biol. (2020) 2184:273–80. 10.1007/978-1-0716-0802-9_19 32808232

[29] Kaul K Benej M Mishra S Ahirwar DK Yadav M Stanford KI . Slit2-mediated metabolic reprogramming in bone marrow-derived macrophages enhances antitumor immunity. Front Immunol. (2021) 12:753477. doi: 10.3389/fimmu.2021.753477, PMID: 34777365 PMC8581492

[30] Udagawa N Koide M Nakamura M Nakamichi Y Yamashita T Uehara S . Osteoclast differentiation by RANKL and OPG signaling pathways. J Bone Miner Metab. (2021) 39:19–26. doi: 10.1007/s00774-020-01162-6, PMID: 33079279

[31] Kobayashi Y Udagawa N Takahashi N . Action of RANKL and OPG for osteoclastogenesis. Crit Rev Eukaryot Gene Expr. (2009) 19:61–72. doi: 10.1615/CritRevEukarGeneExpr.v19.i1.30, PMID: 19191757

[32] Hu K Song M Song T Jia X Song Y . Osteoimmunology in osteoarthritis: unraveling the interplay of immunity, inflammation, and joint degeneration. J Inflammation Res. (2025) 18:4121–42. doi: 10.2147/JIR.S514002, PMID: 40125089 PMC11930281

[33] Ma H Gao L Chang R Zhai L Zhao Y . Crosstalk between macrophages and immunometabolism and their potential roles in tissue repair and regeneration. Heliyon. (2024) 10:e38018. doi: 10.1016/j.heliyon.2024.e38018, PMID: 39381218 PMC11458987

[34] Dabaliz A Al Hakawati MN Alrashdan N Alrashdan S Bakir M Mohammad KS . Adipocyte-tumor interactions in the bone marrow niche: implications for metastasis and therapy. Int J Mol Sci. (2025) 26. doi: 10.3390/ijms26199781, PMID: 41097047 PMC12525404

[35] Aaron N Costa S Rosen CJ Qiang L . The implications of bone marrow adipose tissue on inflammaging. Front Endocrinol (Lausanne). (2022) 13:853765. doi: 10.3389/fendo.2022.853765, PMID: 35360075 PMC8962663

[36] Pu H Ding L Jiang P Li G Wang K Jiang J . New insight into bone immunity in marrow cavity and cancellous bone microenvironments and their regulation. Biomedicines. (2025) 13. doi: 10.3390/biomedicines13102426, PMID: 41153709 PMC12561077

[37] Intemann J De Gorter DJJ Naylor AJ Dankbar B Wehmeyer C . Importance of osteocyte-mediated regulation of bone remodelling in inflammatory bone disease. Swiss Med Wkly. (2020) 150:w20187. doi: 10.4414/smw.2020.20187, PMID: 32031236

[38] Yang N Liu Y . The role of the immune microenvironment in bone regeneration. Int J Med Sci. (2021) 18:3697–707. doi: 10.7150/ijms.61080, PMID: 34790042 PMC8579305

[39] Riether C Schürch CM Ochsenbein AF . Regulation of hematopoietic and leukemic stem cells by the immune system. Cell Death Differ. (2015) 22:187–98. doi: 10.1038/cdd.2014.89, PMID: 24992931 PMC4291501

[40] Xu Y Yan H Zhang X Zhuo J Han Y Zhang H . Roles of altered macrophages and cytokines: implications for pathological mechanisms of postmenopausal osteoporosis, rheumatoid arthritis, and alzheimer’s disease. Front Endocrinol (Lausanne). (2022) 13:876269. doi: 10.3389/fendo.2022.876269, PMID: 35757427 PMC9226340

[41] Gong Y Hao D Zhang Y Tu Y He B Yan L . Molecular subtype classification of postmenopausal osteoporosis and immune infiltration microenvironment based on bioinformatics analysis of osteoclast-regulatory genes. Biomedicines. (2023) 11. doi: 10.3390/biomedicines11102701, PMID: 37893075 PMC10604900

[42] Murayama M Chow SK Lee ML Young B Ergul YS Shinohara I . The interactions of macrophages, lymphocytes, and mesenchymal stem cells during bone regeneration. Bone Joint Res. (2024) 13:462–73. doi: 10.1302/2046-3758.139.BJR-2024-0122.R1, PMID: 39237112 PMC11377107

[43] Kamrani S Naseramini R Khani P Razavi ZS Afkhami H Atashzar MR . Mesenchymal stromal cells in bone marrow niche of patients with multiple myeloma: a double-edged sword. Cancer Cell Int. (2025) 25:117. doi: 10.1186/s12935-025-03741-x, PMID: 40140850 PMC11948648

[44] He C He P Ou Y Tang X Wei H Xu Y . Rectifying the crosstalk between the skeletal and immune systems improves osteoporosis treatment by core-shell nanocapsules. ACS Nano. (2025) 19:5549–67. doi: 10.1021/acsnano.4c14728, PMID: 39879106

[45] Xu Z Wu L Tang Y Xi K Tang J Xu Y . Spatiotemporal regulation of the bone immune microenvironment via dam-like biphasic bionic periosteum for bone regeneration. Adv Healthc Mater. (2023) 12:e2201661. doi: 10.1002/adhm.202201661, PMID: 36189833 PMC11469314

[46] He J Chen G Liu M Xu Z Chen H Yang L . Scaffold strategies for modulating immune microenvironment during bone regeneration. Mater Sci Eng C Mater Biol Appl. (2020) 108:110411. doi: 10.1016/j.msec.2019.110411, PMID: 31923946

[47] Li M Chu X Wang D Jian L Liu L Yao M . Tuning the surface potential to reprogram immune microenvironment for bone regeneration. Biomaterials. (2022) 282:121408. doi: 10.1016/j.biomaterials.2022.121408, PMID: 35189460

[48] Elias HK Bryder D Park CY . Molecular mechanisms underlying lineage bias in aging hematopoiesis. Semin Hematol. (2017) 54:4–11. doi: 10.1053/j.seminhematol.2016.11.002, PMID: 28088987

[49] Woods K Guezguez B . Dynamic changes of the bone marrow niche: mesenchymal stromal cells and their progeny during aging and leukemia. Front Cell Dev Biol. (2021) 9:714716. doi: 10.3389/fcell.2021.714716, PMID: 34447754 PMC8383146

[50] Fan Y Elkhalek M Zhang Y Liu L Tian Q Chueakula N . Bone marrow adipocytes: key players in vascular niches, aging, and disease. Front Cell Dev Biol. (2025) 13:1633801. doi: 10.3389/fcell.2025.1633801, PMID: 40852589 PMC12367753

[51] Singh P Kacena MA Orschell CM Pelus LM . Aging-related reduced expression of CXCR4 on bone marrow mesenchymal stromal cells contributes to hematopoietic stem and progenitor cell defects. Stem Cell Rev Rep. (2020) 16:684–92. doi: 10.1007/s12015-020-09974-9, PMID: 32418119 PMC7395885

[52] Nelson TA Tommasini S Fretz JA . Deletion of the transcription factor EBF1 in perivascular stroma disrupts skeletal homeostasis and precipitates premature aging of the marrow microenvironment. Bone. (2024) 187:117198. doi: 10.1016/j.bone.2024.117198, PMID: 39002837 PMC11410106

[53] Fichtel P von Bonin M Kuhnert R Möbus K Bornhäuser M Wobus M . Mesenchymal stromal cell-derived extracellular vesicles modulate hematopoietic stem and progenitor cell viability and the expression of cell cycle regulators in an age-dependent manner. Front Bioeng Biotechnol. (2022) 10:892661. doi: 10.3389/fbioe.2022.892661, PMID: 35721867 PMC9198480

[54] Li Z Gao K Wang M Liang S Li D Zhang P . Naringin inhibits the osteoblast-osteoclast pyroptosis cascade reaction mediated by accumulated bone marrow adipose tissue in the treatment of postmenopausal osteoporosis. J Orthop Translat. (2025) 55:323–38. doi: 10.1016/j.jot.2025.09.004, PMID: 41104349 PMC12522718

[55] Bordukalo-Nikšić T Kufner V Vukičević S . The role of BMPs in the regulation of osteoclasts resorption and bone remodeling: from experimental models to clinical applications. Front Immunol. (2022) 13:869422. doi: 10.3389/fimmu.2022.869422, PMID: 35558080 PMC9086899

[56] Daponte V Henke K Drissi H . Current perspectives on the multiple roles of osteoclasts: Mechanisms of osteoclast-osteoblast communication and potential clinical implications. Elife. (2024) 13. doi: 10.7554/eLife.95083, PMID: 38591777 PMC11003748

[57] Hu Y Tang XX He HY . Gene expression during induced differentiation of sheep bone marrow mesenchymal stem cells into osteoblasts. Genet Mol Res. (2013) 12:6527–34. doi: 10.4238/2013.December.11.4, PMID: 24390999

[58] Sila-Asna M Bunyaratvej A Maeda S Kitaguchi H Bunyaratavej N . Osteoblast differentiation and bone formation gene expression in strontium-inducing bone marrow mesenchymal stem cell. Kobe J Med Sci. (2007) 53:25–35. 17579299

[59] Ganesh T Laughrey LE Niroobakhsh M Lara-Castillo N . Multiscale finite element modeling of mechanical strains and fluid flow in osteocyte lacunocanalicular system. Bone. (2020) 137:115328. doi: 10.1016/j.bone.2020.115328, PMID: 32201360 PMC7354216

[60] Riquelme MA Cardenas ER Xu H Jiang JX . The role of connexin channels in the response of mechanical loading and unloading of bone. Int J Mol Sci. (2020) 21. doi: 10.3390/ijms21031146, PMID: 32050469 PMC7038207

[61] Pathak JL Bravenboer N Klein-Nulend J . The osteocyte as the new discovery of therapeutic options in rare bone diseases. Front Endocrinol (Lausanne). (2020) 11:405. doi: 10.3389/fendo.2020.00405, PMID: 32733380 PMC7360678

[62] Rossi F Tortora C Punzo F Bellini G Argenziano M Di Paola A . The endocannabinoid/endovanilloid system in bone: from osteoporosis to osteosarcoma. Int J Mol Sci. (2019) 20. doi: 10.3390/ijms20081919, PMID: 31003519 PMC6514542

[63] Hou X Tian F . STAT3-mediated osteogenesis and osteoclastogenesis in osteoporosis. Cell Commun Signal. (2022) 20:112. doi: 10.1186/s12964-022-00924-1, PMID: 35879773 PMC9310501

[64] Wang M Cao Z Hu W Dong Z Wang Y Ma C . The role of the immune microenvironment on osteoblast differentiation. Immunol Invest. (2025) 54:962–1011. doi: 10.1080/08820139.2025.2527246, PMID: 40657659

[65] Choi YY Jin SC Song M Yi S Park J Baek HK . Cervus elaphus sibiricus (deer antler) extract alleviates osteoporosis via dual modulation of osteoblast and osteoclast activity in ovariectomy-induced mice on network pharmacology. J Ethnopharmacol. (2026) 355:120669. doi: 10.1016/j.jep.2025.120669, PMID: 41033422

[66] Bonewald LF Johnson ML . Osteocytes, mechanosensing and Wnt signaling. Bone. (2008) 42:606–15. doi: 10.1016/j.bone.2007.12.224, PMID: 18280232 PMC2349095

[67] Holliday LS Patel SS Rody WJ . RANKL and RANK in extracellular vesicles: surprising new players in bone remodeling. Extracell Vesicles Circ Nucl Acids. (2021) 2:18–28. doi: 10.20517/evcna.2020.02, PMID: 33982033 PMC8112638

[68] Zhao W Qian J Li J Su T Deng X Fu Y . From death to birth: how osteocyte death promotes osteoclast formation. Front Immunol. (2025) 16:1551542. doi: 10.3389/fimmu.2025.1551542, PMID: 40165960 PMC11955613

[69] Pan T Liu F Hao X Wang S Wasi M Song JH . BIGH3 mediates apoptosis and gap junction failure in osteocytes during renal cell carcinoma bone metastasis progression. Cancer Lett. (2024) 596:217009. doi: 10.1016/j.canlet.2024.217009, PMID: 38849015 PMC11964150

[70] Ford S Tiedemann K Shapiro R Komarova SV Jähn-Rickert K Zimmermann EA . Fluorescent mapping of osteocyte-driven bone formation at pre-osteocyte and mature osteocyte lacunae. Acta Biomater. (2025) 207:444–55. doi: 10.1016/j.actbio.2025.09.040, PMID: 41027524

[71] Li J Sakisaka Y Nemoto E Maruyama K Suzuki S Xiong K . Cementocyte-derived extracellular vesicles regulate osteoclastogenesis and osteoblastogenesis. J Dent Sci. (2024) 19:2236–46. doi: 10.1016/j.jds.2024.02.025, PMID: 39347082 PMC11437308

[72] Cui Y Lv B Li Z Ma C Gui Z Geng Y . Bone-Targeted Biomimetic Nanogels Re-Establish Osteoblast/Osteoclast Balance to Treat Postmenopausal Osteoporosis. Small. (2024) 20:e2303494. doi: 10.1002/smll.202303494, PMID: 37794621

[73] Lu XL Huo B Chiang V Guo XE . Osteocytic network is more responsive in calcium signaling than osteoblastic network under fluid flow. J Bone Miner Res. (2012) 27:563–74. doi: 10.1002/jbmr.1474, PMID: 22113822 PMC3343217

[74] Polineni S Resulaj M Faje AT Meenaghan E Bredella MA Bouxsein M . Red and White Blood Cell Counts Are Associated With Bone Marrow Adipose Tissue, Bone Mineral Density, and Bone Microarchitecture in Premenopausal Women. J Bone Miner Res. (2020) 35:1031–9. doi: 10.1002/jbmr.3986, PMID: 32078187 PMC7881438

[75] Niu H Zhou M Xu X Xu X . Bone Marrow Adipose Tissue as a Critical Regulator of Postmenopausal Osteoporosis - A Concise Review. Clin Interv Aging. (2024) 19:1259–72. doi: 10.2147/cia.S466446, PMID: 39011312 PMC11249116

[76] Li J Wu J Xie Y Yu X . Bone marrow adipocytes and lung cancer bone metastasis: unraveling the role of adipokines in the tumor microenvironment. Front Oncol. (2024) 14:1360471. doi: 10.3389/fonc.2024.1360471, PMID: 38571500 PMC10987778

[77] Marinelli Busilacchi E Morsia E Poloni A . Bone marrow adipose tissue. Cells. (2024) 13:724. doi: 10.3390/cells13090724, PMID: 38727260 PMC11083575

[78] Abend Bardagi A Dos Santos Paschoal C Favero GG Riccetto L Alexandrino Dias ML Guerra Junior G . Leptin's Immune Action: A Review Beyond Satiety. Immunol Invest. (2023) 52:117–133. doi: 10.1080/08820139.2022.2129381, PMID: 36278927

[79] Wei XQ Zhang YM Sun Y Ling HY He YN Fu JX . Bone Marrow Adipocytes Promote the Survival of Multiple Myeloma Cells and Up-Regulate Their Chemoresistance. Zhongguo Shi Yan Xue Ye Xue Za Zhi. (2023) 31:154–161. doi: 10.19746/j.cnki.issn.1009-2137.2023.01.025, PMID: 36765493

[80] Lu JJ Sun Y Zhang X Wang QQ Xiang ZY Ling YQ . Advances on pentraxin 3 in osteoporosis and fracture healing. Zhongguo Gu Shang. (2023) 36:393–8. doi: 10.12200/j.issn.1003-0034.2023.04.018, PMID: 37087632

[81] Mallick R Basak S Das RK Banerjee A Paul S Pathak S . Fatty Acids and their Proteins in Adipose Tissue Inflammation. Cell Biochem Biophys. (2024) 82:35–51. doi: 10.1007/s12013-023-01185-6, PMID: 37794302 PMC10867084

[82] Tian J Moon JS Nga HT Lee HY Nguyen TL Jang HJ . Brown fat-specific mitoribosomal function is crucial for preventing cold exposure-induced bone loss. Cell Mol Life Sci. (2024) 81:314. doi: 10.1007/s00018-024-05347-4, PMID: 39066814 PMC11335241

[83] Reiche ME Poels K Bosmans LA Vos WG Van Tiel CM Gijbels MJJ . Adipocytes control hematopoiesis and inflammation through CD40 signaling. Haematologica. (2023) 108:1873–1885. doi: 10.3324/haematol.2022.281482, PMID: 36475519 PMC10316249

[84] Li J Lu L Liu Y Yu X . Bone marrow adiposity during pathologic bone loss: molecular mechanisms underlying the cellular events. J Mol Med (Berl). (2022) 100:167–183. doi: 10.1007/s00109-021-02164-1, PMID: 34751809

[85] Li J Kang R Li G Li X Liu D Zhai L . MiR-30d-5p Regulates Bone Remodeling and Vessel Remodeling in Osteoporosis by Targeting GRP78. Faseb j. (2025) 39:e70781. doi: 10.1096/fj.202500345R, PMID: 40616398

[86] Pang Y Zhu S Ding P Zhang S Zhang Y Ye F . Long-Term High-Fat Diet Affected Bone Marrow Microenvironment During Aging at Single-Cell Resolution. MedComm. (2025) 6:e70276. doi: 10.1002/mco2.70276, PMID: 40692664 PMC12277656

[87] Guo Y Zhou H Wang Y Gu Y . Activated NETosis of bone marrow neutrophils up-regulates macrophage osteoclastogenesis via cGAS-STING/AKT2 pathway to promote osteoporosis. Exp Cell Res. (2025) 446:114477. doi: 10.1016/j.yexcr.2025.114477, PMID: 39988126

[88] Girard D Torossian F Oberlin E Alexander KA Gueguen J Tseng HW . Neurogenic Heterotopic Ossifications Recapitulate Hematopoietic Stem Cell Niche Development Within an Adult Osteogenic Muscle Environment. Front Cell Dev Biol. (2021) 9:611842. doi: 10.3389/fcell.2021.611842, PMID: 33748104 PMC7973025

[89] Ward CM Ravid K . Matrix Mechanosensation in the Erythroid and Megakaryocytic Lineages. Cells. (2020) 9. doi: 10.3390/cells9040894, PMID: 32268541 PMC7226728

[90] Kirkwood KL Zhang L Thiyagarajan R Seldeen KL Troen BR . Myeloid-Derived Suppressor Cells at the Intersection of Inflammaging and Bone Fragility. Immunol Invest. (2018) 47:844–854. doi: 10.1080/08820139.2018.1552360, PMID: 31282803 PMC7039312

[91] Dou C Ding N Zhao C Hou T Kang F Cao Z . Estrogen deficiency-mediated M2 macrophage osteoclastogenesis contributes to M1/M2 ratio alteration in ovariectomized osteoporotic mice. J Bone Miner Res. (2018) 33:899–908. doi: 10.1002/jbmr.3364, PMID: 29281118

[92] Liu Q Tian Y Zhao X Jing H Xie Q Li P . NMAAP1 expressed in BCG-activated macrophage promotes M1 macrophage polarization. Mol Cells. (2015) 38:886–94. doi: 10.14348/molcells.2015.0125, PMID: 26429502 PMC4625070

[93] Vuscan P Kischkel B Joosten LAB Netea MG . Microbial-induced trained immunity for cancer immunotherapy. Pharmacol Rev. (2025) 77:100074. doi: 10.1016/j.pharmr.2025.100074, PMID: 40616857

[94] Daman AW Antonelli AC Redelman-Sidi G Paddock L Khayat S Ketavarapu M . Microbial cancer immunotherapy reprograms hematopoiesis to enhance myeloid-driven anti-tumor immunity. Cancer Cell. (2025) 43:1442–59.e10. doi: 10.1016/j.ccell.2025.05.002, PMID: 40446799 PMC12377364

[95] Jia X Yang R Li J Zhao L Zhou X Xu X . Gut-bone axis: A non-negligible contributor to periodontitis. Front Cell Infect Microbiol. (2021) 11:752708. doi: 10.3389/fcimb.2021.752708, PMID: 34869062 PMC8637199

[96] Domínguez PM Ardavín C . Differentiation and function of mouse monocyte-derived dendritic cells in steady state and inflammation. Immunol Rev. (2010) 234:90–104. doi: 10.1111/j.0105-2896.2009.00876.x, PMID: 20193014

[97] Elsayed R Kurago Z Cutler CW Arce RM Gerber J Celis E . Role of dendritic cell-mediated immune response in oral homeostasis: A new mechanism of osteonecrosis of the jaw. FASEB J. (2020) 34:2595–608. doi: 10.1096/fj.201901819RR, PMID: 31919918 PMC7712496

[98] Sangsuwan R Thuamsang B Pacifici N Tachachartvanich P Murphy D Ram A . Identification of signaling networks associated with lactate modulation of macrophages and dendritic cells. Heliyon. (2025) 11:e42098. doi: 10.1016/j.heliyon.2025.e42098, PMID: 39975831 PMC11835580

[99] Hu S Xiang D Zhang X Zhang L Wang S Jin K . The mechanisms and cross-protection of trained innate immunity. Virol J. (2022) 19:210. doi: 10.1186/s12985-022-01937-5, PMID: 36482472 PMC9733056

[100] Sviridov D Netea MG Bukrinsky MI . Maladaptive trained immunity in viral infections. J Clin Invest. (2025) 135. doi: 10.1172/JCI192469, PMID: 40892509 PMC12404746

[101] Rahmani NR Belluomo R Kruyt MC Gawlitta D Joosten LAB Weinans H . Trained innate immunity modulates osteoblast and osteoclast differentiation. Stem Cell Rev Rep. (2024) 20:1121–34. doi: 10.1007/s12015-024-10711-9, PMID: 38478316 PMC11087362

[102] Wu D Cline-Smith A Shashkova E Perla A Katyal A Aurora R . T-cell mediated inflammation in postmenopausal osteoporosis. Front Immunol. (2021) 12:687551. doi: 10.3389/fimmu.2021.687551, PMID: 34276675 PMC8278518

[103] Yang S Zhu L Xiao L Shen Y Wang L Peng B . Imbalance of interleukin-17+ T-cell and Foxp3+ regulatory T-cell dynamics in rat periapical lesions. J Endod. (2014) 40:56–62. doi: 10.1016/j.joen.2013.09.033, PMID: 24331992

[104] Lighty AM Islam MA Smith BJ Ford Versypt AN . Mathematical modeling of estrogen-mediated inflammation in the gut and immune system. bioRxiv. (2024). doi: 10.1101/2024.12.23.630175, PMID: 39763984 PMC11703212

[105] Chen Y Chen M Liu Y Li Q Xue Y Liu L . Research progress of targeted therapy regulating Th17/Treg balance in bone immune diseases. Front Immunol. (2024) 15:1333993. doi: 10.3389/fimmu.2024.1333993, PMID: 38352872 PMC10861655

[106] Qi H Tian D Li M . Foxo3 promotes the differentiation and function of follicular helper T cells. Cell Rep. (2020) 31:107621. doi: 10.1016/j.celrep.2020.107621, PMID: 32402289

[107] Chen Y Chen M Liu Y . BAFF promotes follicular helper T cell development and germinal center formation through BR3 signal. JCI Insight. (2024) 9. doi: 10.1172/jci.insight.183400, PMID: 39325665 PMC11601555

[108] Lopes AMM Vieira JF Da Silva SFM . Dendritic cell immunotherapy has its antitumor action improved by the LPS in the maturation process. Clin Transl Oncol. (2025) 27:3501–10. doi: 10.1007/s12094-025-03858-5, PMID: 39979657

[109] Avivi I Zisman-Rozen S Naor S . Depletion of B cells rejuvenates the peripheral B-cell compartment but is insufficient to restore immune competence in aging. Aging Cell. (2019) 18:e12959. doi: 10.1111/acel.12959, PMID: 31056853 PMC6612643

[110] Azam Z Sapra L Baghel K Sinha N Gupta RK Soni V . Cissus quadrangularis (Hadjod) inhibits RANKL-induced osteoclastogenesis and augments bone health in an estrogen-deficient preclinical model of osteoporosis via modulating the host osteoimmune system. Cells. (2023) 12. doi: 10.3390/cells12020216, PMID: 36672152 PMC9857034

[111] Li Y Terauchi M Vikulina T Roser-Page S Weitzmann MN . B cell production of both OPG and RANKL is significantly increased in aged mice. Open Bone J. (2014) 6:8–17. doi: 10.2174/1876525401406010008, PMID: 25984250 PMC4429037

[112] Manabe N Kawaguchi H Chikuda H . Connection between B lymphocyte and osteoclast differentiation pathways. J Immunol. (2001) 167:2625–31. doi: 10.4049/jimmunol.167.5.2625, PMID: 11509604

[113] Frase D Lee C Nachiappan C Gupta R Akkouch A . The inflammatory contribution of B-lymphocytes and neutrophils in progression to osteoporosis. Cells. (2023) 12. doi: 10.3390/cells12131744, PMID: 37443778 PMC10340451

[114] Qi P Xie R Liu H . Mechanisms of gut homeostasis regulating Th17/Treg cell balance in PMOP. Front Immunol. (2024) 15:1497311. doi: 10.3389/fimmu.2024.1497311, PMID: 39735544 PMC11671525

[115] Li Y Ling J Jiang Q . Inflammasomes in alveolar bone loss. Front Immunol. (2021) 12:691013. doi: 10.3389/fimmu.2021.691013, PMID: 34177950 PMC8221428

[116] Kazemi-Sufi S Alipour S Rabieepour M Roshan-Milani S Naderi R . Serum proinflammatory cytokines, receptor activator of nuclear factor kappa-β ligand (RANKL), osteoprotegerin (OPG) and RANKL/OPG ratio in mild and severe COVID-19. BMC Infect Dis. (2024) 24:1047. doi: 10.1186/s12879-024-09941-6, PMID: 39333916 PMC11428542

[117] Zhong Z Qian Z Zhang X Chen F Ni S Kang Z . Tetrandrine prevents bone loss in ovariectomized mice by inhibiting RANKL-induced osteoclastogenesis. Front Pharmacol. (2019) 10:1530. doi: 10.3389/fphar.2019.01530, PMID: 31998129 PMC6967024

[118] Romas E Gillespie MT Martin TJ . Involvement of receptor activator of NFkappaB ligand and tumor necrosis factor-alpha in bone destruction in rheumatoid arthritis. Bone. (2002) 30:340–6. doi: 10.1016/S8756-3282(01)00682-2, PMID: 11856640

[119] Reziwan K Sun D Zhang B Zhao Z . MicroRNA-1225 activates Keap1-Nrf2-HO-1 signalling to inhibit TNFα-induced osteoclastogenesis by mediating ROS generation. Cell Biochem Funct. (2019) 37:256–265. doi: 10.1002/cbf.3394, PMID: 31017694

[120] Wu YH Zhang QL Mai SY Ming GX Zheng CF Liang CF . Strictosamide alleviates acute lung injury via regulating T helper 17 cells, regulatory T cells, and gut microbiota. Phytomedicine. (2024) 128:155490. doi: 10.1016/j.phymed.2024.155490, PMID: 38460358

[121] Qiao S Zhang X Chen Z Zhao Y Tzeng CM . Alloferon-1 ameliorates estrogen deficiency-induced osteoporosis through dampening the NLRP3/caspase-1/IL-1β/IL-18 signaling pathway. Int Immunopharmacol. (2023) 124:110954. doi: 10.1016/j.intimp.2023.110954, PMID: 37742365

[122] Zhang L Wang Q Su H Cheng J . Exosomes from adipose derived mesenchymal stem cells alleviate diabetic osteoporosis in rats through suppressing NLRP3 inflammasome activation in osteoclasts. J Biosci Bioeng. (2021) 131:671–678. doi: 10.1016/j.jbiosc.2021.02.007, PMID: 33849774

[123] De Leon-Oliva D Barrena-Blázquez S Jiménez-Álvarez L Fraile-Martinez O García-Montero C López-González L . The RANK-RANKL-OPG System: A Multifaceted Regulator of Homeostasis, Immunity, and Cancer. Medicina (Kaunas). (2023) 59. doi: 10.3390/medicina59101752, PMID: 37893470 PMC10608105

[124] Walsh MC Choi Y . Biology of the RANKL-RANK-OPG system in immunity, bone, and beyond. Front Immunol. (2014) 5:511. doi: 10.3389/fimmu.2014.00511, PMID: 25368616 PMC4202272

[125] Ueland T Bollerslev J Mosekilde L . Osteoclast function is regulated by neighbouring osteoblasts. Osteoprotegerin, RAND and RANK ligand constitute a unique regulatory system for bone resorption with important pathophysiological and therapeutic aspects. Ugeskr Laeger. (2002) 164:3526–30. 12116680

[126] Ninomiya H Fukuda S Nishida-Fukuda H Shibata Y Sato T Nakamichi Y . Osteoprotegerin secretion and its inhibition by RANKL in osteoblastic cells visualized using bioluminescence imaging. Bone. (2025) 191:117319. doi: 10.1016/j.bone.2024.117319, PMID: 39500402

[127] Wei SG Chen HH Xie LR Qin Y Mai YY Huang LH . RNA interference-mediated osteoprotegerin silencing increases the receptor activator of nuclear factor-kappa B ligand/osteoprotegerin ratio and promotes osteoclastogenesis. World J Stem Cells. (2025) 17:101290. doi: 10.4252/wjsc.v17.i4.101290, PMID: 40308885 PMC12038464

[128] Gersh FL O'Keefe JH Lavie CJ Henry BM . The Renin-Angiotensin-Aldosterone System in Postmenopausal Women: The Promise of Hormone Therapy. Mayo Clin Proc. (2021) 96:3130–3141. doi: 10.1016/j.mayocp.2021.08.009, PMID: 34736778

[129] Kireev RA Tresguerres AC Garcia C Borras C Ariznavarreta C Vara E . Hormonal regulation of pro-inflammatory and lipid peroxidation processes in liver of old ovariectomized female rats. Biogerontology. (2010) 11:229–43. doi: 10.1007/s10522-009-9242-2, PMID: 19633997

[130] Wang LT Chen LR Chen KH . Hormone-related and drug-induced osteoporosis: A cellular and molecular overview. Int J Mol Sci. (2023) 24. doi: 10.3390/ijms24065814, PMID: 36982891 PMC10054048

[131] Wang S Deng Z Seneviratne CJ Cheung GS Jin L Zhao B . Enterococcus faecalis promotes osteoclastogenesis and semaphorin 4D expression. Innate Immun. (2015) 21:726–35. doi: 10.1177/1753425915593162, PMID: 26138525

[132] Sun H Li Q Zhang Y Bi Y Li X Shu Y . Regulation of OPG and RANKL expressed by human dental follicle cells in osteoclastogenesis. Cell Tissue Res. (2015) 362:399–405. doi: 10.1007/s00441-015-2214-8, PMID: 26149648

[133] Andersen TL Abdelgawad ME Kristensen HB Hauge EM Rolighed L Bollerslev J . Understanding coupling between bone resorption and formation: are reversal cells the missing link?. Am J Pathol. (2013) 183:235–46. doi: 10.1016/j.ajpath.2013.03.006, PMID: 23747107

[134] Xiao W Li S Pacios S Wang Y Graves DT . Bone Remodeling Under Pathological Conditions. Front Oral Biol. (2016) 18:17–27. doi: 10.1159/000351896, PMID: 26599114 PMC10757467

[135] Chen Z Jiang M Mo L Zhou C Huang H Ma C . A natural agent, 5-deoxycajanin, mitigates estrogen-deficiency bone loss via modulating osteoclast-osteoblast homeostasis. Int Immunopharmacol. (2024) 141:112906. doi: 10.1016/j.intimp.2024.112906, PMID: 39173403

[136] Du J Qin W Wen F Zhao D Yin X Guo Z . Luteolin's Potential in Managing Osteoporosis and Bone Metabolism Disorders: Preclinical Insights. Drug Des Devel Ther. (2025) 19:9715–9732. doi: 10.2147/dddt.S547141, PMID: 41185709 PMC12579884

[137] Hamidouche Z Haÿ E Vaudin P Charbord P Schüle R Marie PJ . FHL2 mediates dexamethasone-induced mesenchymal cell differentiation into osteoblasts by activating Wnt/beta-catenin signaling-dependent Runx2 expression. Faseb j. (2008) 22:3813–22. doi: 10.1096/fj.08-106302, PMID: 18653765

[138] Rossini M Gatti D Adami S . Involvement of WNT/β-catenin signaling in the treatment of osteoporosis. Calcif Tissue Int. (2013) 93:121–32. doi: 10.1007/s00223-013-9749-z, PMID: 23748710

[139] Carrillo-López N Panizo S Alonso-Montes C Román-García P Rodríguez I Martínez-Salgado C . Direct inhibition of osteoblastic Wnt pathway by fibroblast growth factor 23 contributes to bone loss in chronic kidney disease. Kidney Int. (2016) 90:77–89. doi: 10.1016/j.kint.2016.01.024, PMID: 27165819

[140] Wang WL Tam PKH Chen Y . Abnormally activated wingless/integrated signaling modulates tumor-associated macrophage polarization and potentially promotes hepatocarcinoma cell growth. World J Gastroenterol. (2024) 30:4490–5. doi: 10.3748/wjg.v30.i41.4490, PMID: 39534418 PMC11551672

[141] Yuan C Yang D Ma J Yang J Xue J Song F . Modulation of Wnt/β-catenin signaling in IL-17A-mediated macrophage polarization of RAW264.7 cells. Braz J Med Biol Res. (2020) 53:e9488. doi: 10.1590/1414-431x20209488, PMID: 32578719 PMC7307890

[142] Wu X Zhang J Ma C Li W Zeng J Wang Y . A role for Wnt/β-catenin signalling in suppressing Bacillus Calmette-Guerin-induced macrophage autophagy. Microb Pathog. (2019) 127:277–287. doi: 10.1016/j.micpath.2018.12.016, PMID: 30550847

[143] Shi T Zhang Y Wang Y Song X Wang H Zhou X . DKK1 Promotes Tumor Immune Evasion and Impedes Anti-PD-1 Treatment by Inducing Immunosuppressive Macrophages in Gastric Cancer. Cancer Immunol Res. (2022) 10:1506–1524. doi: 10.1158/2326-6066.Cir-22-0218, PMID: 36206576

[144] Sun M Chen Z Wu X Yu Y Wang L Lu A . The Roles of Sclerostin in Immune System and the Applications of Aptamers in Immune-Related Research. Front Immunol. (2021) 12:602330. doi: 10.3389/fimmu.2021.602330, PMID: 33717084 PMC7946814

[145] Reppe S Refvem H Gautvik VT Olstad OK Høvring PI Reinholt FP . Eight genes are highly associated with BMD variation in postmenopausal Caucasian women. Bone. (2010) 46:604–12. doi: 10.1016/j.bone.2009.11.007, PMID: 19922823

[146] Secreto FJ Hoeppner LH Westendorf JJ . Wnt signaling during fracture repair. Curr Osteoporos Rep. (2009) 7:64–9. doi: 10.1007/s11914-009-0012-5, PMID: 19631031 PMC2972700

[147] Miao R Lim VY Kothapalli N Ma Y Fossati J Zehentmeier S . Hematopoietic stem cell niches and signals controlling immune cell development and maintenance of immunological memory. Front Immunol. (2020) 11:600127. doi: 10.3389/fimmu.2020.600127, PMID: 33324418 PMC7726109

[148] Liekens S Schols D Hatse S . CXCL12-CXCR4 axis in angiogenesis, metastasis and stem cell mobilization. Curr Pharm Des. (2010) 16:3903–20. doi: 10.2174/138161210794455003, PMID: 21158728

[149] Greenbaum A Hsu YM Day RB Schuettpelz LG Christopher MJ Borgerding JN . CXCL12 in early mesenchymal progenitors is required for haematopoietic stem-cell maintenance. Nature. (2013) 495:227–30. doi: 10.1038/nature11926, PMID: 23434756 PMC3600148

[150] Bonaud A Lemos JP Espéli M Balabanian K . Hematopoietic multipotent progenitors and plasma cells: neighbors or roommates in the mouse bone marrow ecosystem? Front Immunol. (2021) 12:658535. doi: 10.3389/fimmu.2021.658535, PMID: 33936091 PMC8083056

[151] Bachelerie F . CXCL12/CXCR4-axis dysfunctions: Markers of the rare immunodeficiency disorder WHIM syndrome. Dis Markers. (2010) 29:189–98. doi: 10.1155/2010/475104 PMC3835381 21178277

[152] Kumar R Milanesi S Szpakowska M Dotta L Di Silvestre D Trotta AM . Reduced G protein signaling despite impaired internalization and β-arrestin recruitment in patients carrying a CXCR4Leu317fsX3 mutation causing WHIM syndrome. JCI Insight. (2023) 8. doi: 10.1172/jci.insight.145688, PMID: 36883568 PMC10077478

[153] Lagane B Chow KY Balabanian K Levoye A Harriague J Planchenault T . CXCR4 dimerization and beta-arrestin-mediated signaling account for the enhanced chemotaxis to CXCL12 in WHIM syndrome. Blood. (2008) 112:34–44. doi: 10.1182/blood-2007-07-102103, PMID: 18436740

[154] García-Bernal D Redondo-Muñoz J Dios-Esponera A Chèvre R Bailón E Garayoa M . Sphingosine-1-phosphate activates chemokine-promoted myeloma cell adhesion and migration involving α4β1 integrin function. J Pathol. (2013) 229:36–48. doi: 10.1002/path.4066, PMID: 22711564

[155] Beider K Bitner H Leiba M Gutwein O Koren-Michowitz M Ostrovsky O . Multiple myeloma cells recruit tumor-supportive macrophages through the CXCR4/CXCL12 axis and promote their polarization toward the M2 phenotype. Oncotarget. (2014) 5:11283–96. doi: 10.18632/oncotarget.2207, PMID: 25526031 PMC4294328

[156] Burger JA Peled A . CXCR4 antagonists: targeting the microenvironment in leukemia and other cancers. Leukemia. (2009) 23:43–52. doi: 10.1038/leu.2008.299, PMID: 18987663

[157] Li N Wang Y Xu H Wang H Gao Y Zhang Y . Exosomes Derived from RM-1 Cells Promote the Recruitment of MDSCs into Tumor Microenvironment by Upregulating CXCR4 via TLR2/NF-κB Pathway. J Oncol. (2021) 2021:5584406. doi: 10.1155/2021/5584406, PMID: 34659412 PMC8519695

[158] Patterson SD Copland M . The bone marrow immune microenvironment in CML: treatment responses, treatment-free remission, and therapeutic vulnerabilities. Curr Hematol Malig Rep. (2023) 18:19–32. doi: 10.1007/s11899-023-00688-6, PMID: 36780103 PMC9995533

[159] Lim H Park H Kim HP . Effects of flavonoids on senescence-associated secretory phenotype formation from bleomycin-induced senescence in BJ fibroblasts. Biochem Pharmacol. (2015) 96:337–48. doi: 10.1016/j.bcp.2015.06.013, PMID: 26093063

[160] Ho YH Méndez-Ferrer S . Microenvironmental contributions to hematopoietic stem cell aging. Haematologica. (2020) 105:38–46. doi: 10.3324/haematol.2018.211334, PMID: 31806690 PMC6939521

[161] Hoffman CM Han J Calvi LM . Impact of aging on bone, marrow and their interactions. Bone. (2019) 119:1–7. doi: 10.1016/j.bone.2018.07.012, PMID: 30010082

[162] Wang J Xin Y Dong Z Li S Yang G . The interplay between the immune microenvironment and bone aging: From molecular mechanisms to therapeutic interventions. Exp Gerontol. (2025) 212:112974. doi: 10.1016/j.exger.2025.112974, PMID: 41297720

[163] Chandra A Law SF Pignolo RJ . Changing landscape of hematopoietic and mesenchymal cells and their interactions during aging and in age-related skeletal pathologies. Mech Ageing Dev. (2025) 225:112059. doi: 10.1016/j.mad.2025.112059, PMID: 40220914 PMC12103995

[164] Chen YH Zhang X Attarian D Kraus VB . Synergistic roles of CBX4 chromo and SIM domains in regulating senescence of primary human osteoarthritic chondrocytes. Arthritis Res Ther. (2023) 25:197. doi: 10.1186/s13075-023-03183-8, PMID: 37828576 PMC10568837

[165] Lopes-Paciencia S Saint-Germain E Rowell MC Ruiz AF Kalegari P Ferbeyre G . The senescence-associated secretory phenotype and its regulation. Cytokine. (2019) 117:15–22. doi: 10.1016/j.cyto.2019.01.013, PMID: 30776684

[166] Li B Lyu P Tang J Li J Ouchi T Fan Y . The Potential Role and Therapeutic Relevance of Cellular Senescence in Skeletal Pathophysiology. J Gerontol A Biol Sci Med Sci. (2024) 79. doi: 10.1093/gerona/glae037, PMID: 38306619

[167] Hoong CWS Saul D Khosla S Sfeir JG . Advances in the management of osteoporosis. Bmj. (2025) 390:e081250. doi: 10.1136/bmj-2024-081250, PMID: 40738610

[168] Lawrence M Goyal A Pathak S Ganguly P . Cellular Senescence and Inflammaging in the Bone: Pathways, Genetics, Anti-Aging Strategies and Interventions. Int J Mol Sci. (2024) 25. doi: 10.3390/ijms25137411, PMID: 39000517 PMC11242738

[169] Andersson A Törnqvist AE Moverare-Skrtic S Bernardi AI Farman HH Chambon P . Roles of activating functions 1 and 2 of estrogen receptor α in lymphopoiesis. J Endocrinol. (2018) 236:99–109. doi: 10.1530/joe-17-0372, PMID: 29255084

[170] Wärnmark A Almlöf T Leers J Gustafsson JA Treuter E . Differential recruitment of the mammalian mediator subunit TRAP220 by estrogen receptors ERalpha and ERbeta. J Biol Chem. (2001) 276:23397–404. doi: 10.1074/jbc.M011651200, PMID: 11303023

[171] Schreihofer DA Rowe DF Rissman EF Scordalakes EM Gustafsson JJ Shupnik MA . Estrogen receptor-alpha (ERalpha), but not ERbeta, modulates estrogen stimulation of the ERalpha-truncated variant, TERP-1. Endocrinology. (2002) 143:4196–202. doi: 10.1210/en.2002-220353, PMID: 12399412

[172] Lou Y Fu Z Tian Y Hu M Wang Q Zhou Y . Estrogen-sensitive activation of SGK1 induces M2 macrophages with anti-inflammatory properties and a Th2 response at the maternal-fetal interface. Reprod Biol Endocrinol. (2023) 21:50. doi: 10.1186/s12958-023-01102-9, PMID: 37226177 PMC10207684

[173] Fernandes G Lawrence R Sun D . Protective role of n-3 lipids and soy protein in osteoporosis. Prostaglandins Leukot Essent Fatty Acids. (2003) 68:361–72. doi: 10.1016/S0952-3278(03)00060-7, PMID: 12798656

[174] Hansdah K Lui JC . Emerging insights into the endocrine regulation of bone homeostasis by gut microbiome. J Endocr Soc. (2024) 8:bvae117. doi: 10.1210/jendso/bvae117, PMID: 38957653 PMC11215793

[175] Schneider AE Kárpáti E Schuszter K Tóth EA Kiss E Kulcsár M . A dynamic network of estrogen receptors in murine lymphocytes: fine-tuning the immune response. J Leukoc Biol. (2014) 96:857–72. doi: 10.1189/jlb.2A0214-080RR, PMID: 25070950

[176] Fu Z Li M Zhou H Zhong X Zhang Z Meng X . Endoplasmic reticulum stress orchestrates tumor metabolism and immunity: new insights into immunometabolic therapeutics. Front Immunol. (2025) 16:1674163. doi: 10.3389/fimmu.2025.1674163, PMID: 41098732 PMC12518273

[177] Lo YL Lin HC Li CY Huang B Yang CP Chuang HY . Functional pH-Responsive Nanoparticles for Immune Reprogramming in MSS Colorectal Cancer via ER Stress-Induced Proteostasis Disruption, PD-L1-Targeting miRNA, and TLR7 Activation. Pharmaceutics. (2025) 17. doi: 10.3390/pharmaceutics17111503, PMID: 41304839 PMC12656422

[178] Salminen A Kaarniranta K Kauppinen A . The role of myeloid-derived suppressor cells (MDSC) in the inflammaging process. Ageing Res Rev. (2018) 48:1–10. doi: 10.1016/j.arr.2018.09.001, PMID: 30248408

[179] Basu S Ulbricht Y Rossol M . Healthy and premature aging of monocytes and macrophages. Front Immunol. (2025) 16:1506165. doi: 10.3389/fimmu.2025.1506165, PMID: 40165963 PMC11955604

[180] Nishida Y Terkawi MA Matsumae G Yokota S Tokuhiro T Ogawa Y . Dynamic transcriptome analysis of osteal macrophages identifies a distinct subset with senescence features in experimental osteoporosis. JCI Insight. (2024) 9. doi: 10.1172/jci.insight.182418, PMID: 39480497 PMC11623942

[181] Fu Y Meng L Zhang M Li L Liu H Ma W . ED-71 alleviates OVX-induced osteoporosis by inhibiting macrophage senescence through SIRT1/PGC-1α pathway: A potential therapeutic approach. Bone. (2026) 203:117737. doi: 10.1016/j.bone.2025.117737, PMID: 41290109

[182] Zhou Q Wang K Wang C Sun X Wang L Sun J . Wen-Shen-Tong-Luo-Zhi-Tong Decoction alleviates bone loss in aged mice by suppressing LONP1-mediated macrophage senescence. Pharm Biol. (2025) 63:524–548. doi: 10.1080/13880209.2025.2537125, PMID: 40719285 PMC12305870

[183] Massaro F Corrillon F Stamatopoulos B Dubois N Ruer A Meuleman N . Age-related changes in human bone marrow mesenchymal stromal cells: morphology, gene expression profile, immunomodulatory activity and miRNA expression. Front Immunol. (2023) 14:1267550. doi: 10.3389/fimmu.2023.1267550, PMID: 38130717 PMC10733451

[184] Aurora R Veis D . Does aging activate T-cells to reduce bone mass and quality? Curr Osteoporos Rep. (2022) 20:326–33. doi: 10.1007/s11914-022-00745-8, PMID: 36044177 PMC10016147

[185] Kverka M Stepan JJ . Associations among estrogens, the gut microbiome and osteoporosis. Curr Osteoporos Rep. (2024) 23:2. doi: 10.1007/s11914-024-00896-w, PMID: 39585466 PMC11588883

[186] Zhang S Ni W . High systemic immune-inflammation index is relevant to osteoporosis among middle-aged and older people: A cross-sectional study. Immun Inflammation Dis. (2023) 11:e992. doi: 10.1002/iid3.992, PMID: 37647432 PMC10465993

[187] Li B Li J Li B Ouchi T Li L Li Y . A single-cell transcriptomic atlas characterizes age-related changes of murine cranial stem cell niches. Aging Cell. (2023) 22:e13980. doi: 10.1111/acel.13980, PMID: 37681346 PMC10652347

[188] Zhao X Patil S Qian A Zhao C . Bioactive Compounds of Polygonatum sibiricum - Therapeutic Effect and Biological Activity. Endocr Metab Immune Disord Drug Targets. (2022) 22:26–37. doi: 10.2174/1871530321666210208221158, PMID: 33563164

[189] Bai L Liu Y Zhang X Chen P Hang R Xiao Y . Osteoporosis remission via an anti-inflammaging effect by icariin activated autophagy. Biomaterials. (2023) 297:122125. doi: 10.1016/j.biomaterials.2023.122125, PMID: 37058900

[190] Günther M Paczia N Michels S . Cimicifuga racemosa extract Ze 450 shifts macrophage immunometabolism and attenuates pro-inflammatory signaling. BioMed Pharmacother. (2025) 188:118130. doi: 10.1016/j.biopha.2025.118130, PMID: 40382826

[191] Fagundes CT Amaral FA Vieira AT . Transient TLR activation restores inflammatory response and ability to control pulmonary bacterial infection in germfree mice. J Immunol. (2012) 188:1411–20. doi: 10.4049/jimmunol.1101682, PMID: 22210917

[192] Pacifici R . Role of gut microbiota in the skeletal response to PTH. J Clin Endocrinol Metab. (2021) 106:636–45. doi: 10.1210/clinem/dgaa895, PMID: 33254225 PMC7947780

[193] Li JY Yu M Pal S Tyagi AM Dar H Adams J . Parathyroid hormone-dependent bone formation requires butyrate production by intestinal microbiota. J Clin Invest. (2020) 130:1767–1781. doi: 10.1172/jci133473, PMID: 31917685 PMC7108906

[194] Pal S Perrien DS Yumoto T Faccio R Stoica A Adams J . The microbiome restrains melanoma bone growth by promoting intestinal NK and Th1 cell homing to bone. J Clin Invest. (2022) 132. doi: 10.1172/jci157340, PMID: 35503658 PMC9197523

[195] Wells C Robertson T Sheth P Abraham S . How aging influences the gut-bone marrow axis and alters hematopoietic stem cell regulation. Heliyon. (2024) 10:e32831. doi: 10.1016/j.heliyon.2024.e32831, PMID: 38984298 PMC11231543

[196] Chen Y Xie Y Yu X . Progress of research on the gut microbiome and its metabolite short-chain fatty acids in postmenopausal osteoporosis: a literature review. Front Med. (2025) 19:474–92. doi: 10.1007/s11684-025-1129-3, PMID: 40347368

[197] Yang B Wu J Hou X Bai T Liu S . Memory in Misfire: The Gut Microbiome-Trained Immunity Circuit in Inflammatory Bowel Diseases. Int J Mol Sci. (2025) 26. doi: 10.3390/ijms26199663, PMID: 41096928 PMC12524489

[198] Lv J Chen H Ni Y Zhang Y Huang X . Clostridium butyricum attenuates radiation-induced bone loss through gut microbiota and immune regulation in mice. Microbiol Res. (2026) 302:128352. doi: 10.1016/j.micres.2025.128352, PMID: 41016376

[199] Green RS Roy T Morales DD Morrow C Neilson R Schott EM . A synbiotic medical food improves gut barrier function, reduces immune responses, and inhibits osteoclast activity in models of postmenopausal bone loss aligned with clinical outcomes. bioRxiv. (2025). doi: 10.1101/2025.08.07.669142, PMID: 41584438 PMC12829910

[200] Gong Y Ma X Huang J Zhang P Hai Y Song Y . Akkermansia muciniphila and osteoporosis: emerging role of gut microbiota in skeletal homeostasis. Front Microbiol. (2025) 16:1665101. doi: 10.3389/fmicb.2025.1665101, PMID: 41030554 PMC12477698

[201] Suzuki R Chen L Endo T Tokuhiro T Nakajo M Ogawa Y . Transcriptomic profiling reveals a dramatic inflammatory shift in osteal macrophages during colitis-induced osteoporosis. Inflamm Res. (2025) 74:165. doi: 10.1007/s00011-025-02139-9, PMID: 41266820

[202] You Y Xiang T Yang C Xiao S Tang Y Luo G . Interactions between the gut microbiota and immune cell dynamics: novel insights into the gut-bone axis. Gut Microbes. (2025) 17:2545417. doi: 10.1080/19490976.2025.2545417, PMID: 40873417 PMC12396131

[203] Zhang YW Cao MM Li YJ Dai GC Lu PP Zhang M . The regulative effect and repercussion of probiotics and prebiotics on osteoporosis: involvement of brain-gut-bone axis. Crit Rev Food Sci Nutr. (2023) 63:7510–7528. doi: 10.1080/10408398.2022.2047005, PMID: 35234534

[204] Gu C Du H Li N Zhou Y Li S Sun Y . The gut-bone axis in osteoporosis: a multifaceted interaction with implications for bone health. Front Endocrinol (Lausanne). (2025) 16:1569152. doi: 10.3389/fendo.2025.1569152, PMID: 40741168 PMC12307166

[205] Hao F Guo M Zhao Y Zhu X Hu X Zhu W . Qing'e Pills Ameliorates Osteoporosis by Regulating Gut Microbiota and Th17/Treg Balance in Ovariectomized Rats. J Inflamm Res. (2025) 18:7611–7629. doi: 10.2147/jir.S517176, PMID: 40524965 PMC12168940

[206] Lisco G Triggiani D Giagulli VA De Pergola G Guastamacchia E Piazzolla G . Endocrine, Metabolic, and Immune Pathogenesis of Postmenopausal Osteoporosis. Is there a Therapeutic Role in Natural Products?. Endocr Metab Immune Disord Drug Targets. (2023) 23:1278–1290. doi: 10.2174/1871530323666230330121301, PMID: 37005529

[207] Rogers MJ . New insights into the molecular mechanisms of action of bisphosphonates. Curr Pharm Des. (2003) 9:2643–58. doi: 10.2174/1381612033453640, PMID: 14529538

[208] Mac-Way F Trombetti A Noel C Lafage-Proust MH . Giant osteoclasts in patients under bisphosphonates. BMC Clin Pathol. (2014) 14:31. doi: 10.1186/1472-6890-14-31, PMID: 25024641 PMC4094788

[209] Van Acker HH Anguille S Willemen Y Smits EL Van Tendeloo VF . Bisphosphonates for cancer treatment: Mechanisms of action and lessons from clinical trials. Pharmacol Ther. (2016) 158:24–40. doi: 10.1016/j.pharmthera.2015.11.008, PMID: 26617219

[210] Ferbebouh M Vallières F Benderdour M Fernandes J . The pathophysiology of immunoporosis: innovative therapeutic targets. Inflamm Res. (2021) 70:859–875. doi: 10.1007/s00011-021-01484-9, PMID: 34272579

[211] Lewiecki EM . Clinical use of denosumab for the treatment for postmenopausal osteoporosis. Curr Med Res Opin. (2010) 26:2807–12. doi: 10.1185/03007995.2010.533651, PMID: 21050058

[212] Chiu YG Ritchlin CT . Denosumab: targeting the RANKL pathway to treat rheumatoid arthritis. Expert Opin Biol Ther. (2017) 17:119–28. doi: 10.1080/14712598.2017.1263614, PMID: 27871200 PMC5794005

[213] Liu YCG Teng AY . Potential contribution of immature myeloid CD11c(+)dendritic cells-derived osteoclast precursor to inflammation-induced bone loss in the TRAF6-null chimeras *in-vivo*. J Dent Sci. (2023) 18:1372–7. doi: 10.1016/j.jds.2023.03.016, PMID: 37404624 PMC10316501

[214] Liao SS Deng YL Hsu CY Lee HT Li CR Yang CC . Denosumab in the Management of Glucocorticoid-Induced Osteoporosis: Long-Term Efficacy and Secondary Fracture Outcomes. J Clin Med. (2025) 14. doi: 10.3390/jcm14051633, PMID: 40095574 PMC11900549

[215] Al Khayyat SG Falsetti P Conticini E D'Alessandro R Bellisai F Gentileschi S . Bone-sparing effects of rituximab and body composition analysis in a cohort of postmenopausal women affected by rheumatoid arthritis - retrospective study. Reumatologia. (2021) 59:206–210. doi: 10.5114/reum.2021.108430, PMID: 34538950 PMC8436793

[216] Zhao Z Deng Y Li L Zhu L Wang X Sun H . Enhancing Akkermansia growth via phytohormones: a strategy to modulate the gut-bone axis in postmenopausal osteoporosis therapy. J Transl Med. (2025) 23:410. doi: 10.1186/s12967-025-06426-1, PMID: 40205438 PMC11984252

[217] Kallio A Guo T Lamminen E Seppänen J Kangas L Väänänen HK . Estrogen and the selective estrogen receptor modulator (SERM) protection against cell death in estrogen receptor alpha and beta expressing U2OS cells. Mol Cell Endocrinol. (2008) 289:38–48. doi: 10.1016/j.mce.2008.03.005, PMID: 18455292

[218] Li S Liu G Hu S . Osteoporosis: interferon-gamma-mediated bone remodeling in osteoimmunology. Front Immunol. (2024) 15:1396122. doi: 10.3389/fimmu.2024.1396122, PMID: 38817601 PMC11137183

[219] Bonnet AL Aboishava L Mannstadt M . Advances in parathyroid hormone-based medicines. J Bone Miner Res. (2025) 40:1195–206. doi: 10.1093/jbmr/zjaf118, PMID: 40847810 PMC12578286

[220] Pigeaud KE Rietveld ML Witvliet AF Hogervorst JMA Zhang C Forouzanfar T . The Effect of Sclerostin and Monoclonal Sclerostin Antibody Romosozumab on Osteogenesis and Osteoclastogenesis Mediated by Periodontal Ligament Fibroblasts. Int J Mol Sci. (2023) 24. doi: 10.3390/ijms24087574, PMID: 37108735 PMC10145870

[221] Chicana B Donham C Millan AJ Manilay JO . Wnt Antagonists in Hematopoietic and Immune Cell Fate: Implications for Osteoporosis Therapies. Curr Osteoporos Rep. (2019) 17:49–58. doi: 10.1007/s11914-019-00503-3, PMID: 30835038 PMC6715281

[222] Gupta N Kanwar N Arora A Khatri K Kanwal A . The interplay of rheumatoid arthritis and osteoporosis: exploring the pathogenesis and pharmacological approaches. Clin Rheumatol. (2024) 43:1421–1433. doi: 10.1007/s10067-024-06932-5, PMID: 38499817

[223] De Martinis M Sirufo MM Ginaldi L . Osteoporosis: current and emerging therapies targeted to immunological checkpoints. Curr Med Chem. (2020) 27:6356–72. doi: 10.2174/0929867326666190730113123, PMID: 31362684 PMC8206194

[224] Smajlović H Prohić A . PAPA syndrome: challenges in achieving long-term remission. Acta Dermatovenerol Croat. (2023) 31:106–9. 38006373

[225] Kaneki H Guo R Chen D Yao Z Schwarz EM Zhang YE . Tumor necrosis factor promotes Runx2 degradation through up-regulation of Smurf1 and Smurf2 in osteoblasts. J Biol Chem. (2006) 281:4326–33. doi: 10.1074/jbc.M509430200, PMID: 16373342 PMC2647592

[226] Zhang W Gao R Rong X Zhu S Cui Y Liu H . Immunoporosis: Role of immune system in the pathophysiology of different types of osteoporosis. Front Endocrinol (Lausanne). (2022) 13:965258. doi: 10.3389/fendo.2022.965258, PMID: 36147571 PMC9487180

[227] Liu Y Yan C Li Y Zhou R Lin X Meng Q . Single-cell RNA sequencing of peripheral blood mononuclear cells from bronchopulmonary dysplasia. Clin Transl Med. (2025) 15:e70276. doi: 10.1002/ctm2.70276, PMID: 40097329 PMC11913593

[228] Hong M Liao Y Liang J Chen X Li S Liu W . Immunomodulation of human CD19(+)CD25(high) regulatory B cells via Th17/Foxp3 regulatory T cells and Th1/Th2 cytokines. Hum Immunol. (2019) 80:863–870. doi: 10.1016/j.humimm.2019.05.011, PMID: 31262519

[229] Cai X Li Z Yao Y Zheng Y Zhang M Ye Y . Glycolithocholic acid increases the frequency of circulating Tregs through constitutive androstane receptor to alleviate postmenopausal osteoporosis. Biochem Pharmacol. (2024) 219:115951. doi: 10.1016/j.bcp.2023.115951, PMID: 38036190

[230] Sapra L Saini C Mishra PK Garg B Gupta S Manhas V . Bacillus coagulans ameliorates inflammatory bone loss in post-menopausal osteoporosis via modulating the "Gut-Immune-Bone" axis. Gut Microbes. (2025) 17:2492378. doi: 10.1080/19490976.2025.2492378, PMID: 40275534 PMC12036487

[231] Hsieh TY Lui SW Lu JW Chen YC Lin TC Jheng WL . Using Treg, Tr1, and Breg Expression Levels to Predict Clinical Responses to csDMARD Treatment in Drug-naive Patients With Rheumatoid Arthritis. In Vivo. (2023) 37:2018–2027. doi: 10.21873/invivo.13299, PMID: 37652509 PMC10500538

[232] Chu J Qin R Wang SJ Wang Q Wu Q . Integrated single-cell and transcriptomic analysis of bone marrow-derived metastatic neuroblastoma reveals molecular mechanisms of metabolic reprogramming. Sci Rep. (2025) 15:28519. doi: 10.1038/s41598-025-13626-8, PMID: 40764361 PMC12325718

[233] Lakshmanachetty S Cruz-Cruz J Hoffmeyer E Cole AP Mitra SS . New Insights into the Multifaceted Role of Myeloid-Derived Suppressor Cells (MDSCs) in High-Grade Gliomas: From Metabolic Reprograming, Immunosuppression, and Therapeutic Resistance to Current Strategies for Targeting MDSCs. Cells. (2021) 10. doi: 10.3390/cells10040893, PMID: 33919732 PMC8070707

[234] Liu R Zhu J Chen A Fan Y Li L Mei Y . Intra-bone marrow injection with engineered Lactococcus lactis for the treatment of metastatic tumors: Primary report. Biomed Pharmacother. (2024) 173:116384. doi: 10.1016/j.biopha.2024.116384, PMID: 38471270

[235] Wang B Bai J Tian B Chen H Yang Q Chen Y . Genetically Engineered Hematopoietic Stem Cells Deliver TGF-β Inhibitor to Enhance Bone Metastases Immunotherapy. Adv Sci (Weinh). (2022) 9:e2201451. doi: 10.1002/advs.202201451, PMID: 35948516 PMC9534984

[236] Jafari Z Hamzehpour Z Faghihi M Derazi RA Makoui MH Makoui RH . Modified-Mesenchymal Stem Cells as an Innovative Therapeutic Approach in Systemic Lupus Erythematosus (SLE); A Comprehensive Review. Mod Rheumatol. (2025). doi: 10.1093/mr/roaf112, PMID: 41273077

[237] Wang J Zhang L Cui X Xu X Guo R Li K . Bcl11a maintains hematopoietic stem cell function but accelerates inflammation-driven exhaustion during aging. Sci Immunol. (2025) 10:eadr2041. doi: 10.1126/sciimmunol.adr2041, PMID: 40215324

[238] Helbling PM Piñeiro-Yáñez E Gerosa R Boettcher S Al-Shahrour F Manz MG . Global transcriptomic profiling of the bone marrow stromal microenvironment during postnatal development, aging, and inflammation. Cell Rep. (2019) 29:3313–30.e4. doi: 10.1016/j.celrep.2019.11.004, PMID: 31801092

[239] Cui J Li X Liu B Dong C Chang Y . Hematopoietic stem cell aging: mechanisms, microenvironment influences, and rejuvenation strategies. Bioengineering (Basel). (2025) 12. doi: 10.3390/bioengineering12111166, PMID: 41301122 PMC12649273

[240] Müller L Di Benedetto S . Aging brain: exploring the interplay between bone marrow aging, immunosenescence, and neuroinflammation. Front Immunol. (2024) 15:1393324. doi: 10.3389/fimmu.2024.1393324, PMID: 38638424 PMC11024322

[241] Robbins PD Jurk D Khosla S Kirkland JL LeBrasseur NK Miller JD . Senolytic drugs: reducing senescent cell viability to extend health span. Annu Rev Pharmacol Toxicol. (2021) 61:779–803. doi: 10.1146/annurev-pharmtox-050120-105018, PMID: 32997601 PMC7790861

[242] Chang J Wang Y Shao L Laberge RM Demaria M Campisi J . Clearance of senescent cells by ABT263 rejuvenates aged hematopoietic stem cells in mice. Nat Med. (2016) 22:78–83. doi: 10.1038/nm.4010, PMID: 26657143 PMC4762215

[243] Singh A Yashavarddhan MH Kalita B Ranjan R Bajaj S Prakash H . Podophyllotoxin and rutin modulates ionizing radiation-induced oxidative stress and apoptotic cell death in mice bone marrow and spleen. Front Immunol. (2017) 8:183. doi: 10.3389/fimmu.2017.00183, PMID: 28289414 PMC5326804

[244] Li H Hu F Pan X Wang Q Liang H Lv L . Multidimensional regulation of bone marrow niche using extracorporeal shock wave responsive nanocomposites for osteoporosis therapy. Small. (2025) 21:e05863. doi: 10.1002/smll.202505863, PMID: 40911703

[245] Salamanna F Contartese D Errani C Sartori M Borsari V Giavaresi G . Role of bone marrow adipocytes in bone metastasis development and progression: a systematic review. Front Endocrinol (Lausanne). (2023) 14:1207416. doi: 10.3389/fendo.2023.1207416, PMID: 37711896 PMC10497772

[246] Bai L Zhang X Shen W Wang P Yin X Liu J . Multifunctional scaffold comprising metal-organic framework, hydrogel, and demineralized bone matrix for the treatment of steroid-induced femoral head necrosis. Small. (2025) 21:e2407758. doi: 10.1002/smll.202407758, PMID: 39575484

[247] Liu H Wei J Xiao S Jin S Yuan L Wen J . Regulation energy metabolism of fiber scaffolds orchestrates osteoimmunomodulation and angio/osteogenesis. Small. (2025) 21:e2409747. doi: 10.1002/smll.202409747, PMID: 40135330

[248] Lyu Z Yuan G Zhang Y Zhang F Liu Y Li Y . Anaerostipes caccae CML199 enhances bone development and counteracts aging-induced bone loss through the butyrate-driven gut-bone axis: the chicken model. Microbiome. (2024) 12:215. doi: 10.1186/s40168-024-01920-y, PMID: 39438898 PMC11495078

[249] Spiliopoulou P Rousakis P Panteli C Eleutherakis-Papaiakovou E Migkou M Kanellias N . Effects of exercise training on the bone marrow immune microenvironment and minimal residual disease in multiple myeloma patients following first-line treatment. Scand J Med Sci Sports. (2025) 35:e70020. doi: 10.1111/sms.70020, PMID: 39853819 PMC11760657

[250] Morgan KT . Nutritional determinants of bone health. J Nutr Elder. (2008) 27:3–27. doi: 10.1080/01639360802059670, PMID: 18928188

[251] Feng Y Dang X Zheng P Liu Y Liu D Che Z . Quercetin in osteoporosis treatment: A comprehensive review of its mechanisms and therapeutic potential. Curr Osteoporos Rep. (2024) 22:353–65. doi: 10.1007/s11914-024-00868-0, PMID: 38652430

[252] Papalexi E Satija R . Single-cell RNA sequencing to explore immune cell heterogeneity. Nat Rev Immunol. (2018) 18:35–45. doi: 10.1038/nri.2017.76, PMID: 28787399

[253] Lutz R Poos AM Solé-Boldo L John L Wagner J Prokoph N . Bone marrow breakout lesions act as key sites for tumor-immune cell diversification in multiple myeloma. Sci Immunol. (2025) 10:eadp6667. doi: 10.1126/sciimmunol.adp6667, PMID: 39919199 PMC7619224

[254] Li F Cai J Liu J Yu SC Zhang X Su Y . Construction of a solid Cox model for AML patients based on multiomics bioinformatic analysis. Front Oncol. (2022) 12:925615. doi: 10.3389/fonc.2022.925615, PMID: 36033493 PMC9399435

[255] Gui G Bingham MA Herzog JR Wong-Rolle A Dillon LW Goswami M . Single-cell spatial transcriptomics reveals immunotherapy-driven bone marrow niche remodeling in AML. Sci Adv. (2025) 11:eadw4871. doi: 10.1126/sciadv.adw4871, PMID: 40632867 PMC12239967

[256] Cui C Cui F Zou Q Zhang Z Jia L . Progress and applications of single-cell RNA sequencing and spatial transcriptome technology in acute kidney injury research. Mol Ther Nucleic Acids. (2025) 36:102583. doi: 10.1016/j.omtn.2025.102583, PMID: 40568028 PMC12192682

[257] Zhang T Wu Z Li L Ren J Zhang Z Wang G . CPPLS-MLP: a method for constructing cell-cell communication networks and identifying related highly variable genes based on single-cell sequencing and spatial transcriptomics data. Brief Bioinform. (2024) 25. doi: 10.1093/bib/bbae198, PMID: 38678387 PMC11056015

[258] Little-Letsinger SE Hamilton SE . Leveraging mice with diverse microbial exposures for advances in osteoimmunology. Front Endocrinol (Lausanne). (2023) 14:1168552. doi: 10.3389/fendo.2023.1168552, PMID: 37251680 PMC10210590

[259] Ferrena A Wang J Zhang R Karadal-Ferrena B Al-Hardan W Singh S . SKP2 knockout in rb1/p53-deficient mouse models of osteosarcoma induces immune infiltration and drives a transcriptional program with a favorable prognosis. Mol Cancer Ther. (2024) 23:223–34. doi: 10.1158/1535-7163.MCT-23-0173, PMID: 37871911 PMC10842346

[260] Duke VR Philippon MJ Lind DRG Kasler H Yamaura K Huard M . Murine progeria model exhibits delayed fracture healing with dysregulated local immune response. bioRxiv. (2024). doi: 10.1101/2024.05.29.596277, PMID: 38854043 PMC11160782

[261] Yang D Wan Y . Molecular determinants for the polarization of macrophage and osteoclast. Semin Immunopathol. (2019) 41:551–63. doi: 10.1007/s00281-019-00754-3, PMID: 31506868 PMC6815265

[262] Papadimitriou K Tsakirakis N Malandrakis P Vitsos P Metousis A Orologas-Stavrou N . Deep phenotyping reveals distinct immune signatures correlating with prognostication, treatment responses, and MRD status in multiple myeloma. Cancers (Basel). (2020) 12. doi: 10.3390/cancers12113245, PMID: 33158030 PMC7692501

[263] Alibrahim MN Carbone A Alsaleh N Gloghini A . Immune checkpoint molecules in hodgkin lymphoma and other hematological Malignancies. Cancers (Basel). (2025) 17. doi: 10.3390/cancers17142292, PMID: 40723175 PMC12293259

[264] Taylor AM Sheng J Ng PKS Harder JM Kumar P Ahn JY . Immunosuppressive tumor microenvironment of osteosarcoma. Cancers (Basel). (2025) 17. doi: 10.3390/cancers17132117, PMID: 40647416 PMC12248827

[265] Wan Z Bai X Wang X Guo X Wang X Zhai M . Mgp high-expressing MSCs orchestrate the osteoimmune microenvironment of collagen/nanohydroxyapatite-mediated bone regeneration. Adv Sci (Weinh). (2024) 11:e2308986. doi: 10.1002/advs.202308986, PMID: 38588510 PMC11187922

[266] Verheye E Kancheva D Satilmis H Vandewalle N Fan R Bardet PMR . A single-cell transcriptomic map of the murine and human multiple myeloma immune microenvironment across disease stages. J Hematol Oncol. (2024) 17:107. doi: 10.1186/s13045-024-01629-3, PMID: 39511632 PMC11546219

[267] Liu Z Zhang S Li H Guo J Wu D Zhou W . Cellular interaction analysis characterizing immunosuppressive microenvironment functions in MM tumorigenesis from precursor stages. Front Genet. (2022) 13:844604. doi: 10.3389/fgene.2022.844604, PMID: 35401705 PMC8984155

[268] Ye X Huang X Fu X Zhang X Lin R Zhang W . Myeloid-like tumor hybrid cells in bone marrow promote progression of prostate cancer bone metastasis. J Hematol Oncol. (2023) 16:46. doi: 10.1186/s13045-023-01442-4, PMID: 37138326 PMC10155318

[269] Yin C Wang M Wang Y Lin Q Lin K Du H . BHLHE22 drives the immunosuppressive bone tumor microenvironment and associated bone metastasis in prostate cancer. J Immunother Cancer. (2023) 11. doi: 10.1136/jitc-2022-005532, PMID: 36941015 PMC10030795

[270] Adegoke NA Gide TN Mao Y Quek C Patrick E Carlino MS . Classification of the tumor immune microenvironment and associations with outcomes in patients with metastatic melanoma treated with immunotherapies. J Immunother Cancer. (2023) 11. doi: 10.1136/jitc-2023-007144, PMID: 37865395 PMC10603328

[271] Fang S Ni H Zhang Q Dai J He S Min J . Integrated single-cell and bulk RNA sequencing analysis reveal immune-related biomarkers in postmenopausal osteoporosis. Heliyon. (2024) 10:e38022. doi: 10.1016/j.heliyon.2024.e38022, PMID: 39328516 PMC11425179

[272] Qu J Li B Qiu M Wang J Chen Z Li K . Discovery of immune-related diagnostic biomarkers and construction of diagnostic model in varies polycystic ovary syndrome. Arch Gynecol Obstet. (2022) 306:1607–15. doi: 10.1007/s00404-022-06686-y, PMID: 35904610

[273] Zhang B Pang Y . Exploring the genetic profiles linked to senescence in thyroid tumors: insights on predicting disease progression and immune responses. Front Oncol. (2025) 15:1545656. doi: 10.3389/fonc.2025.1545656, PMID: 39980566 PMC11839597

[274] Xie X Liu H Wan K Li J Qi P . The gut microbiota in osteoporosis: dual roles and therapeutic prospects. Front Immunol. (2025) 16:1617459. doi: 10.3389/fimmu.2025.1617459, PMID: 40959063 PMC12434086

